# Supplementary material for: Eribulin induces micronuclei and enhances the nuclear localization of cGAS in triple-negative breast cancer cells
Source: Sci Rep. 2024 Jun 19;14:14146. doi: 10.1038/s41598-024-64651-y (PMC11187130; doi:10.1038/s41598-024-64651-y)
Supplement: Supplementary file 1 — Supplementary Information 1. [file 41598_2024_64651_MOESM1_ESM.pdf]

# Original file of repeated experiments

We have performed at least triplicate of all experiments to confirm our conclusions.

**MM231** 1st

**cGAS**

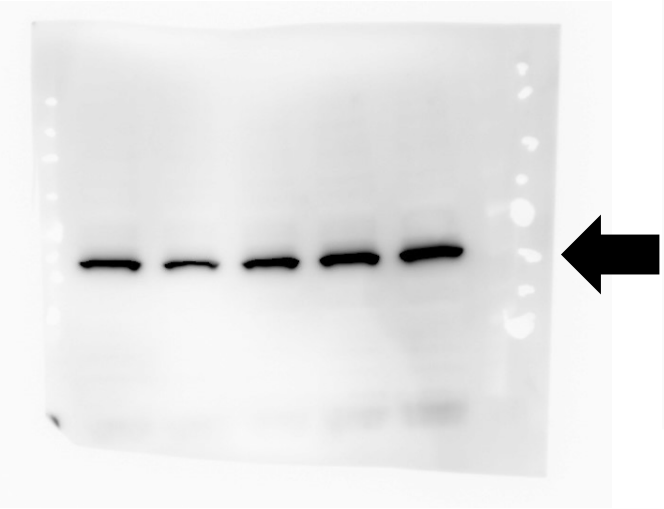

**STING**

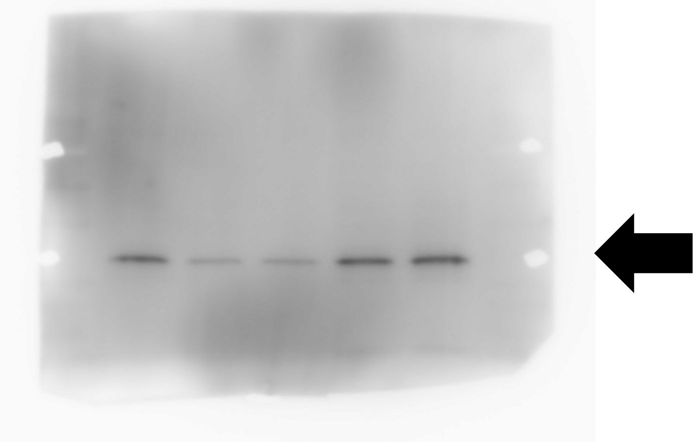

**IFNβ**

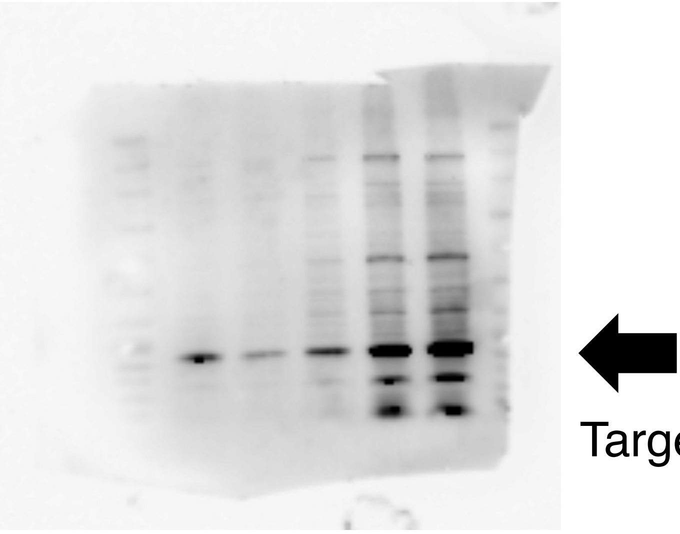

Target band

**Vinculin**

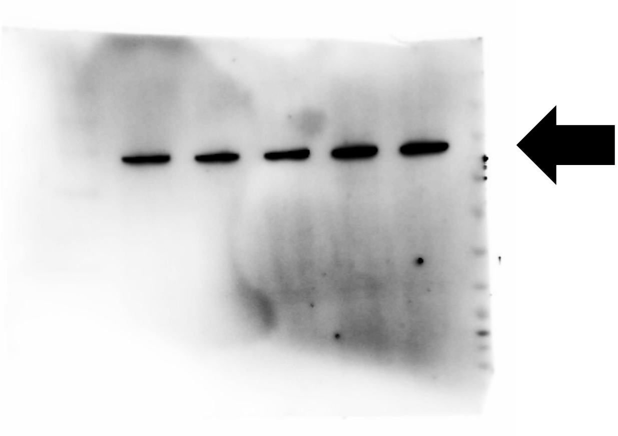

Uncropped Gels and Blots images in MM231 were shown.

**Figure 2f**

**MM231**

**cGAS**

| Short exposure | long exposure |
|---|---|

**2nd**

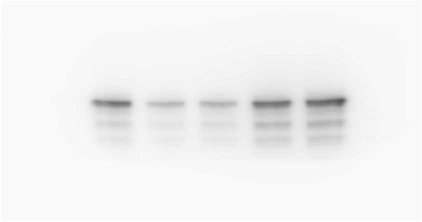

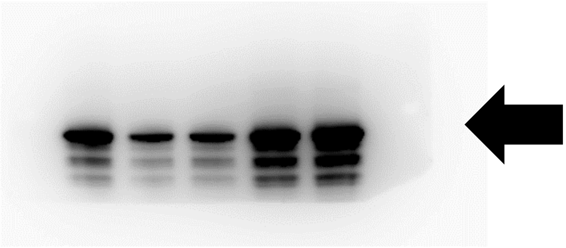

**3rd**

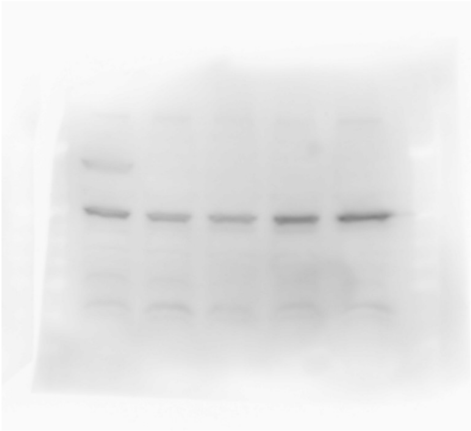

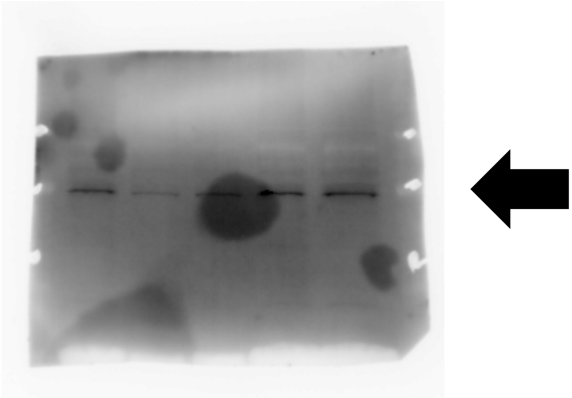

Uncropped Gels and Blots images in MM231 were shown.

**Figure 2f**

**MM231**

**STING**

**2nd**

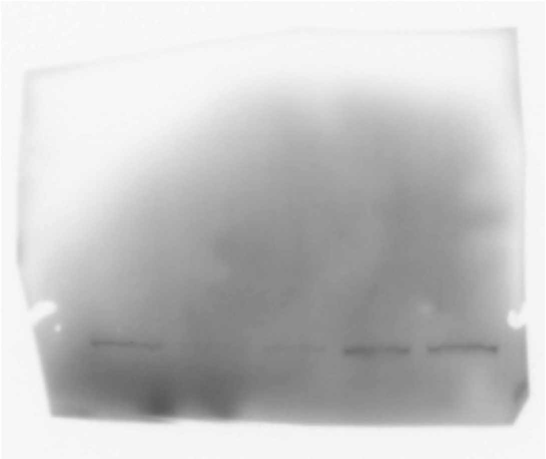

Target band

**3rd**

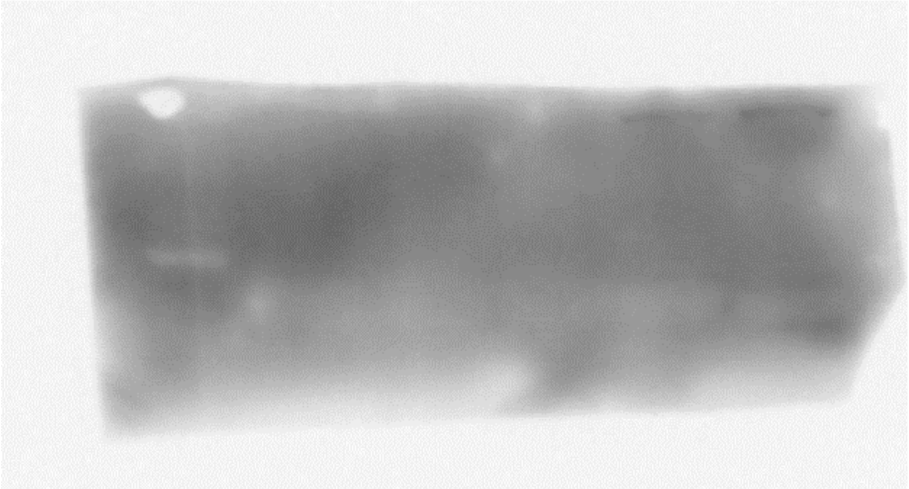

Target band

Uncropped Gels and Blots images in MM231 were shown.

**Figure 2f**

**MM231**

**IFNβ**

**2nd**

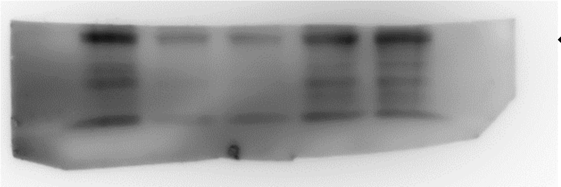

Target band

**3rd**

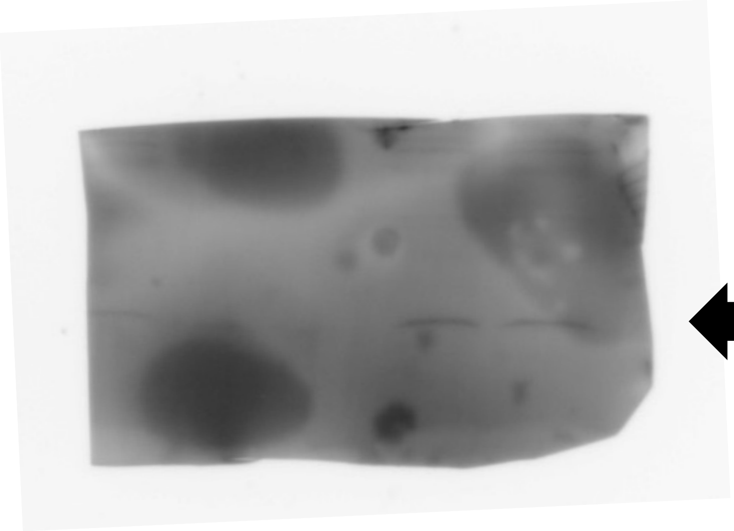

Target band

Uncropped Gels and Blots images in MM231 were shown.

**Figure 2f**

**MM231**

**Vinculin**

**2nd**

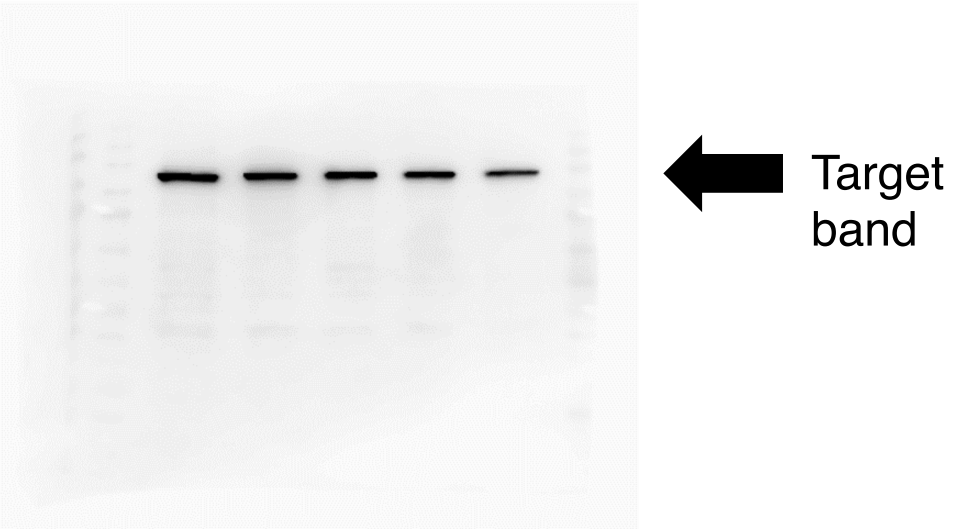



**3rd**

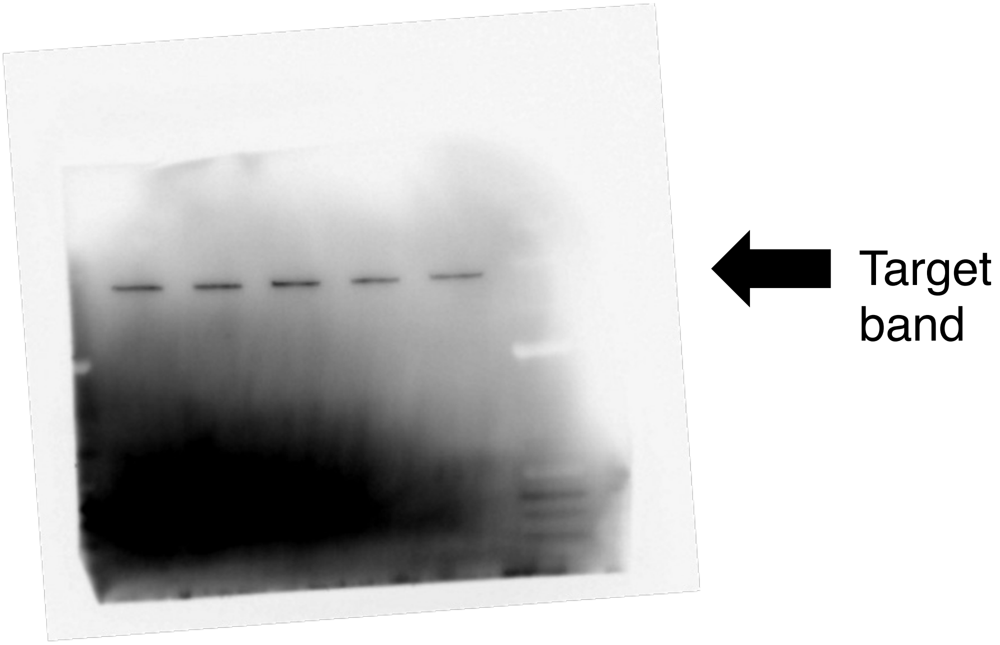



Uncropped Gels and Blots images in MM231 were shown.

**Figure 2f**

**MM231**

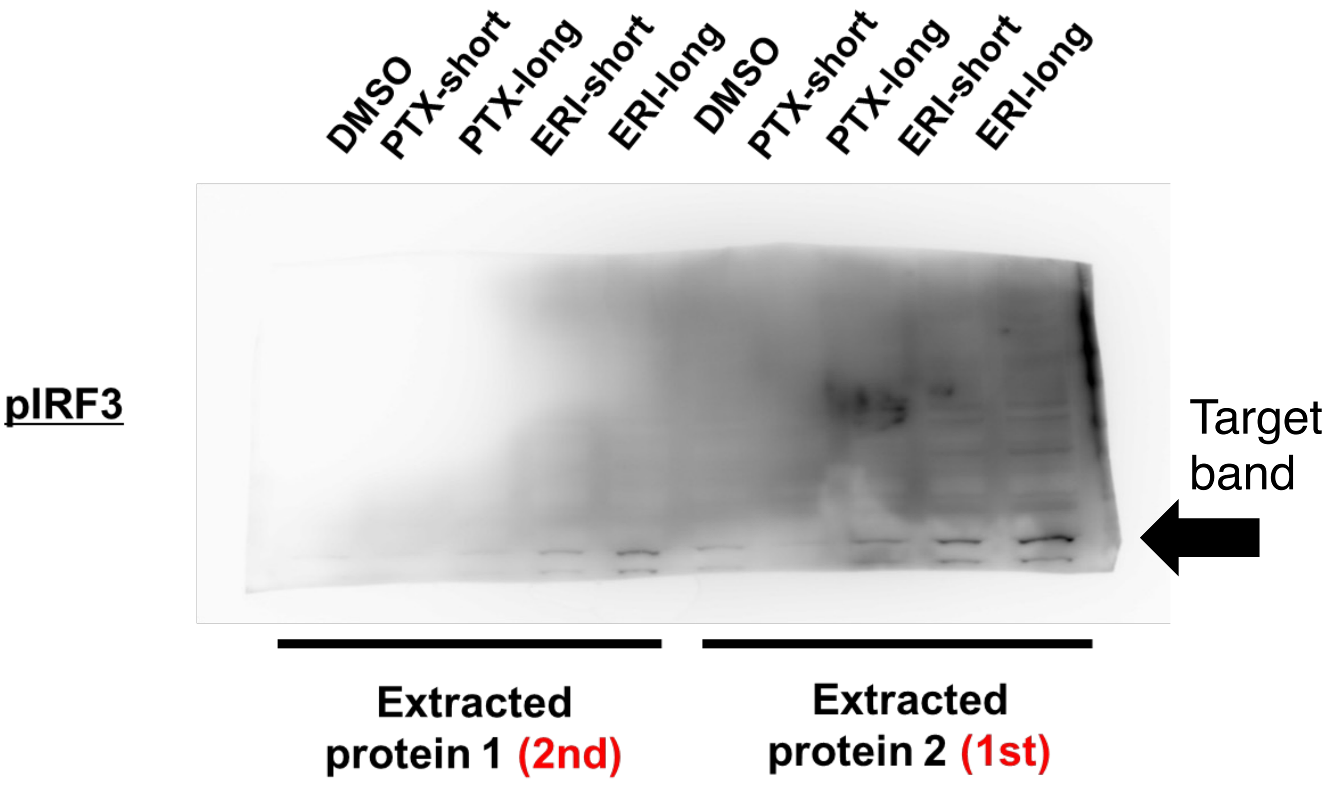


Protein extraction was performed from MM231 cells twice independently and pIRF3 expression was evaluated. The 5 lanes on the left side are ”extracted protein 1” and the 5 lanes on the right side are ”extracted protein 2”. The "extracted protein 2" was used as Figure2f.

Uncropped Gels and Blots images in MM231 were shown.

**Figure 2f**

**MM231**

**pIRF3**

**3rd**

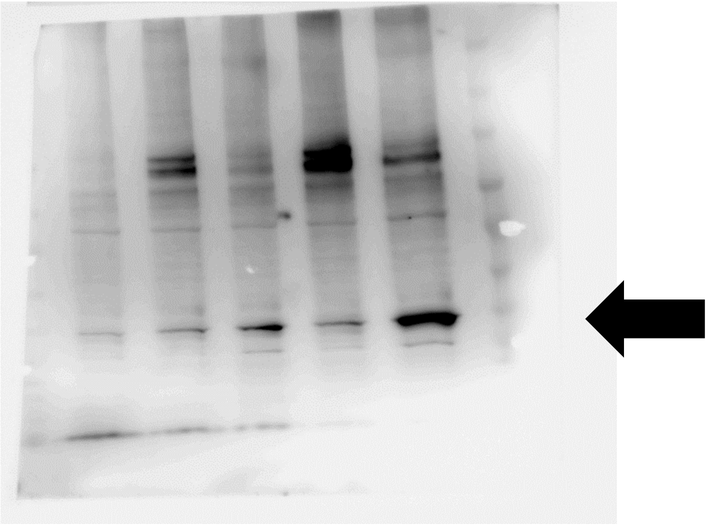


Uncropped Gels and Blots images in MM231 were shown.

**Figure 2f**

**RPE1**

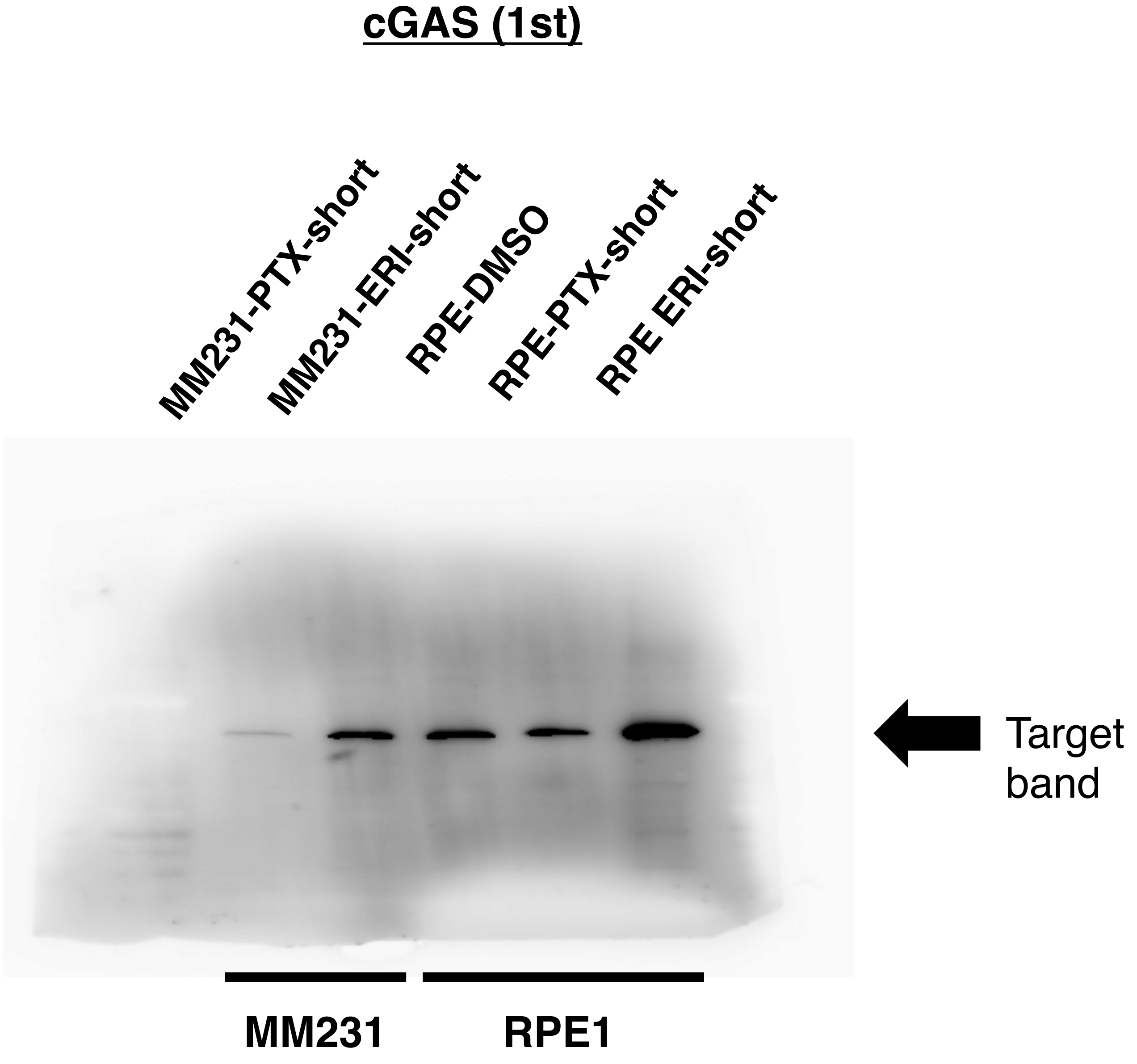


MM231-PTX-short and MM231-ERI-short were also checked whether the bands were detected. Therefore, there are blots of MM231 at the left of the target bands.

Uncropped Gels and Blots images in RPE1 were shown.

**Figure 2f**

**RPE1**

**cGAS**

**2nd**

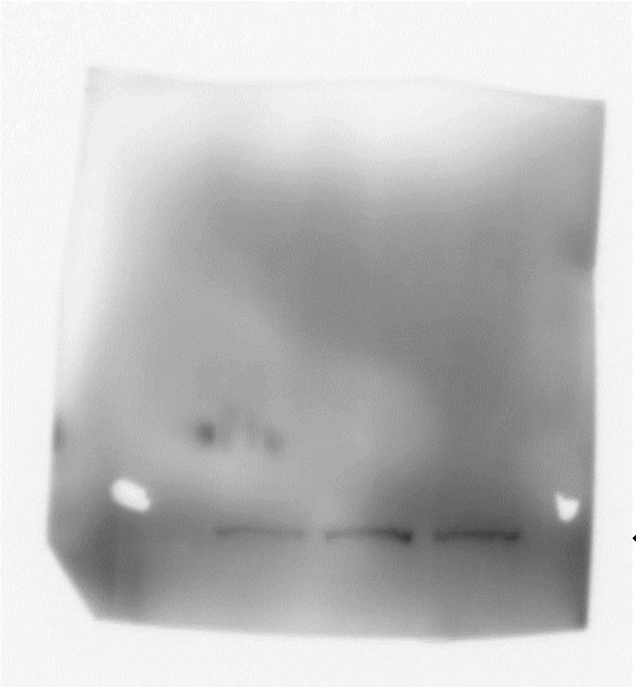

Target band

**3rd**

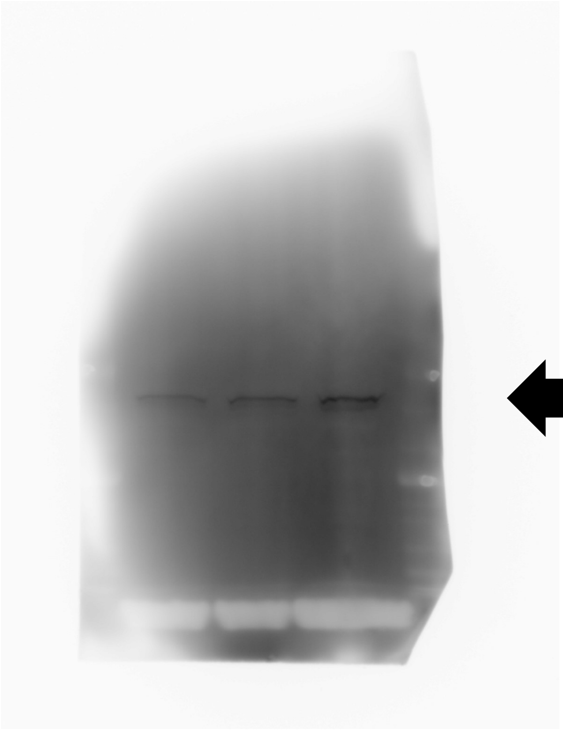

Target band

Uncropped Gels and Blots images in RPE1 were shown.

**Figure 2f**

**RPE1**

**STING (1st)**

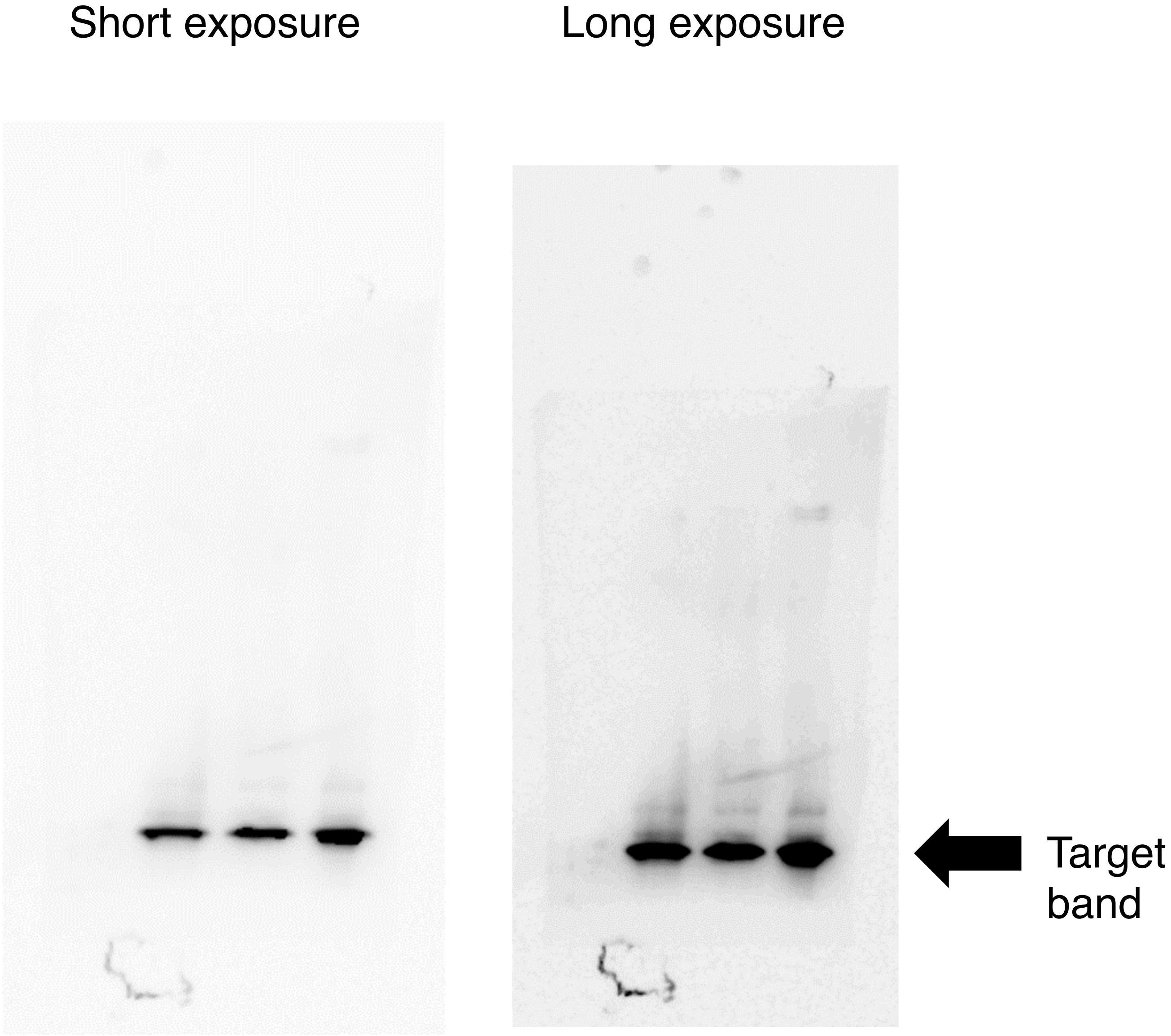


Uncropped Gels and Blots images in RPE1 were shown.

**Figure 2f**

**RPE1**

**STING**

Short exposure

Long exposure

**2nd**

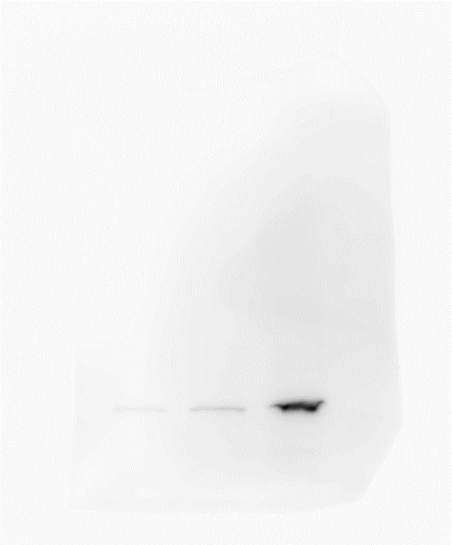

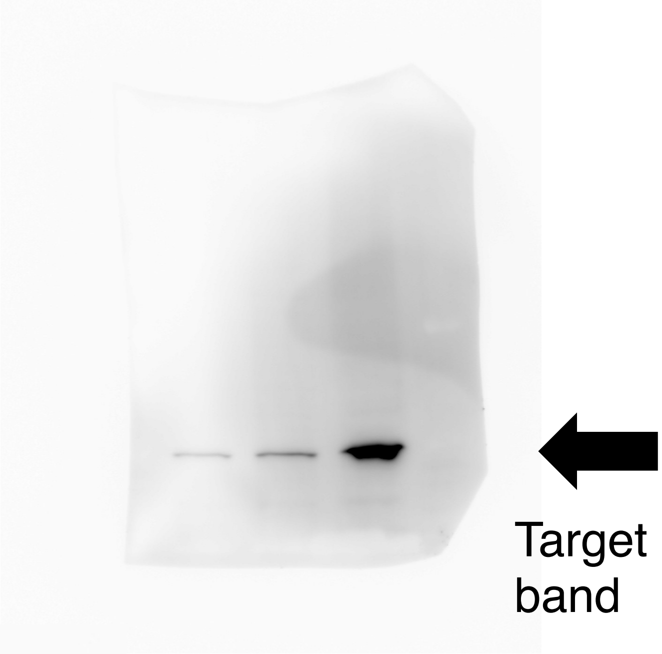

Target band

**3rd**

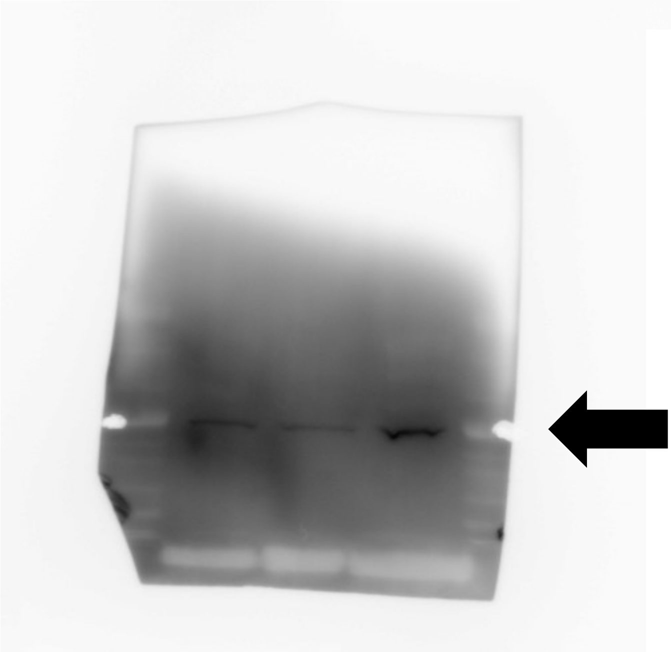

Uncropped Gels and Blots images in RPE1 were shown.

**Figure 2f**

**RPE1**

**pIRF3**

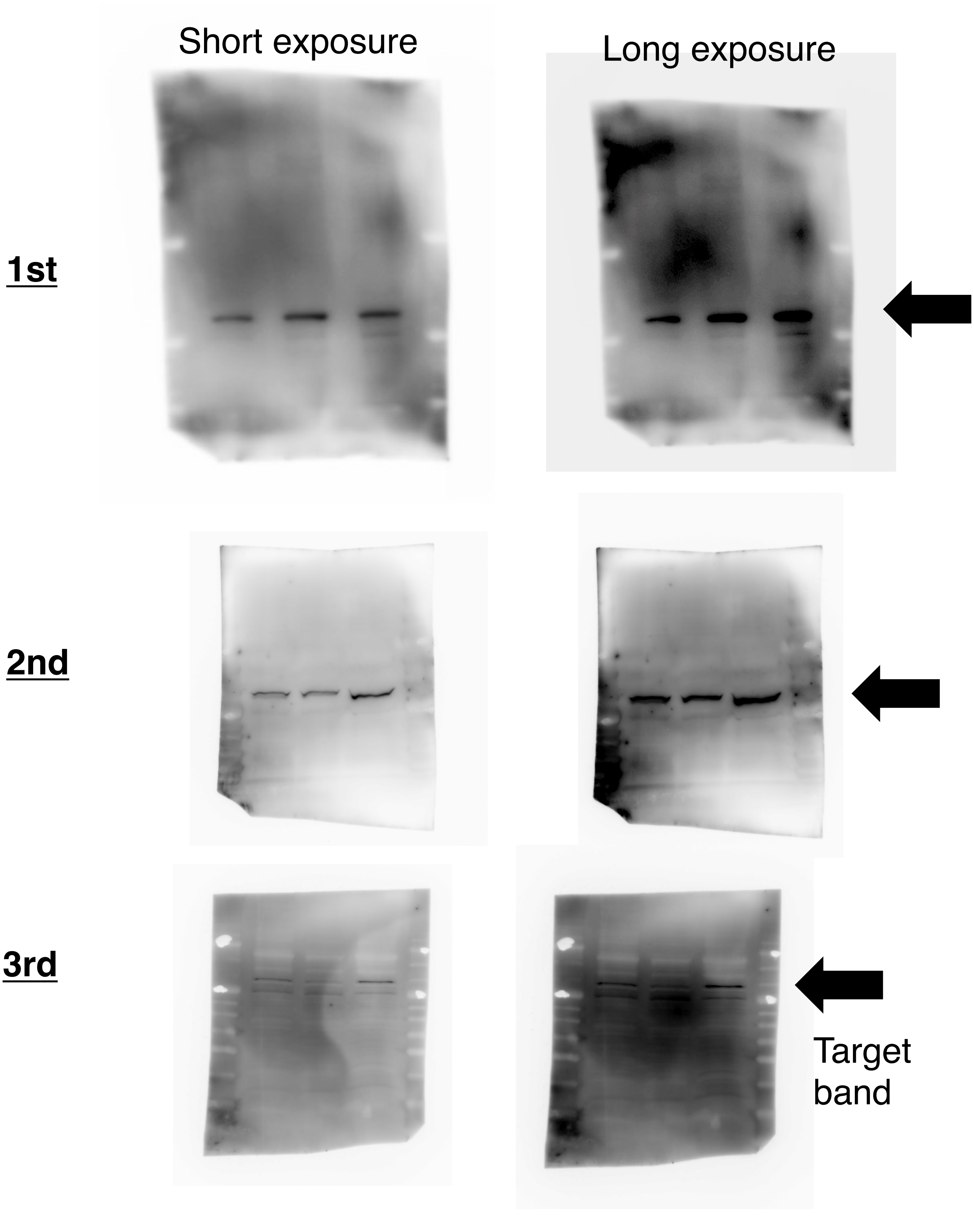


Uncropped Gels and Blots images in RPE1 were shown.

**Figure 2f**

**RPE1**

**<u>IFNβ (1st)</u>**

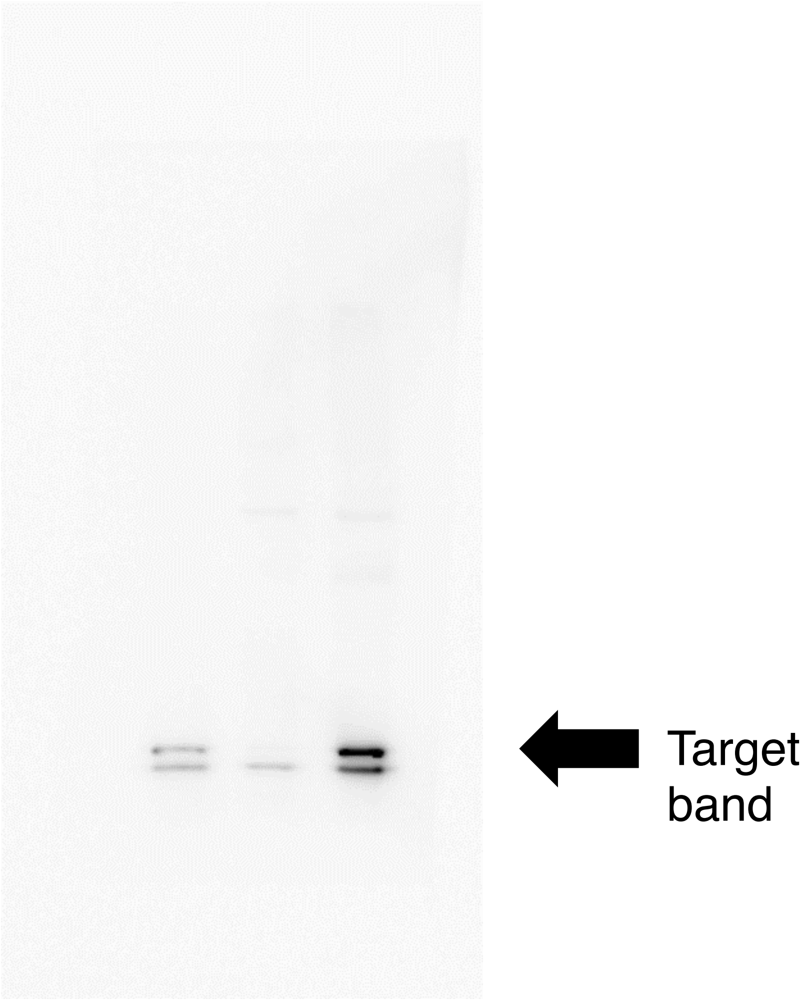


Uncropped Gels and Blots images in RPE1 were shown.

**Figure 2f**

**RPE1**

**IFNβ**

**2nd**

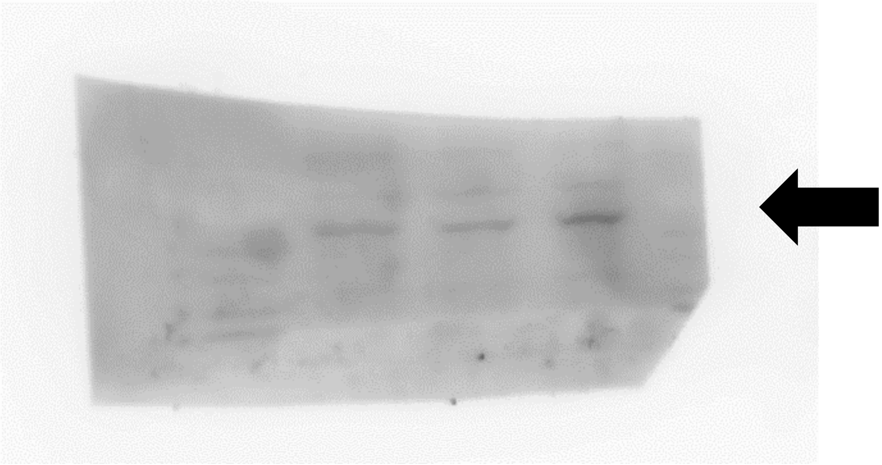

**3rd**

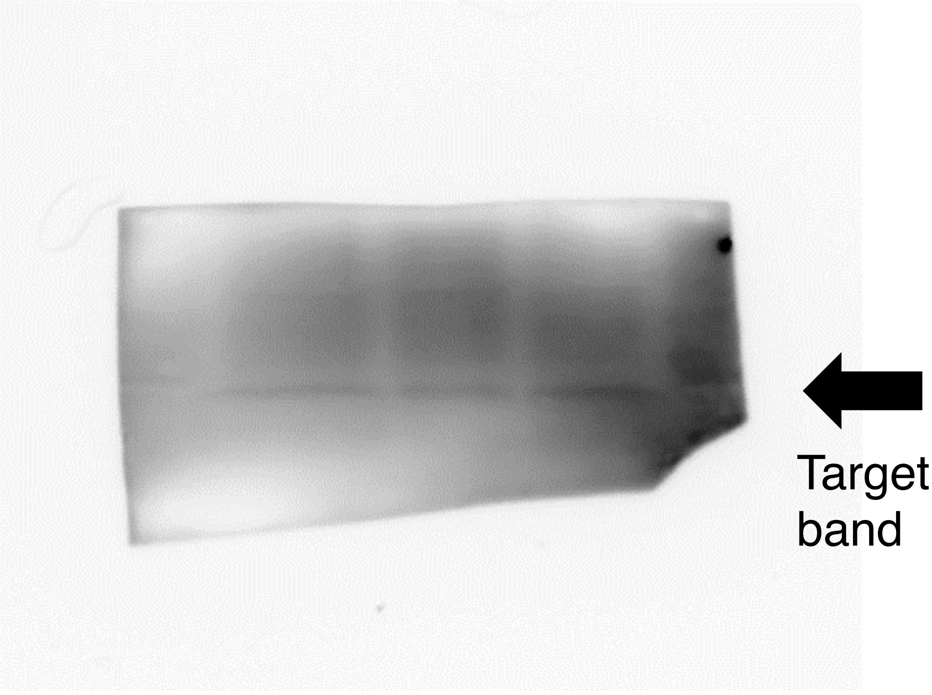

Target band

Uncropped Gels and Blots images in RPE1 were shown.

**Figure 2f**

**RPE1**

**Vinculin**

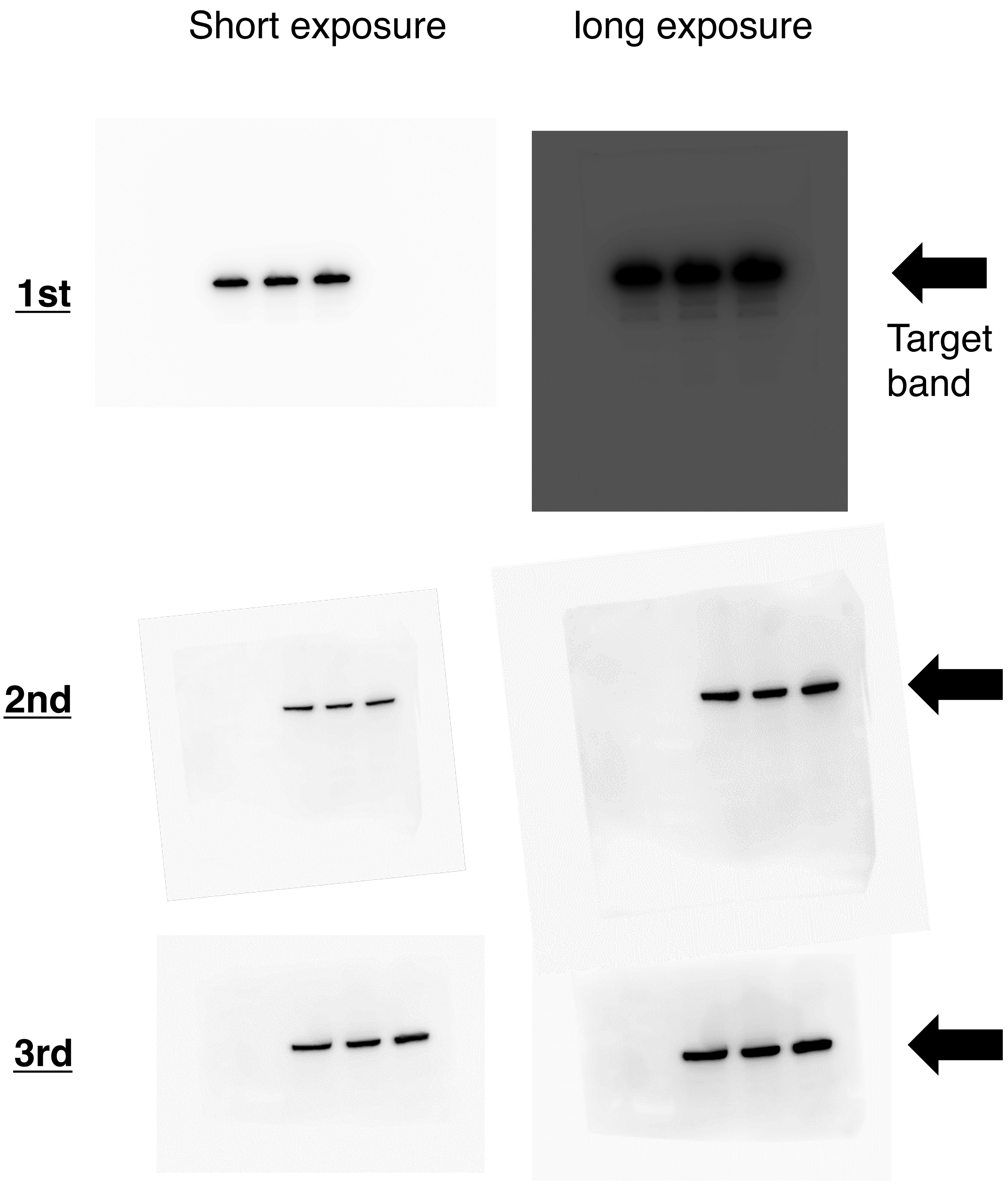


Uncropped Gels and Blots images in RPE1 were shown.

**Figure 2f**

**1st**

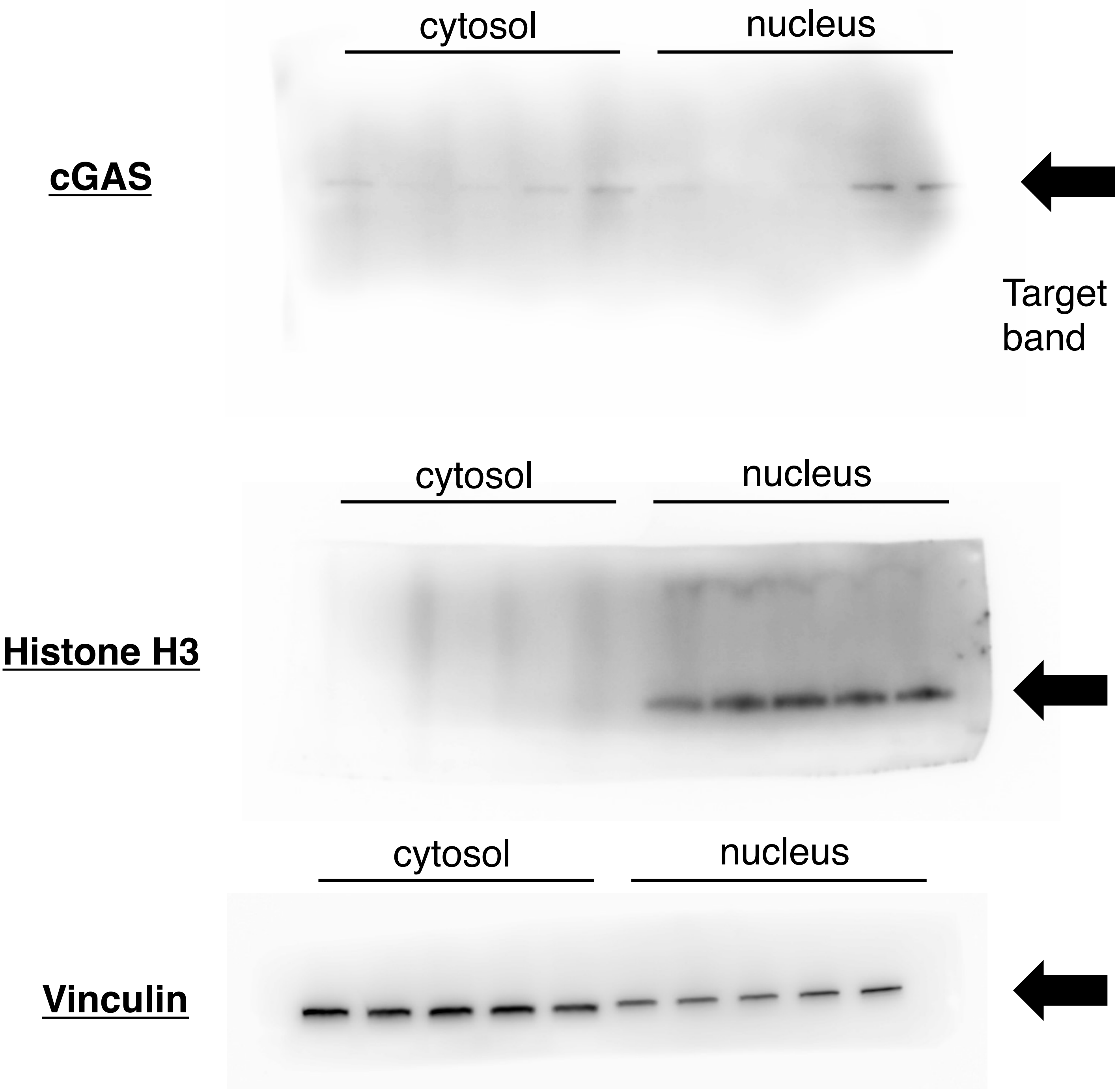


The order of the cytoplasmic and nuclear fractions is reversed in the 2nd and 3rd procedures.

Uncropped Gels and Blots images of Figure 2g were shown.

**Figure 2g**

**cGAS**

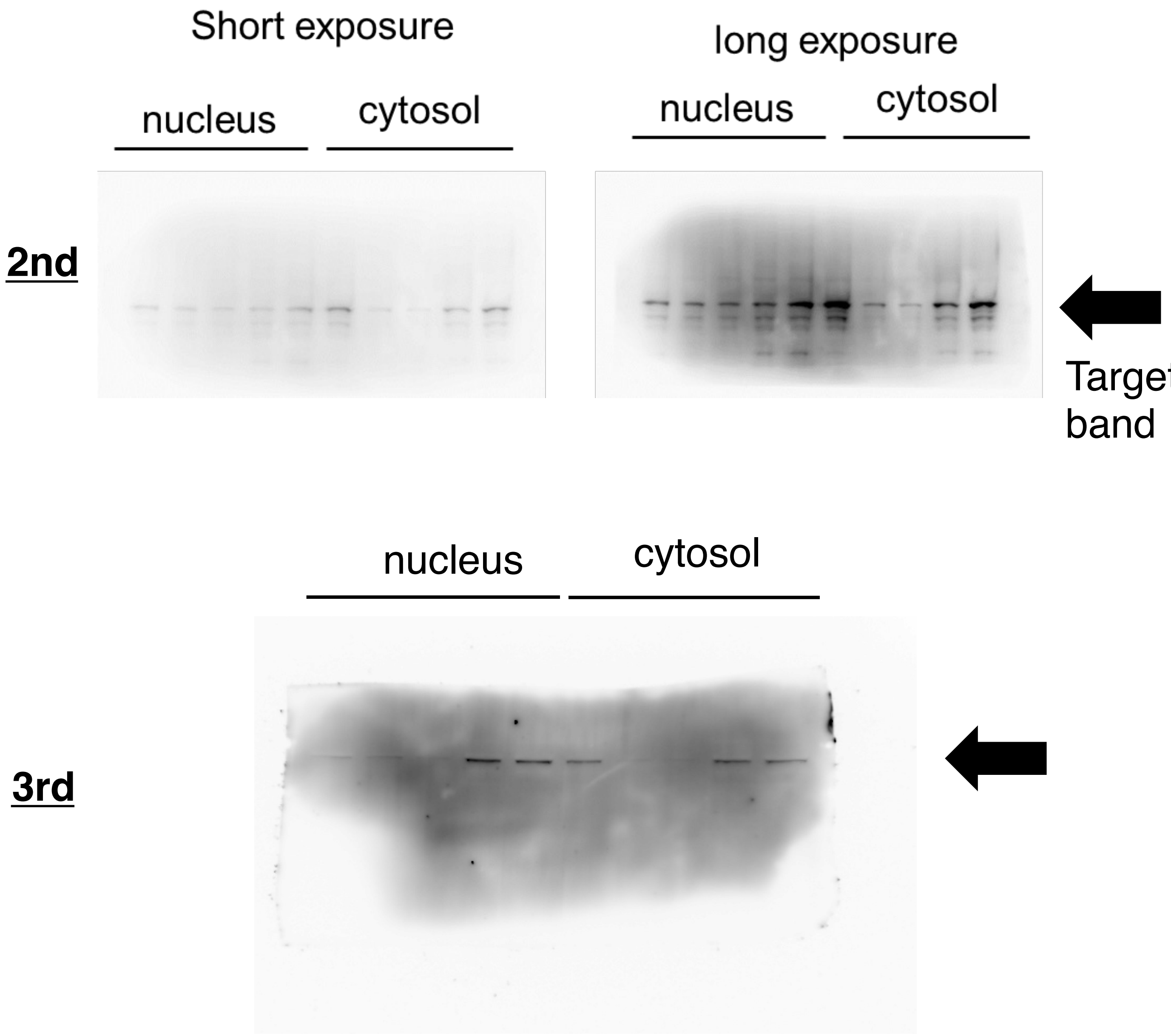


The order of the cytoplasmic and nuclear fractions is reversed in the 2nd and 3rd procedures.

Uncropped Gels and Blots images of Figure 2g were shown.

**Figure 2g**

## Histone H3

**2nd**

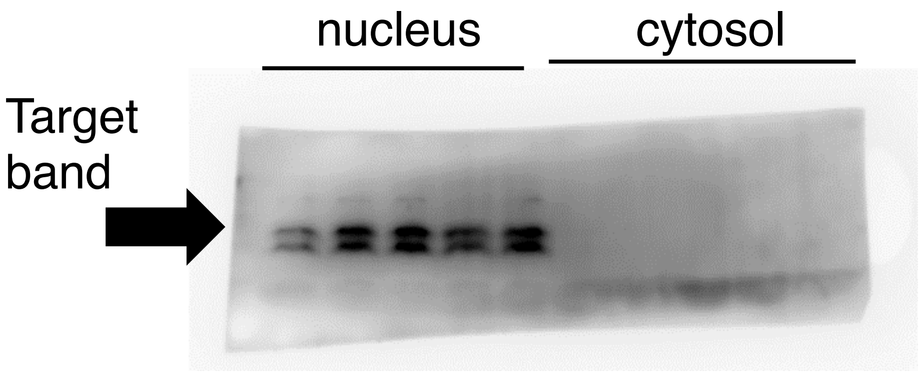


**3rd**

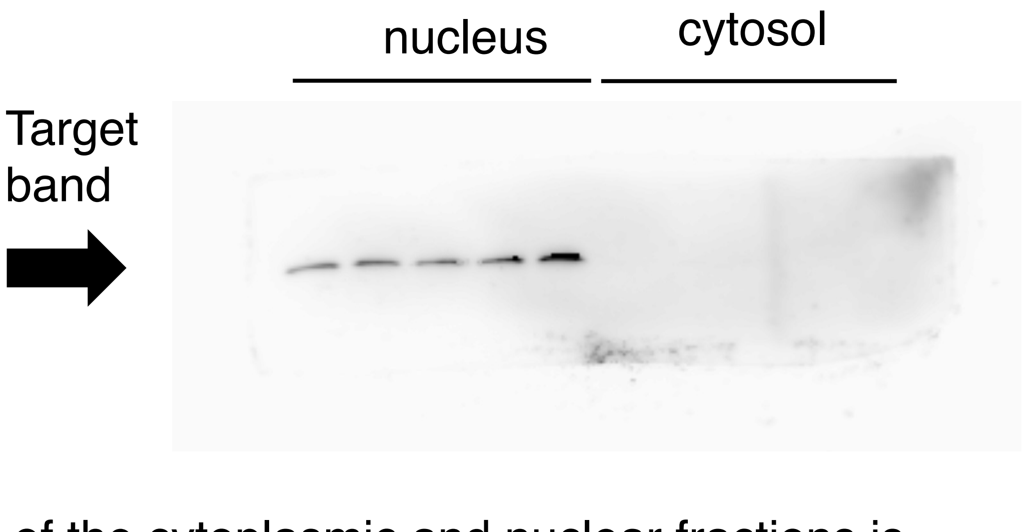


The order of the cytoplasmic and nuclear fractions is reversed in the 2nd and 3rd procedures.

Uncropped Gels and Blots images of Figure 2g were shown.

**Figure 2g**

## Vinculin

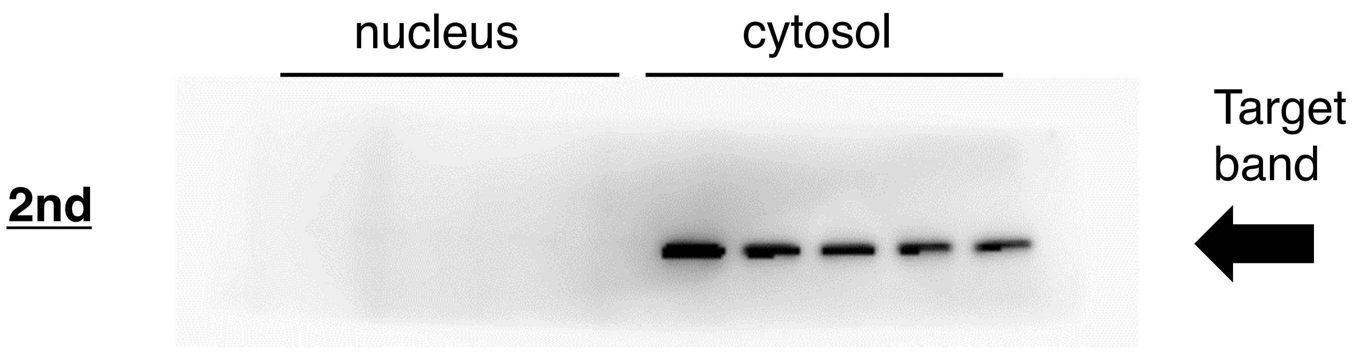


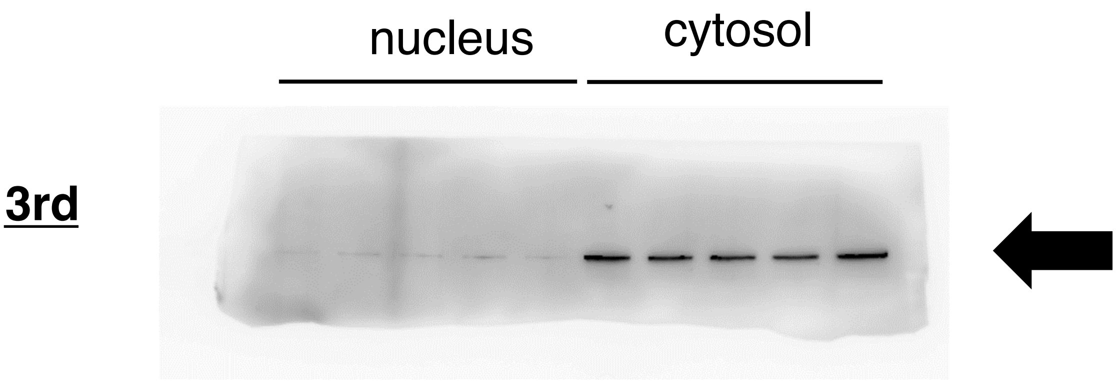


The order of the cytoplasmic and nuclear fractions is reversed in the 2nd and 3rd procedures.

Uncropped Gels and Blots images of Figure 2g were shown.

**Figure 2g**

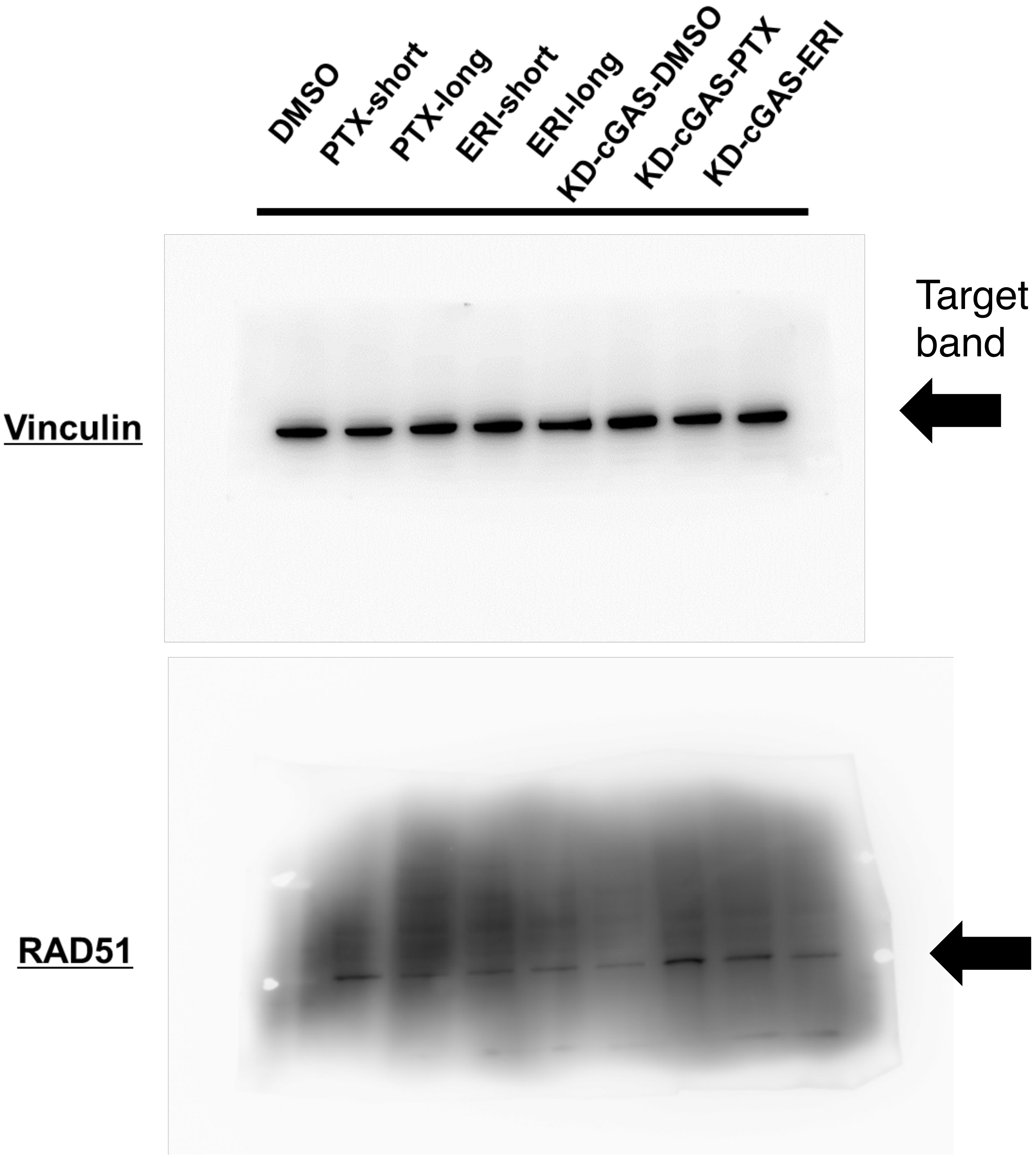


Uncropped Gels and Blots images of Figure 3c were shown.

**Figure 3c**

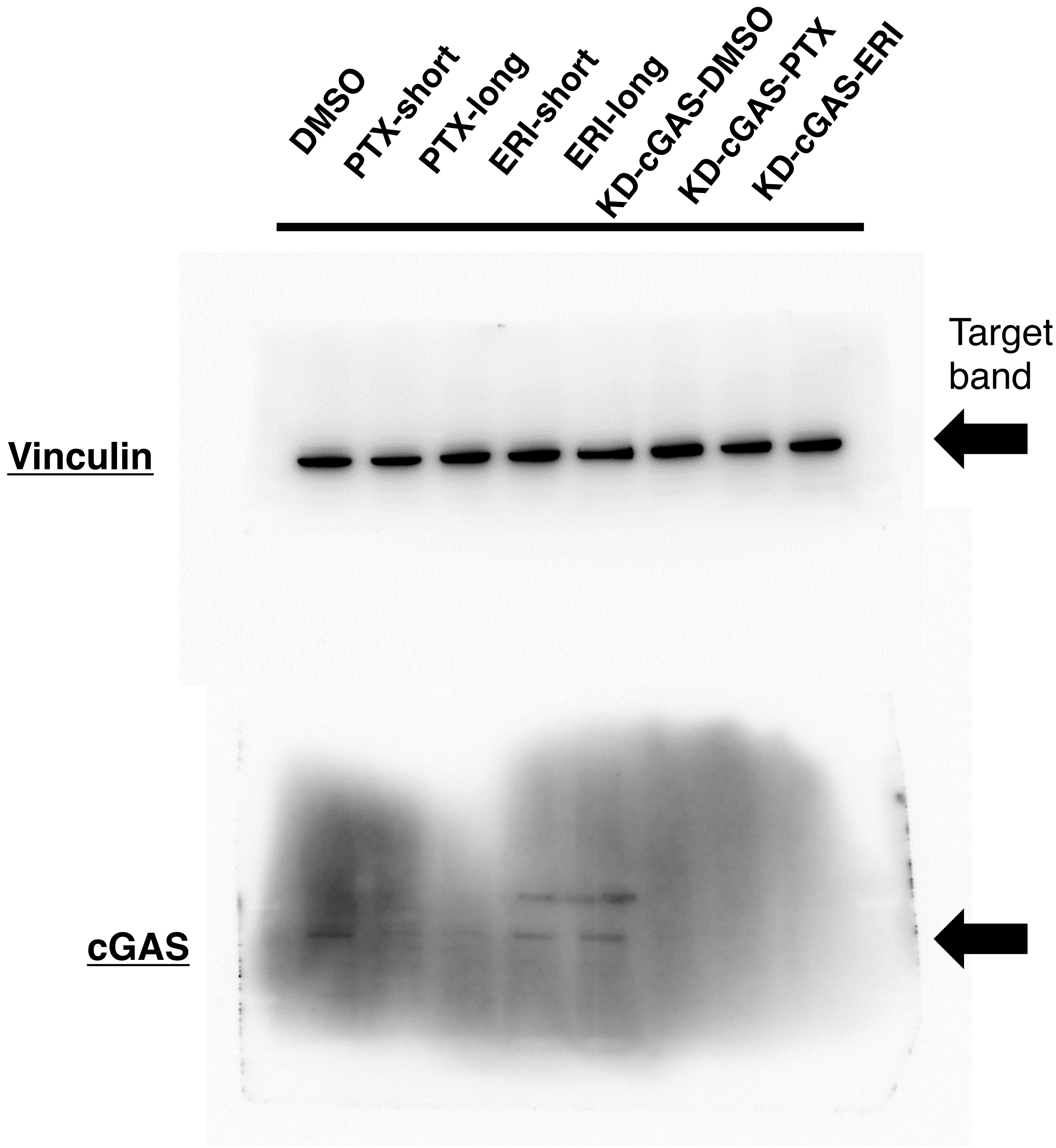


Uncropped Gels and Blots images of Figure 3c were shown.

**Figure 3c**

**Vinculin**

**2nd**

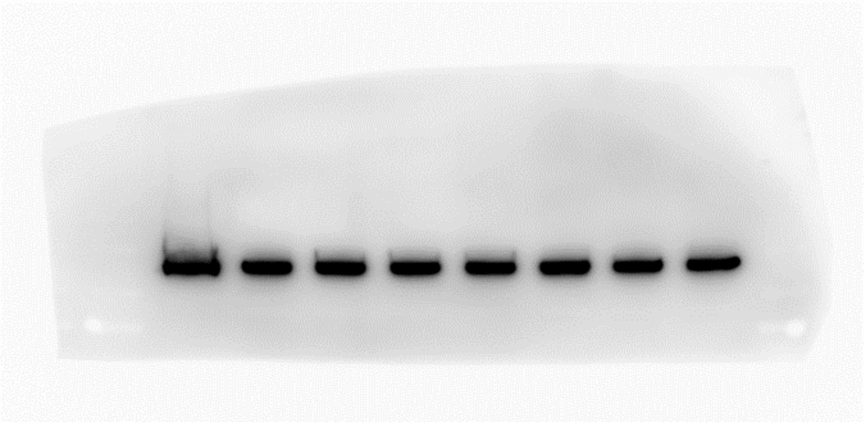

Target band

**3rd**

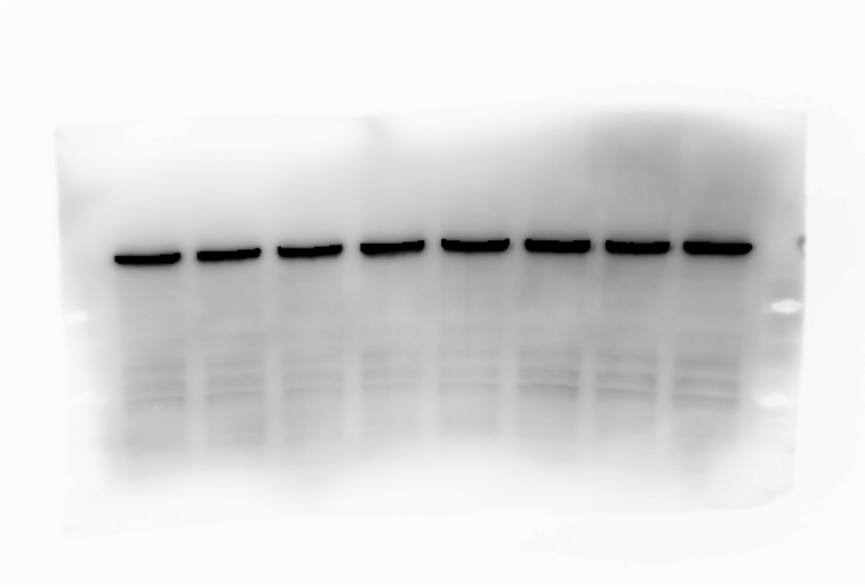

Uncropped Gels and Blots images of Figure 3c were shown.

**Figure 3c**

**RAD51**

Short exposure

long exposure

**2nd**

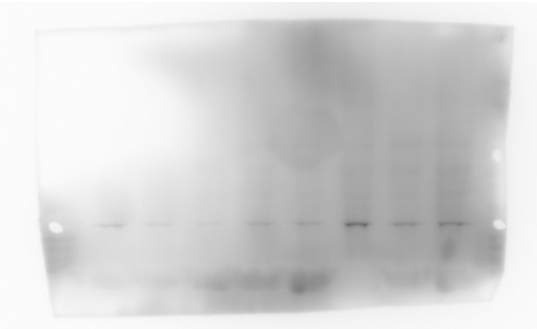

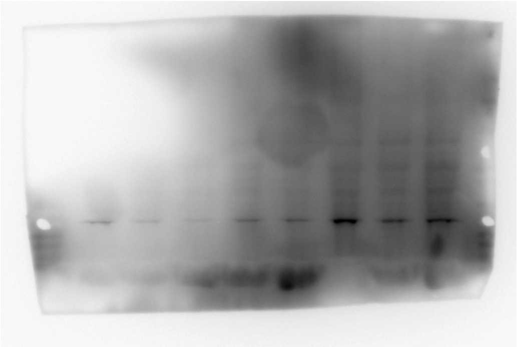

Target band

**3rd**

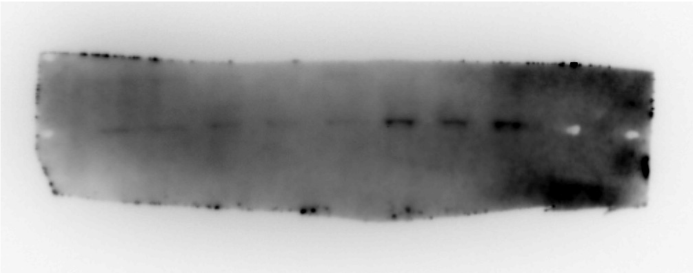

Uncropped Gels and Blots images of Figure 3c were shown.

**Figure 3c**

## cGAS

**2nd**

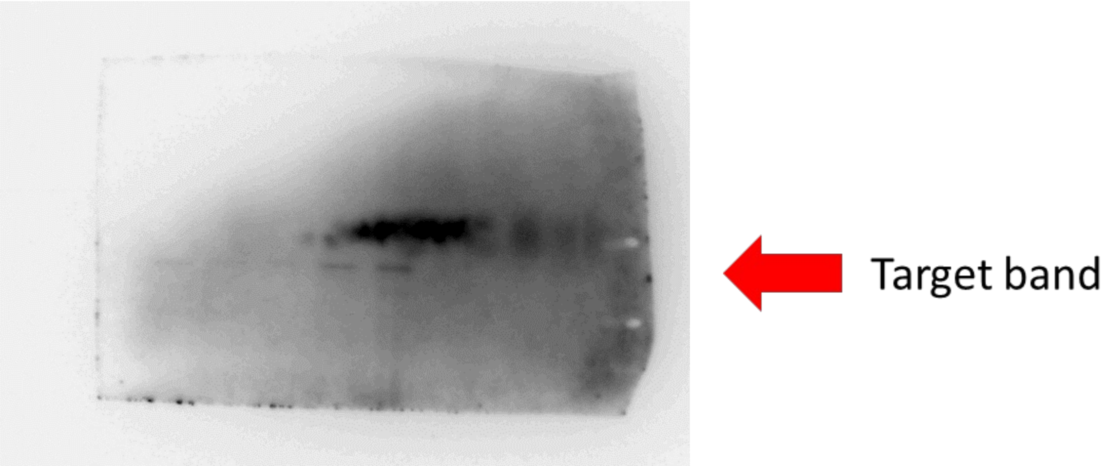


**3rd**

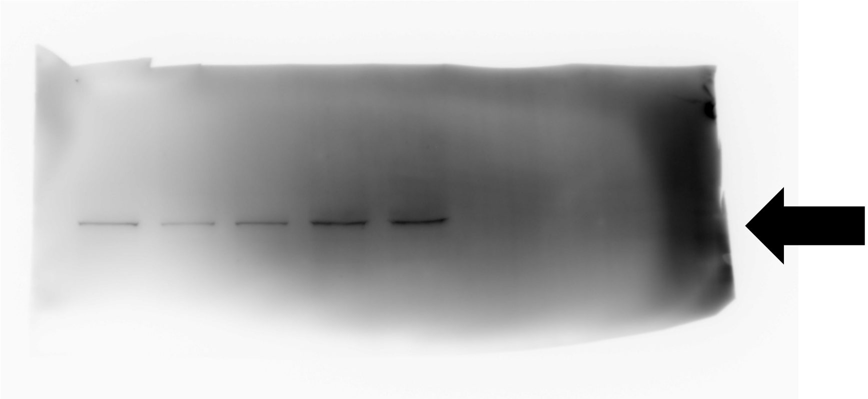

Uncropped Gels and Blots images of Figure 2g were shown.

**Figure 3c**

a

**cGAS**

**1st**

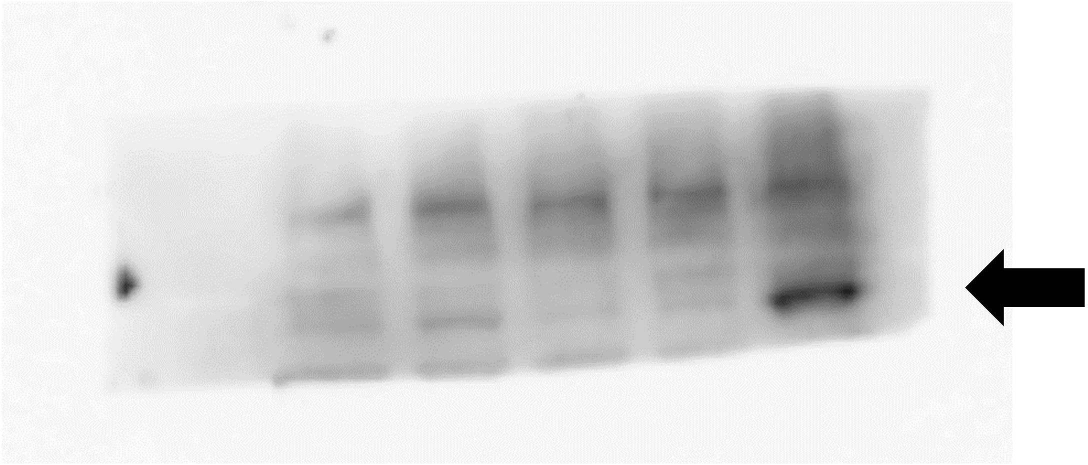

**Histone H3**

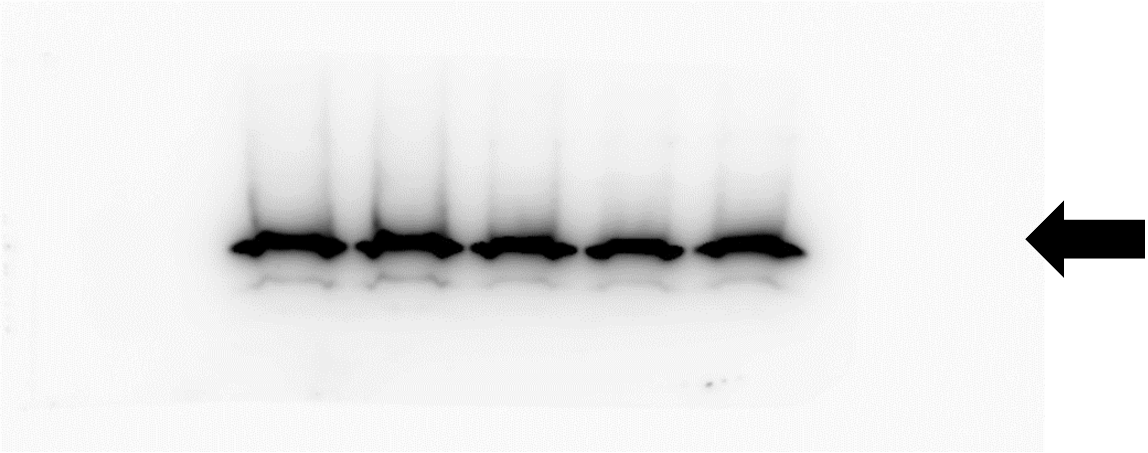

Uncropped Gels and Blots images of Supplementary Figure 2 were shown.

**Supplementary Figure 2**

**cGAS**

**2nd**

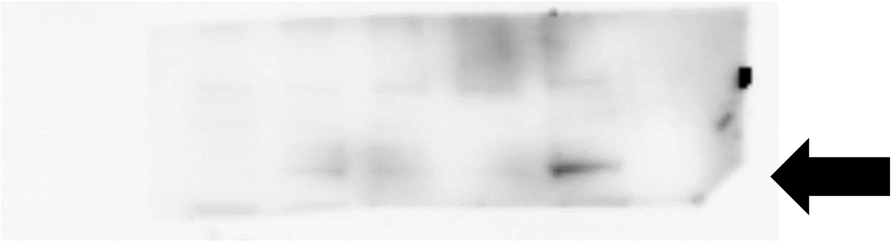

**3rd**

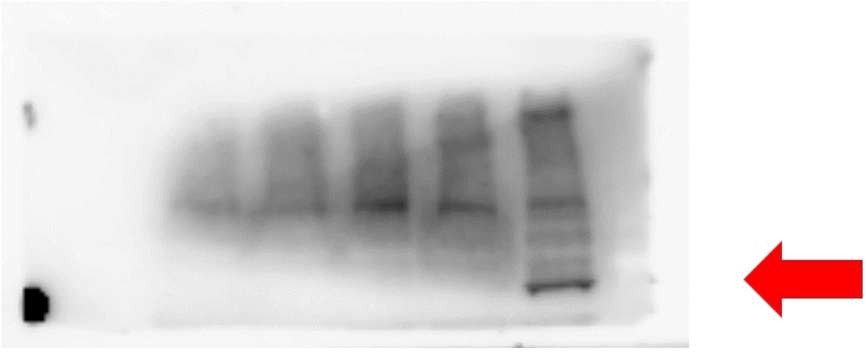

Target band

Uncropped Gels and Blots images of Supplementary Figure 2 were shown.

**Supplementary Figure 2**

**Histone H3**

**2nd**

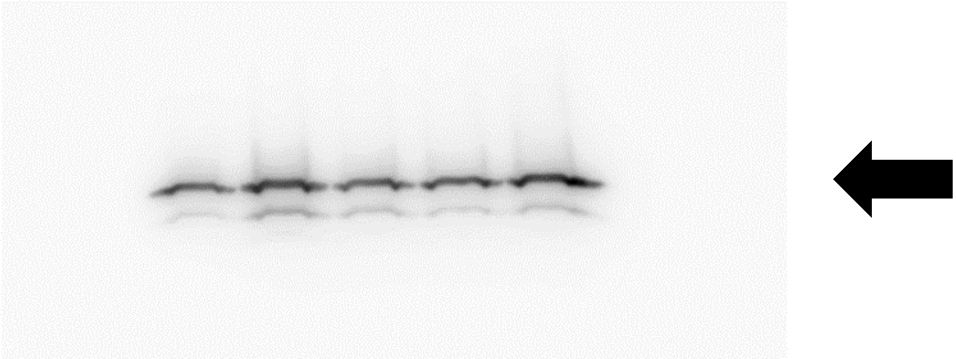

**3rd**

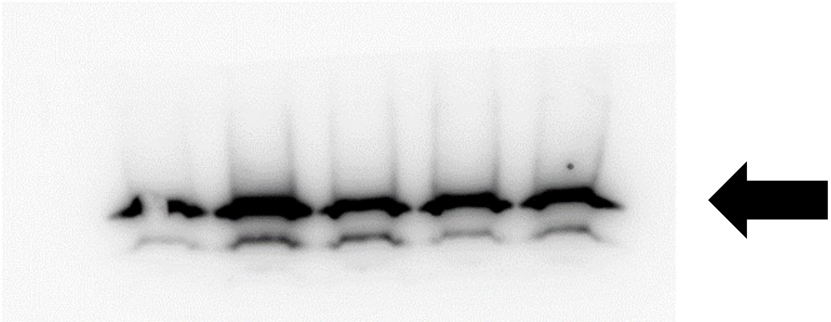

Uncropped Gels and Blots images of Supplementary Fig 2 were shown.

**Supplementary Figure 2**

# Supplemental figure regarding what reviwer pointed out

## Supplementary Figure 2c

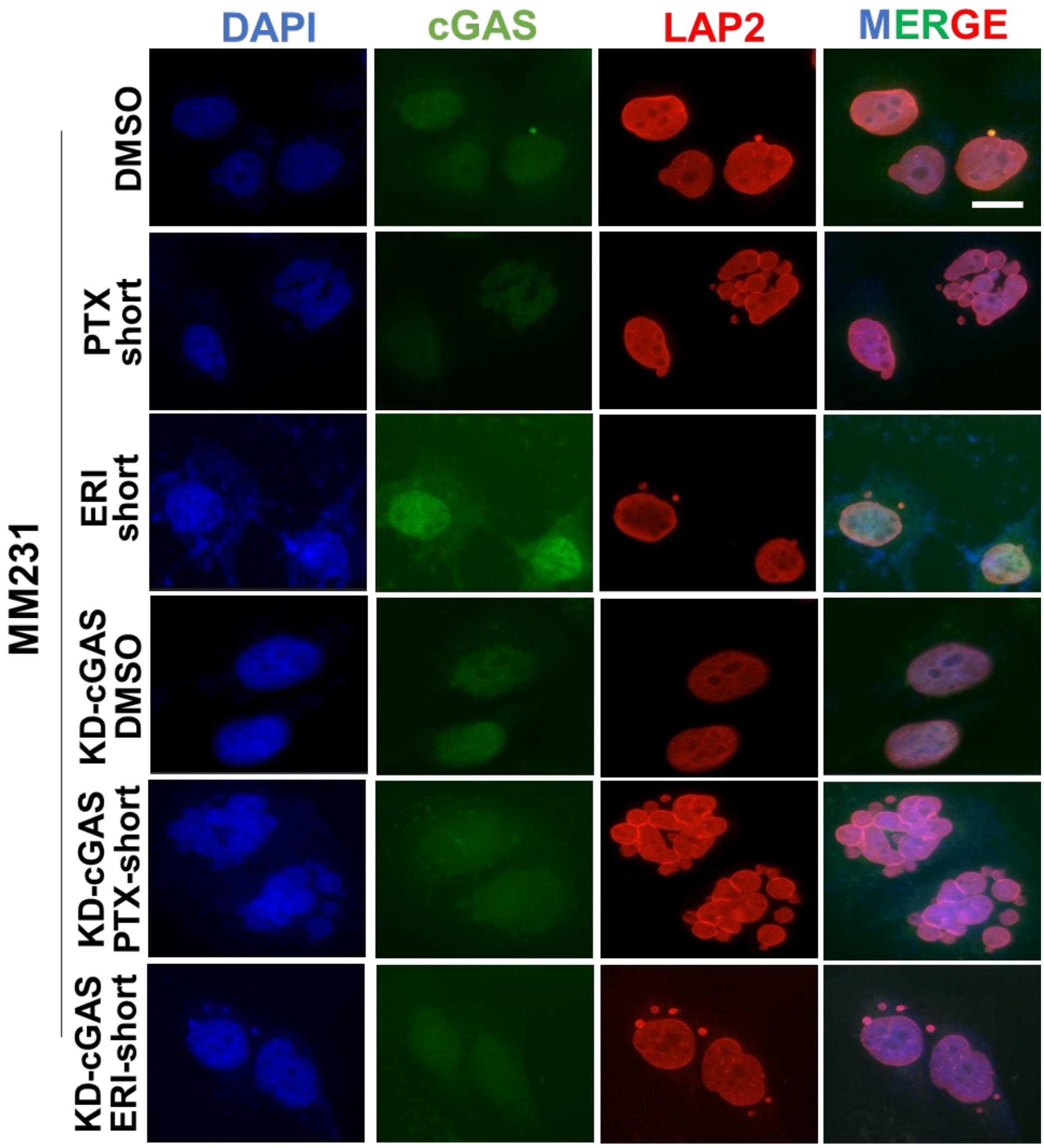


cGAS staining in Figure 2 seems to be a cGAS-specific finding because the staining of the cells seems to disappear when cGAS is knocked down as shown in **supplementary Fig.2c**.

# Supplementary Figure

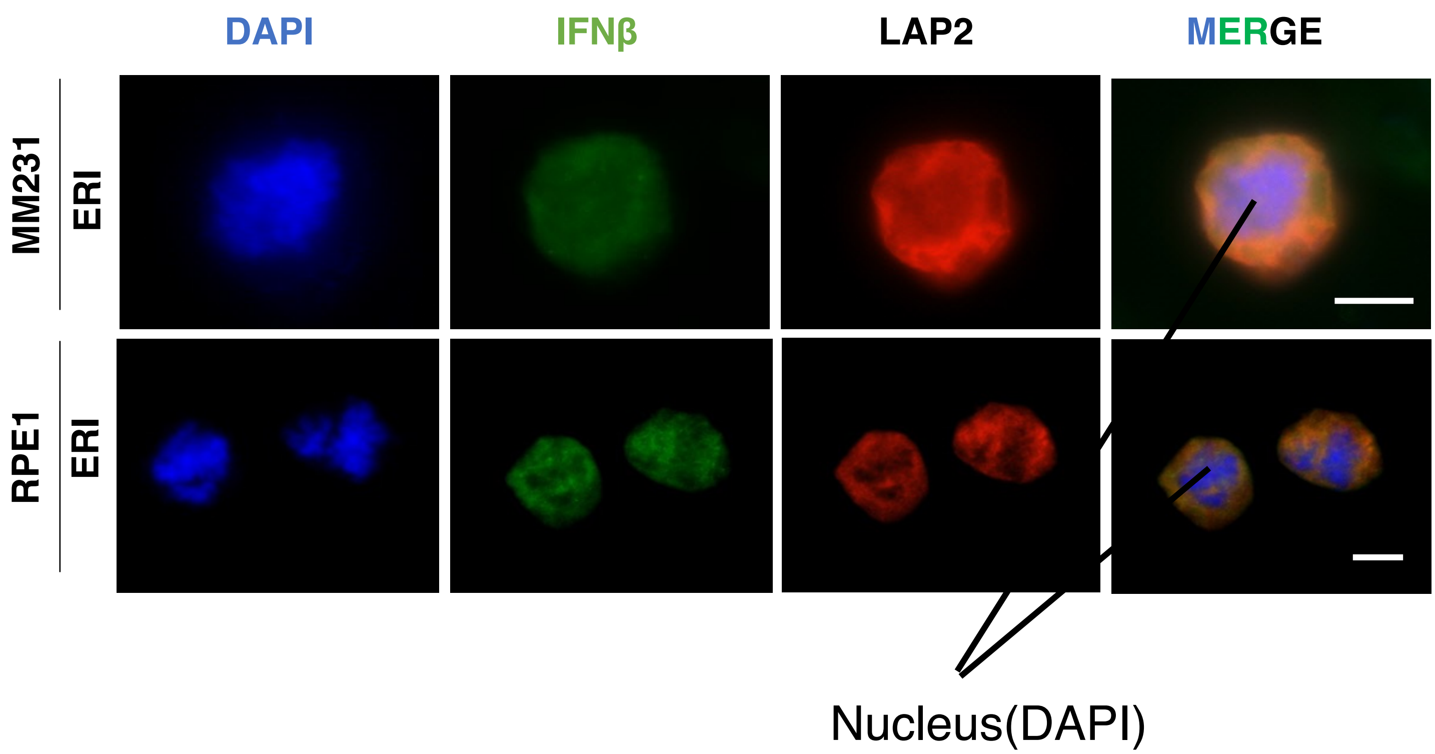


These are part of magnified images of the IFNβ-stained cells in **Figure 2b and 2d**. IFNβ was not stained in the nucleus but stained in the cytosol. The white bars are 200μm.

Additional information on figure 2b and 2d

# Figure 2c

**Figure 2c (with higher exposure)**

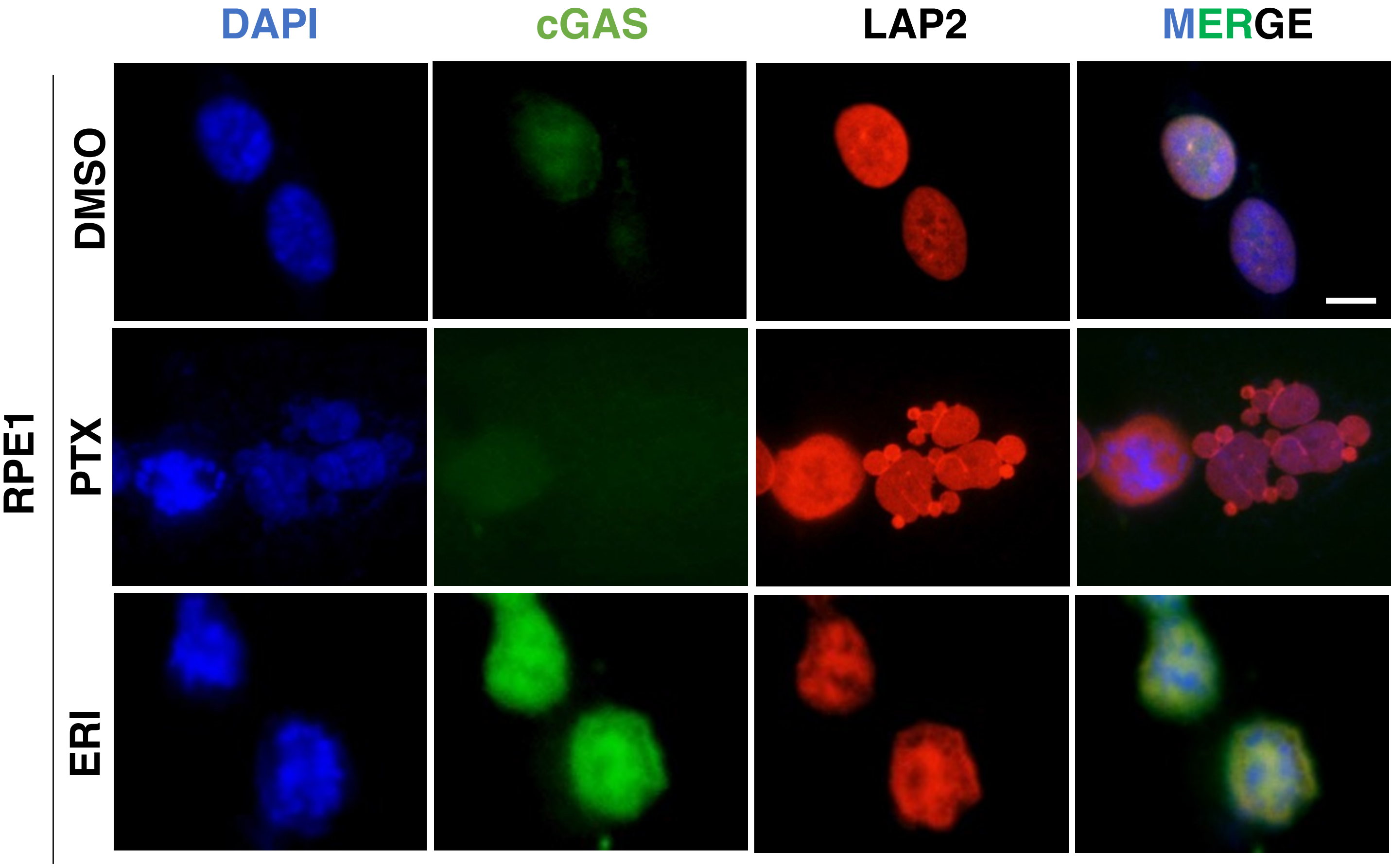


An image gain-adjusted figure is attached after the images were taken (above).

We would like to change Figure 2c to these photos.

**Figure 2c (before revise: lower exposure)**

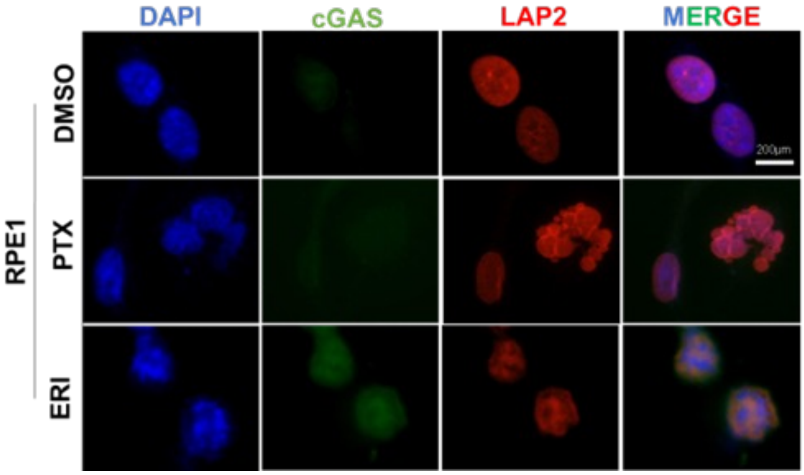

# Figure 2d

## Figure 2d (with higher exposure)

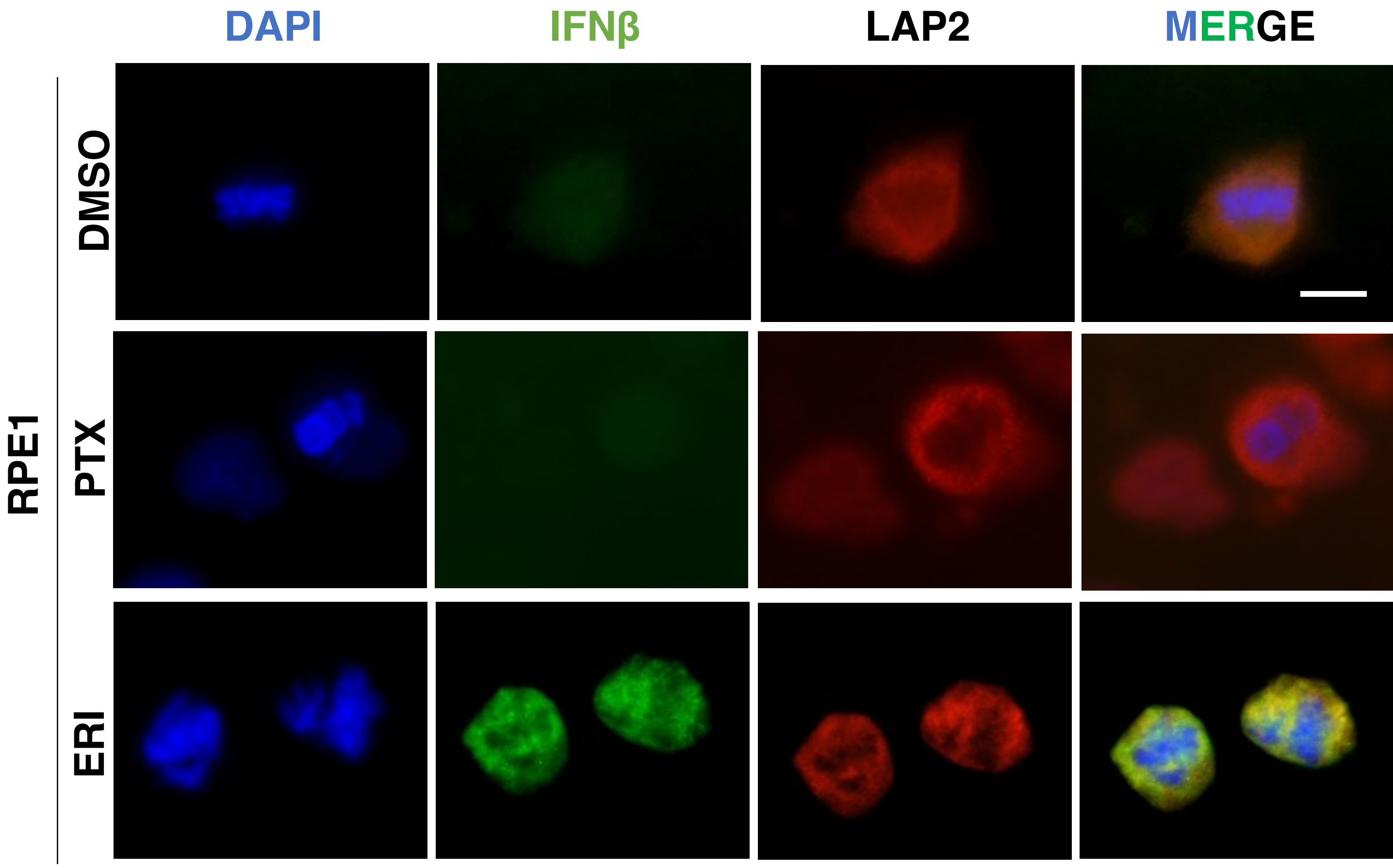


An image gain-adjusted figure is attached after the images were taken (above).

We would like to change Figure 2d to these photos.

## Figure 2d (before revise: lower exposure)

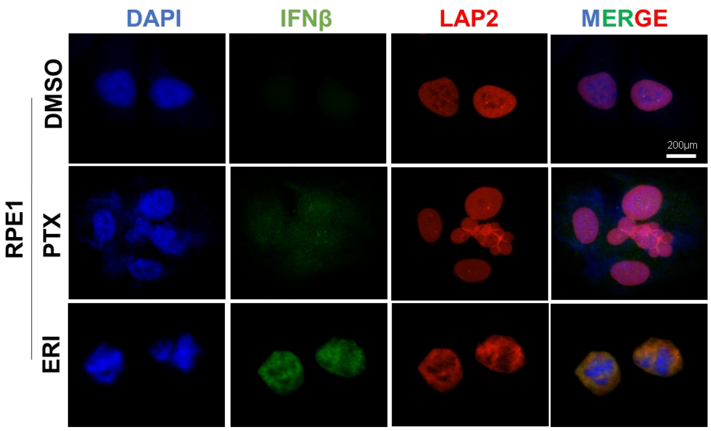

# Supplementary Figure

2nd

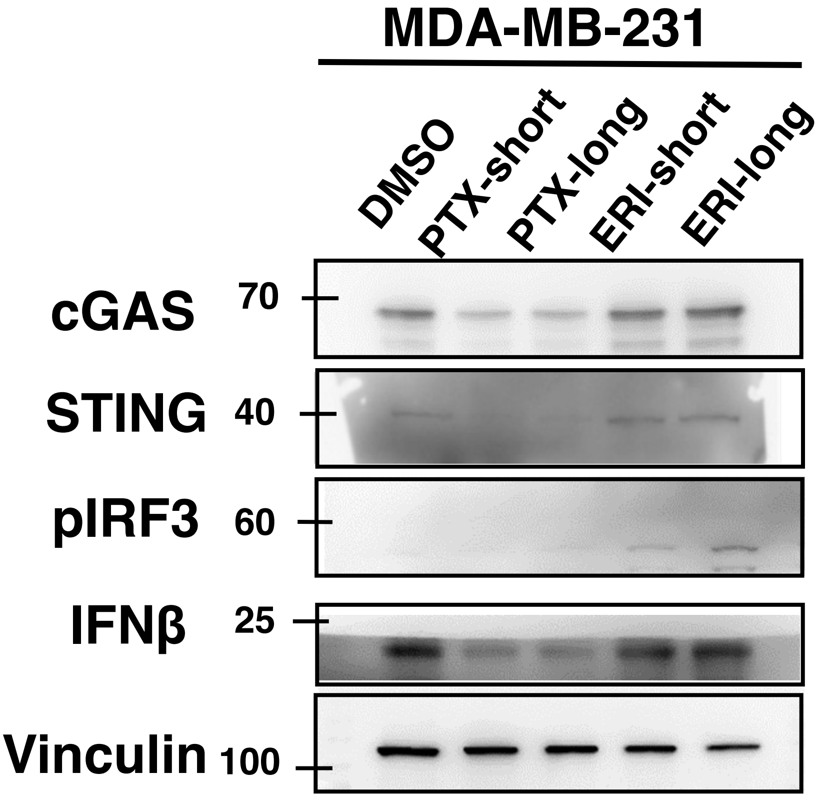


Additional information on Figure 2f

3rd

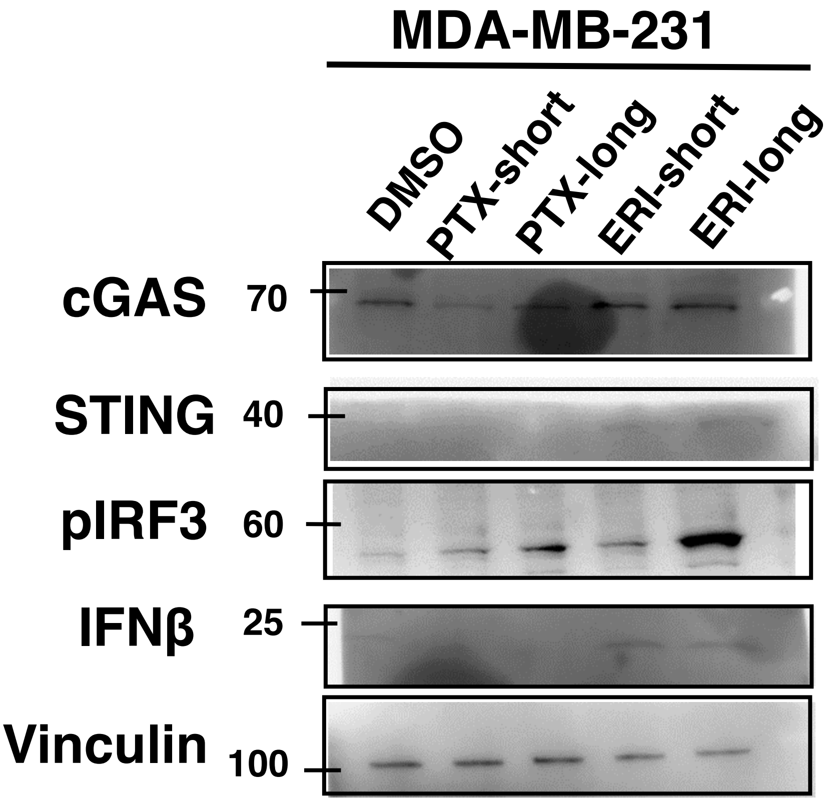


The results of other Western blotting in figure 2f were consistent with the conclusion that cGAS/STING/pIRF3/IFNβ tended to be higher in the ERI long group than in the others.

# Figure 3a

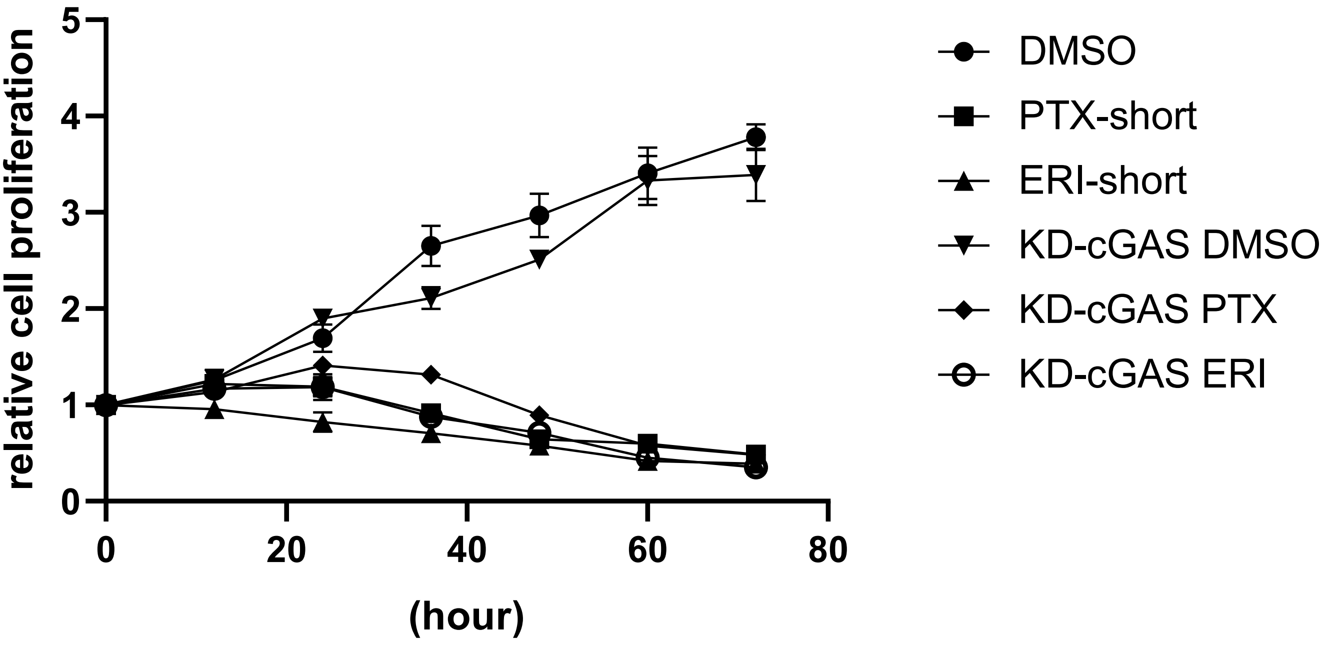


Proliferation at 0 hour was defined as 1, and the relative cell proliferation of above cells was blotted.

$$\text{Relative cell proliferation} = \frac{\text{(Mean OD value of cell A at time X)}}{\text{(Mean OD value of DMSO at 0h)}}$$

※OD: optical density

We would like to change Figure 3a to this graph.

# Supplementary Figure

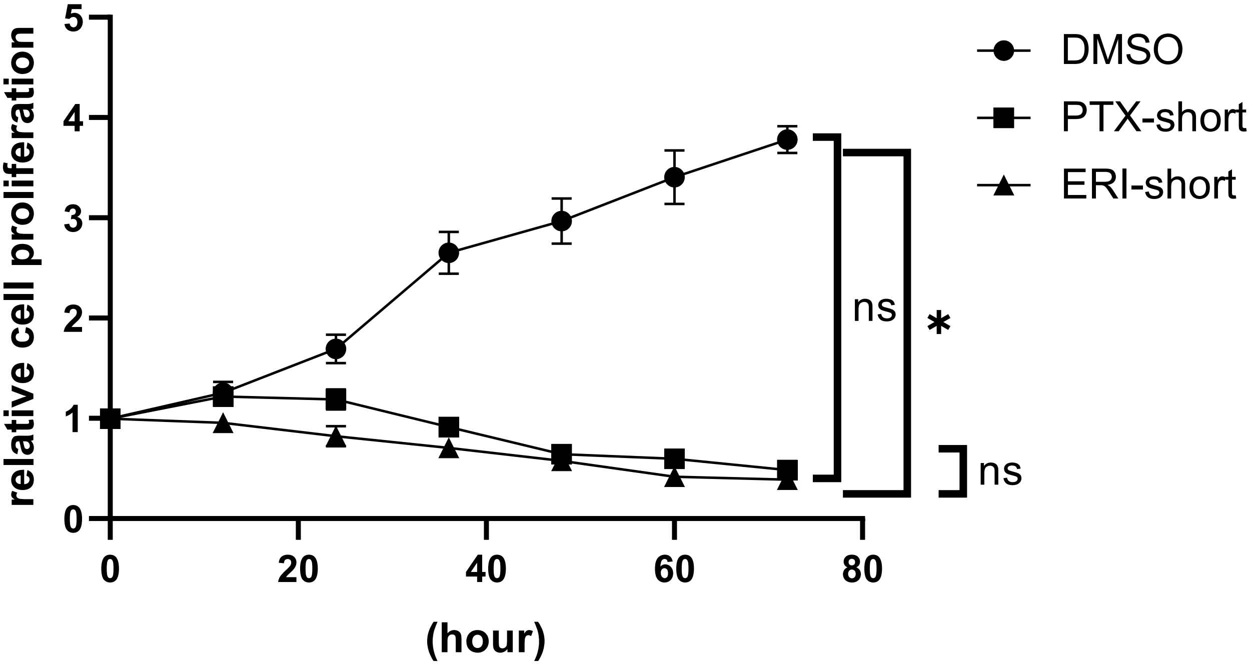


Statistical differences were evaluated by using one-way anova.

ERI-short significantly inhibited cell proliferation compared to DMSO (* p=0.047).
PTX-short inhibited cell proliferation compared to DMSO, but not significantly (p=0.1142).
There was no significant difference in the inhibition of cell proliferation between PTX-short and ERI-short (p>0.9999).

Additional information on Figure 3a

# Supplementary Figure

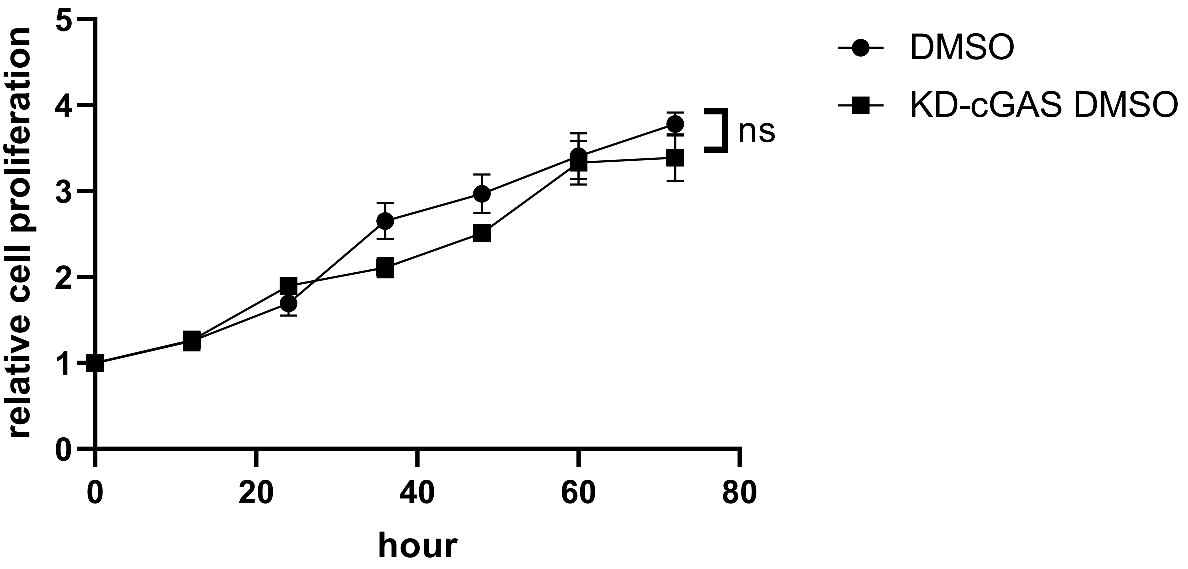


There was no significant difference between DMSO and KD-cGAS-DMSO (p>0.9999).

Additional information on Figure 3a

# Supplementary Figure

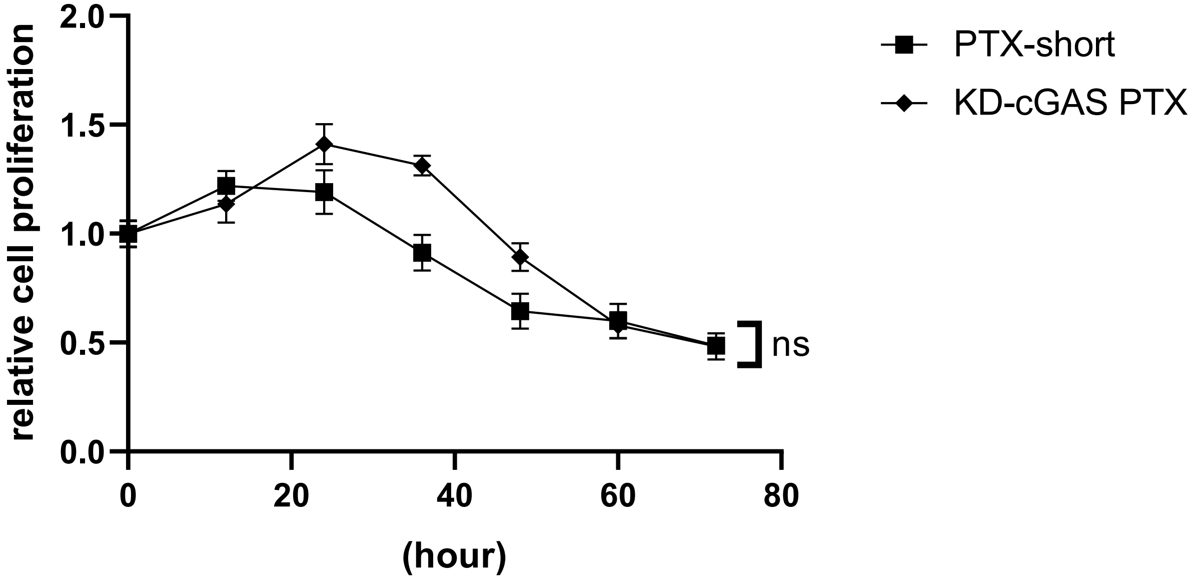


There was no significant difference between PTX-short and KD-cGAS-PTX ($p>0.9999$).

Additional information on Figure 3a

# Supplementary Figure

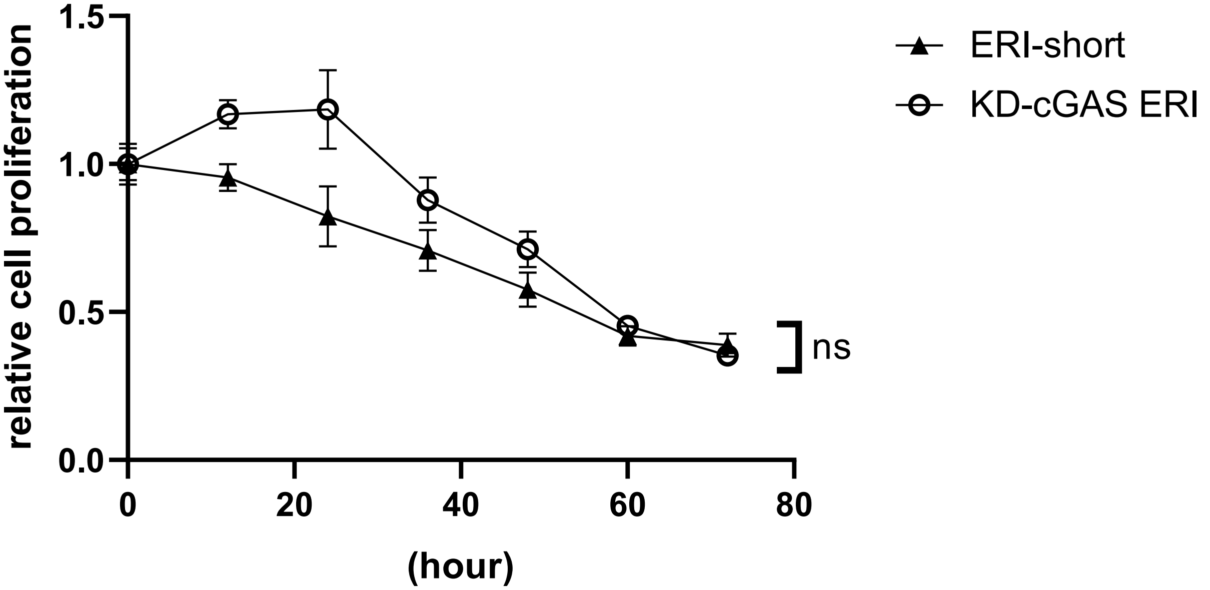


There was no significant difference between ERI-short and KD-cGAS-ERI (p>0.9999).

Additional information on Figure 3a

# Supplementary Figure

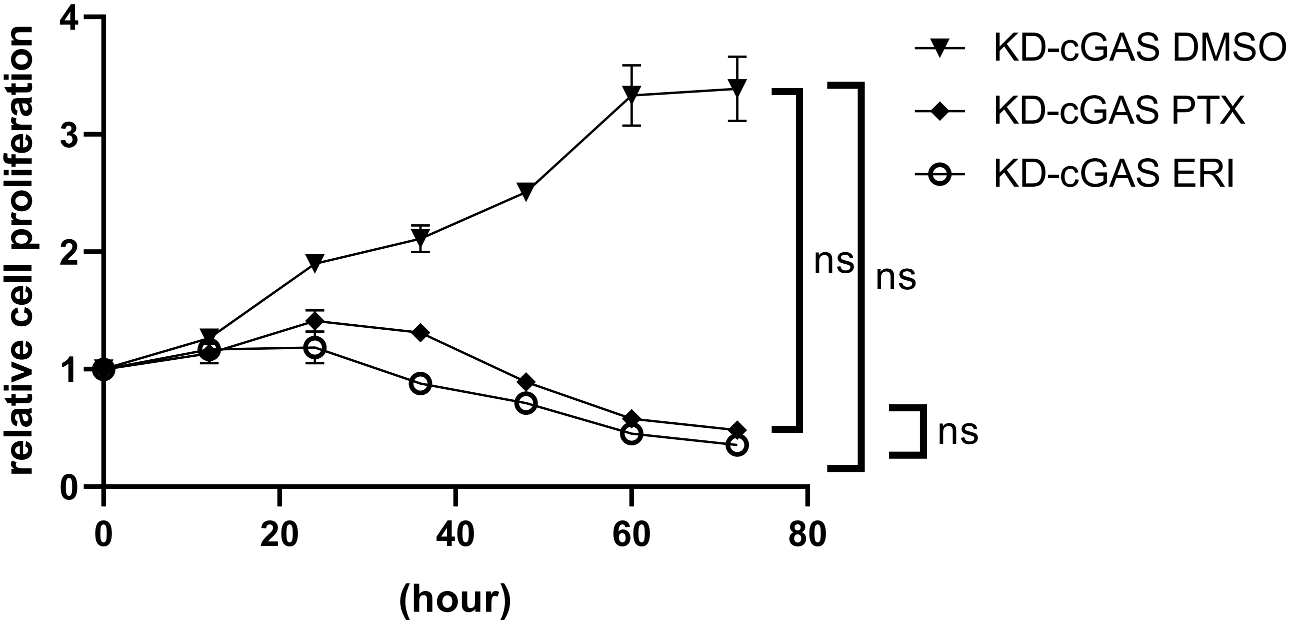


KD-cGAS ERI (p=0.0526) and KD-cGAS PTX (p=0.4404) inhibited cell proliferation compared to KD-cGAS DMSO, but not significantly.
There was no significant difference in the inhibition of cell proliferation between KD-cGAS PTX and KD-cGAS ERI (p>0.9999).

Additional information on Figure 3a

# Figure 3b

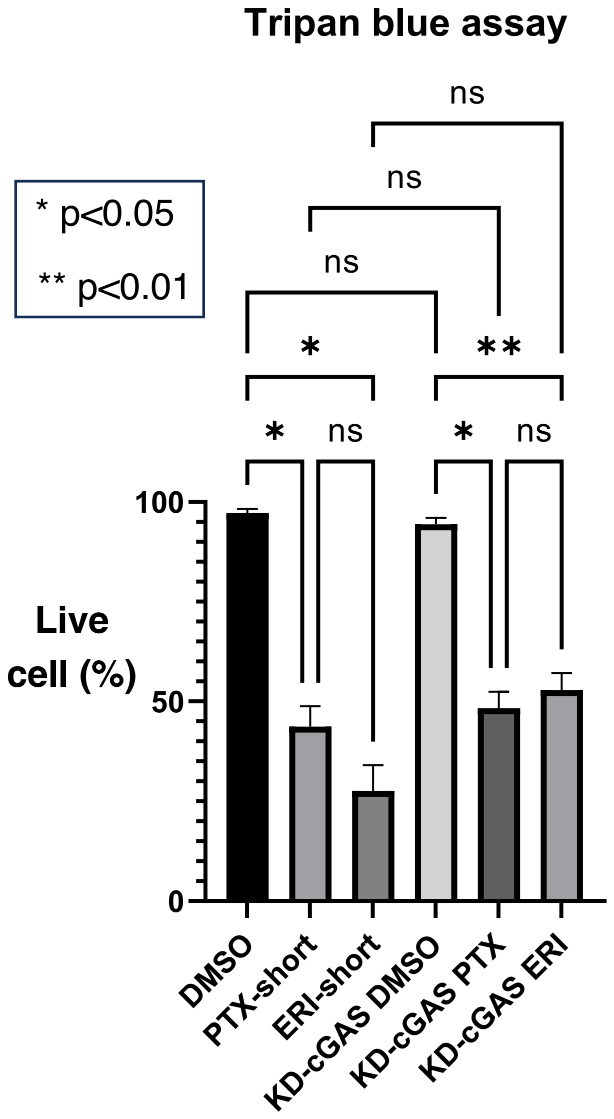


The results of the statistical study are shown.

The percentage of viable cells was statistically compared by unpaired t test.

DMSO and KD-cGAS DMSO were not significantly different in the percentage of viable cells (p=0.3967).

PTX and KD-cGAS PTX were not significantly different in the percentage of viable cells (p=0.5926).

KD-cGAS ERI tended to have a higher percentage of viable cells than ERI short, but the difference was not significant (p=0.0650).

We would like to change Figure 3b to this graph.

# Figure 3c

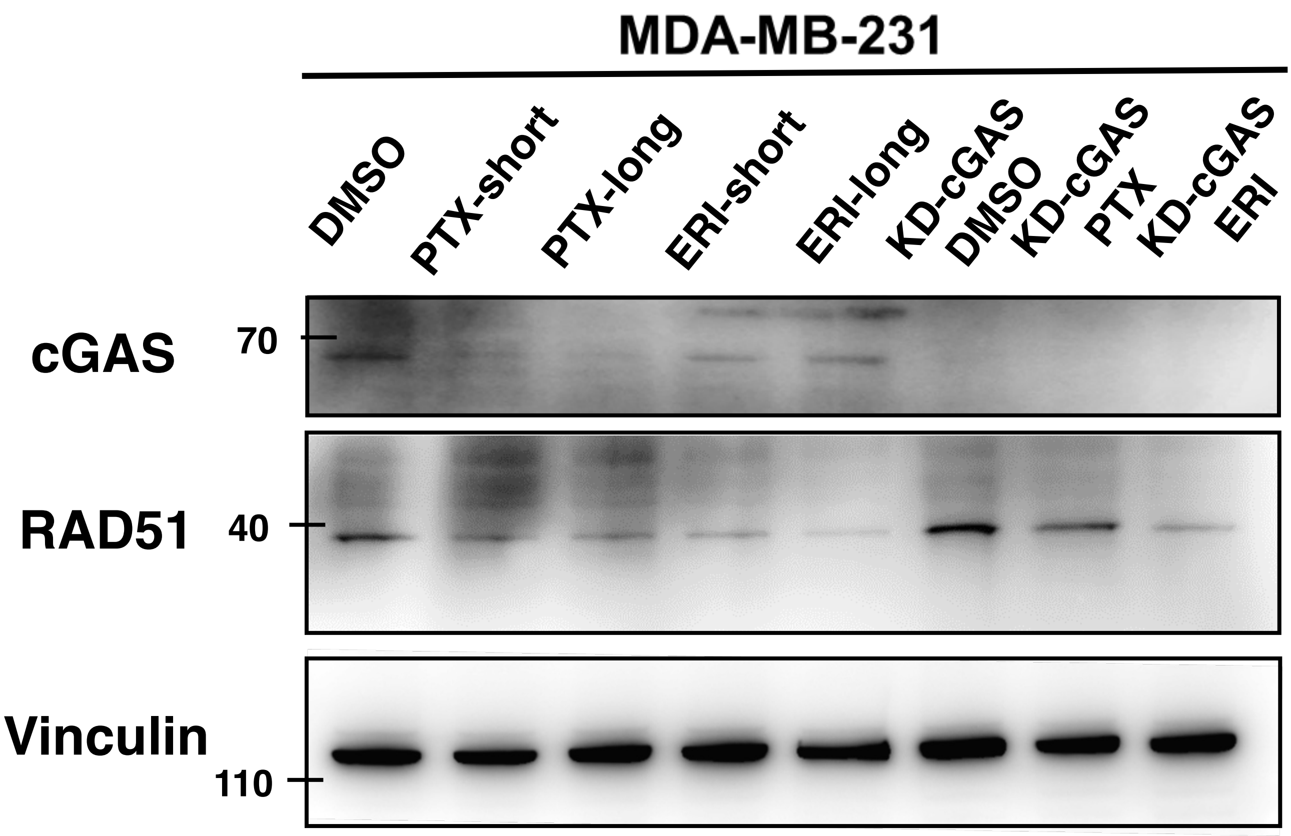


The data shows that cGAS is no longer elevated by ERI or PTX treatment after knockdown of cGAS, and Rad51 is elevated at the same level as in the control in both cases.

We would like to change Figure 3c to this one.

# Figure 3d

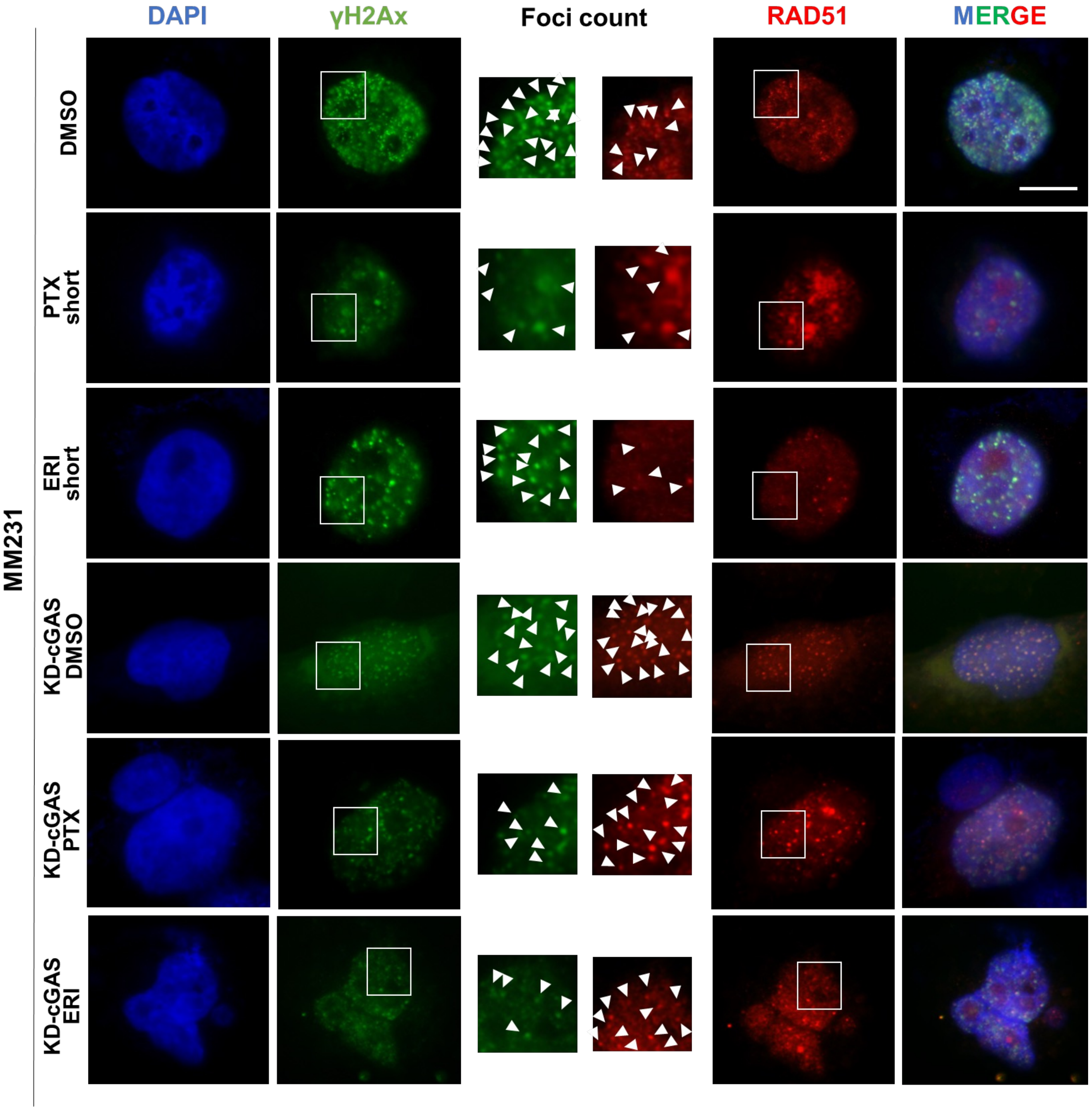


The photos in Figure 3d were not sufficient for evaluation of foci, especially since it was diffusely stained with RAD51.
We would like to replace Figure 3d to this one.

The white bar is 200μm.

White triangles point to foci.

# Supplementary Figure

(×400)

Case 1

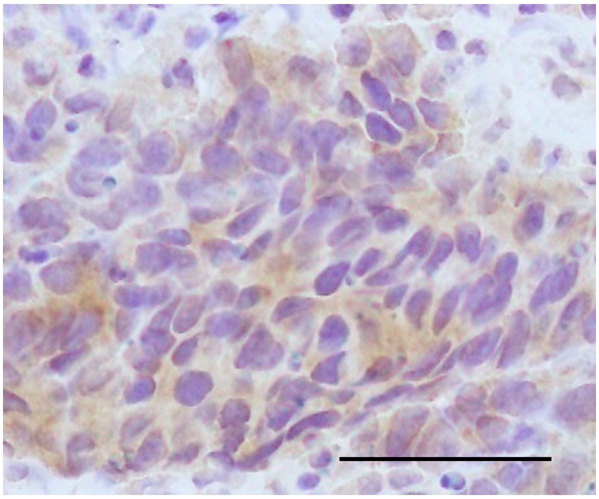

Case 2

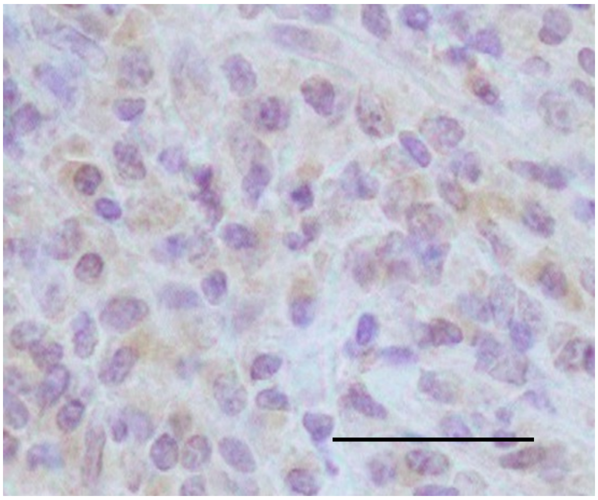

Case 3

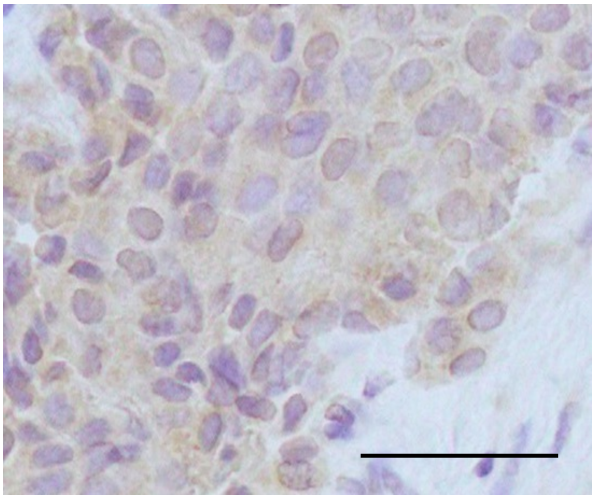

There are three images with IFNβ stained in the cytoplasm rather than the nucleus in IHC.

The black bars are 50μm.
