# Supplementary material for: Eribulin induces micronuclei and enhances the nuclear localization of cGAS in triple-negative breast cancer cells
Source: Sci Rep. 2024 Jun 19;14:14146. doi: 10.1038/s41598-024-64651-y (PMC11187130; doi:10.1038/s41598-024-64651-y)
Supplement: Supplementary file 2 — Supplementary Information 2. [file 41598_2024_64651_MOESM2_ESM.pdf]

Eribulin induces micronuclei and enhances the nuclear localization of cGAS in triple-negative breast cancer cells

Hideyuki Yamada, Mamoru Takada, Dhaval Ghone, Muhan Yu, Takeshi Nagashima, Hiroshi Fujimoto, Junta Sakakibara, Yoshie Hasegawa, Shintaro Takao, Akimitsu Yamada, Kazutaka Narui, Takashi Ishikawa, Aussie Suzuki, Masayuki Otsuka

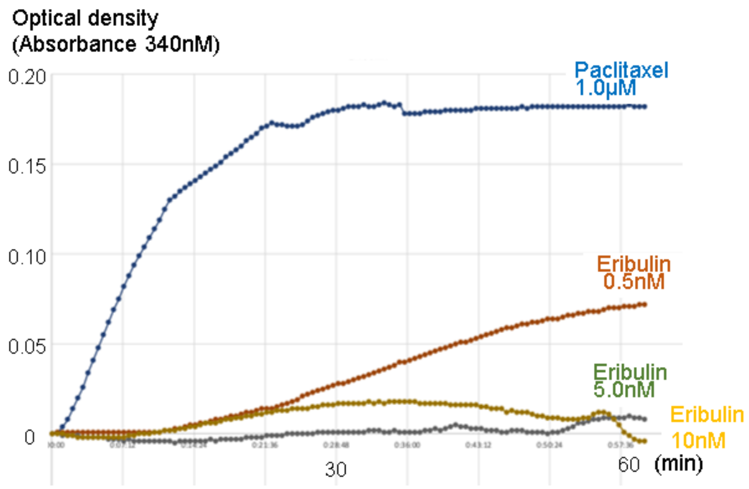

Supplementary Figure 1. The effects of PTX (1.0 μM) and ERI (10 nM, 5 nM, 0.5 nM) on tubulin polymerization were investigated.

Supplementary Figure 1

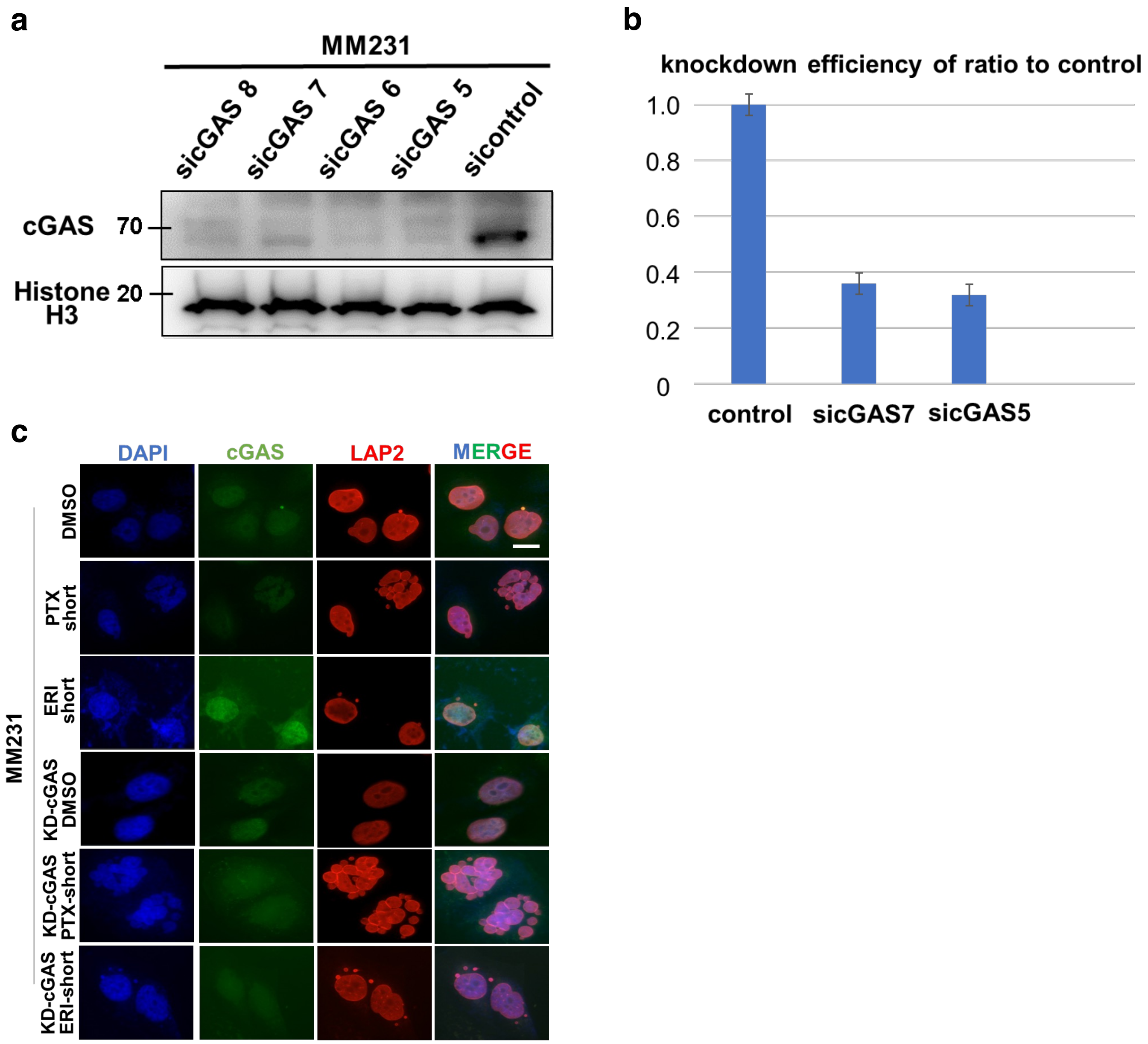

Supplementary Figure 2. The knockdown efficiency were evaluated. (a) Four different siRNAs (siRNA5, siRNA6, siRNA7 and siRNA8) of cGAS were evaluated by WB. Histone H3 was used as loading control. (b) The knockdown efficiency of siRNA5 and siRNA7 compared to control was evaluated by RT-PCR. (c) Immunofluorescence with cGAS was performed to evaluate the effect of KD-cGAS on cell division in MM231.

Supplementary Figure 2 (before revise)

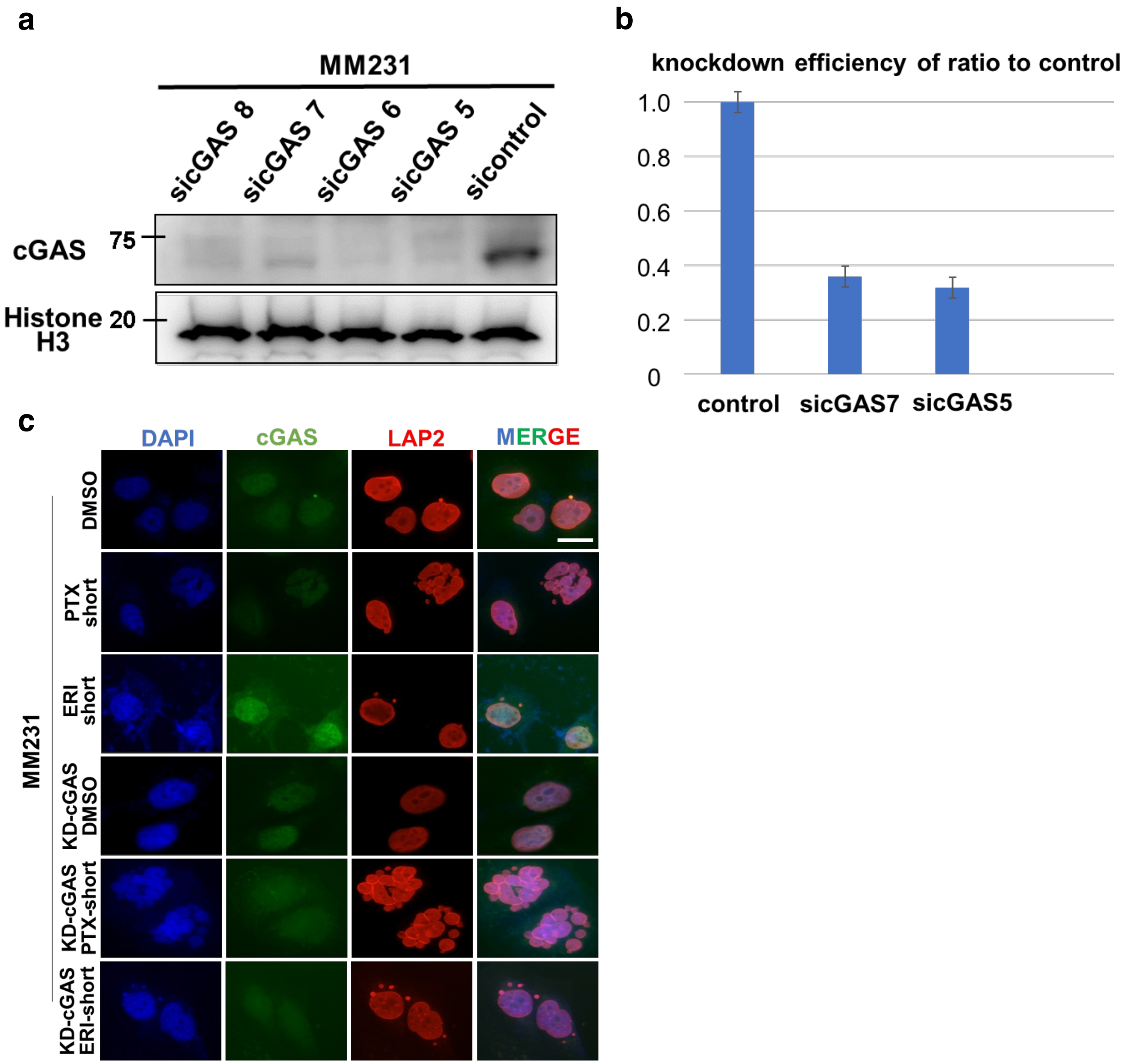

Supplementary Figure 2. The knockdown efficiency were evaluated. (a) Four different siRNAs (siRNA5, siRNA6, siRNA7 and siRNA8) of cGAS were evaluated by WB. Histone H3 was used as loading control. (b) The knockdown efficiency of siRNA5 and siRNA7 compared to control was evaluated by RT-PCR. (c) Immunofluorescence with cGAS was performed to evaluate the effect of KD-cGAS on cell division in MM231. The white line is 10µm.

c) We fixed scale bar.

Supplementary Figure 2 (after revise)

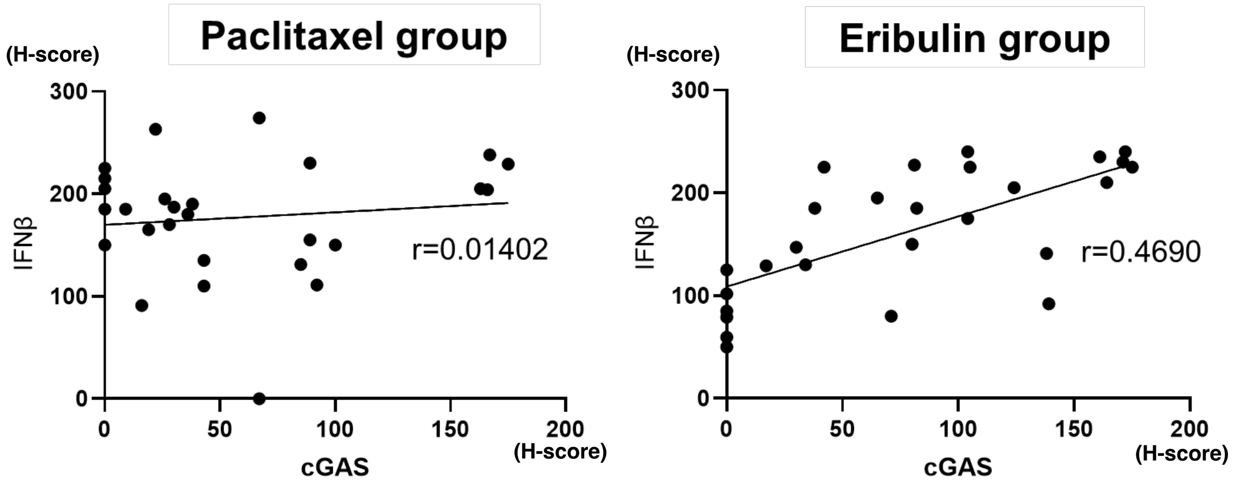

Supplementary Figure 3. The H-score of cGAS and IFNβ were plotted and their correlations were evaluated in both PTX and ERI groups.

Supplementary Figure 3

Agents	cGAS/ IFNβ expression	pCR (n)	non pCR (n)	p value (high/high vs the others)
Paclitaxel	low/ low	1	6	0.5909
	high/ low	2	2	
	low/ high	3	8	
	high/ high	2	3	
Eribulin	low/ low	0	10	0.0005
	high/ low	0	4	
	low/ high	0	3	
	high/ high	5	5	

Supplementary Table1. We evaluated the correlation between the H-score of cGAS/IFNβ and pCR in both PTX and ERI group.

Supplementary Table 1

Original file of repeated experiments

- The values of molecular weight were added to the images of the membrane.
- We used some products with different gel concentrations. Therefore, some of the molecular weights appear different, but we have confirmed that they are in the correct position in that gel.
- Based on reviwer's suggestion, the images of the Figure was revised from the original images.

We have performed at least triplicate of all experiments to confirm our conclusions.

MM231 1st

cGAS

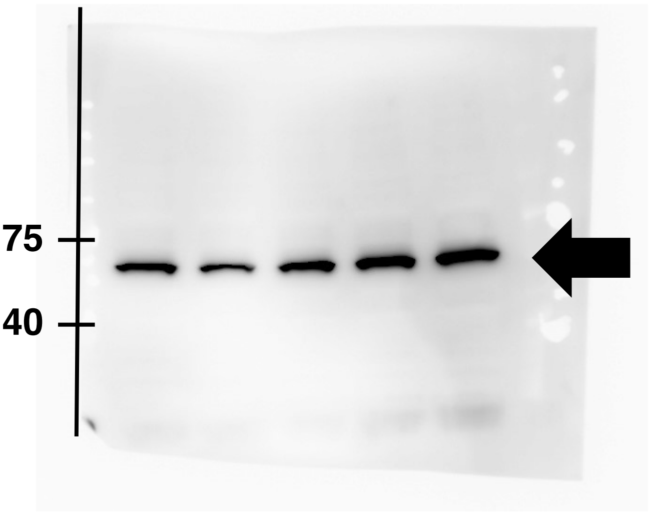

STING

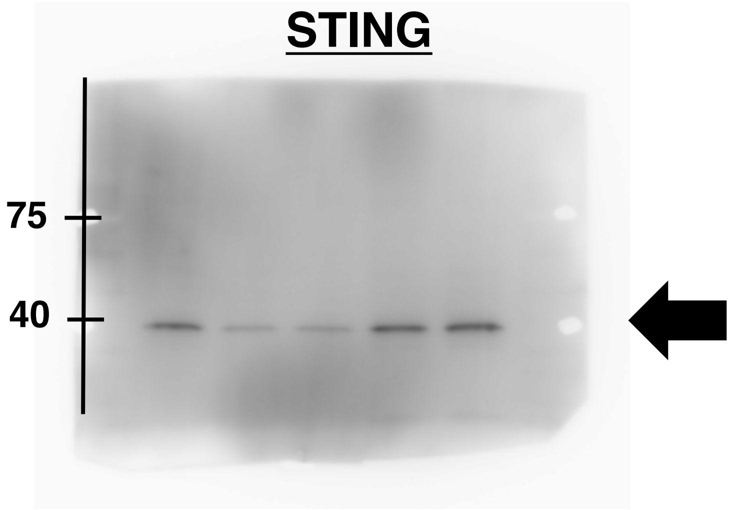

IFNβ

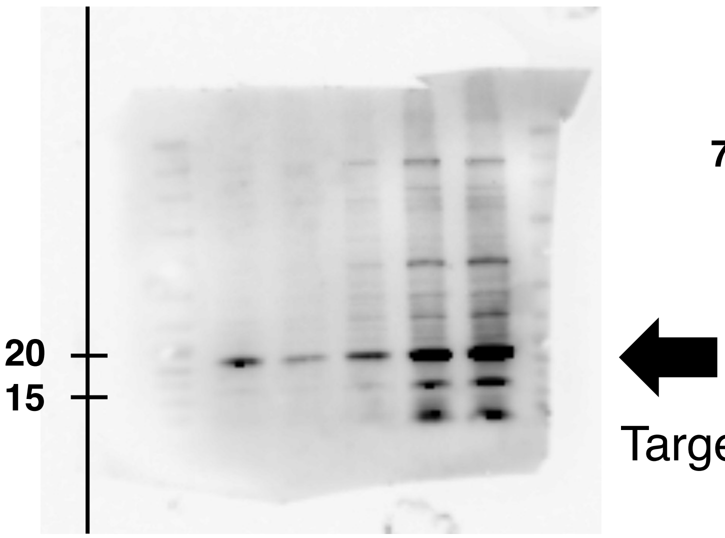

Target band

Vinculin

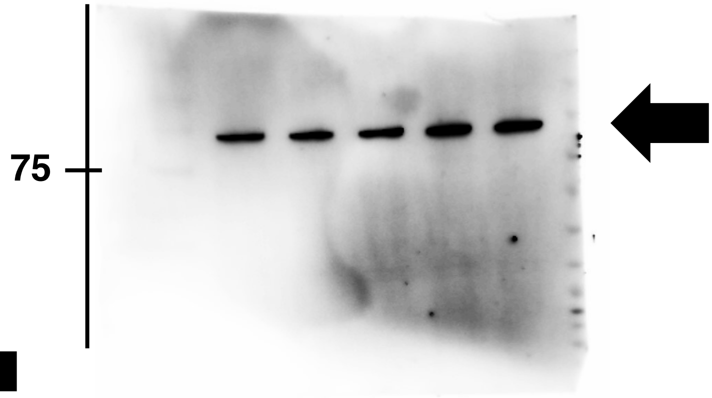

Uncropped Gels and Blots images in MM231 were shown.

Figure 2f

MM231

cGAS

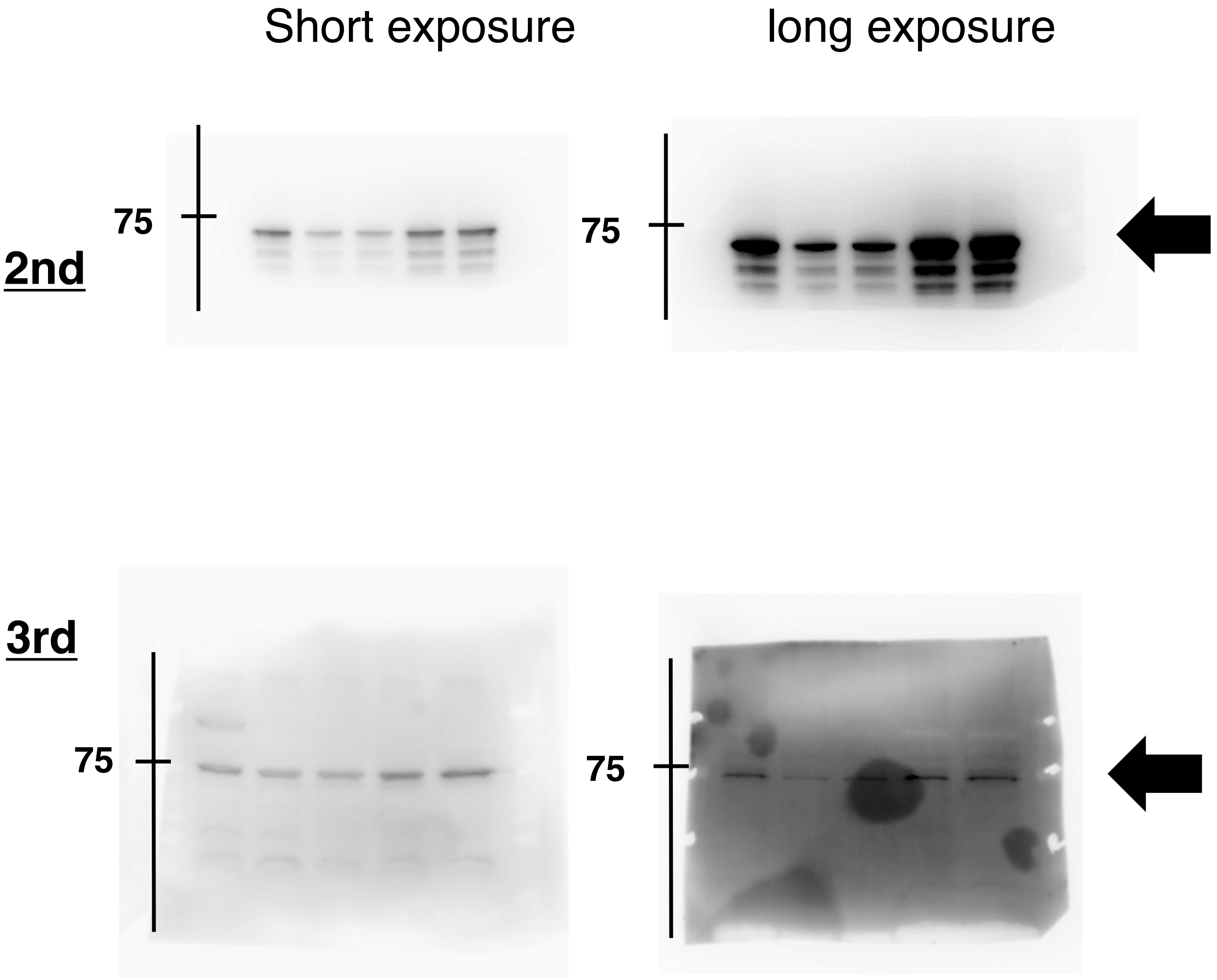

Uncropped Gels and Blots images in MM231 were shown.

Figure 2f

MM231

STING

2nd

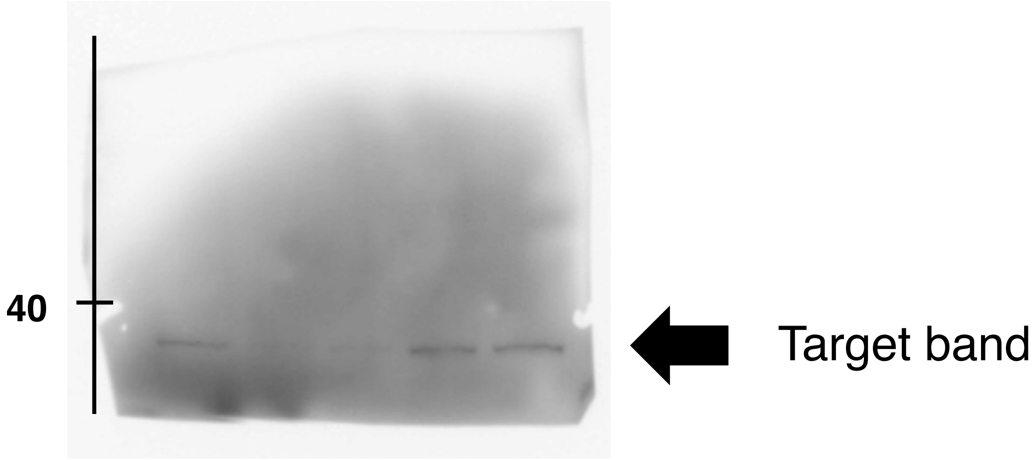

3rd

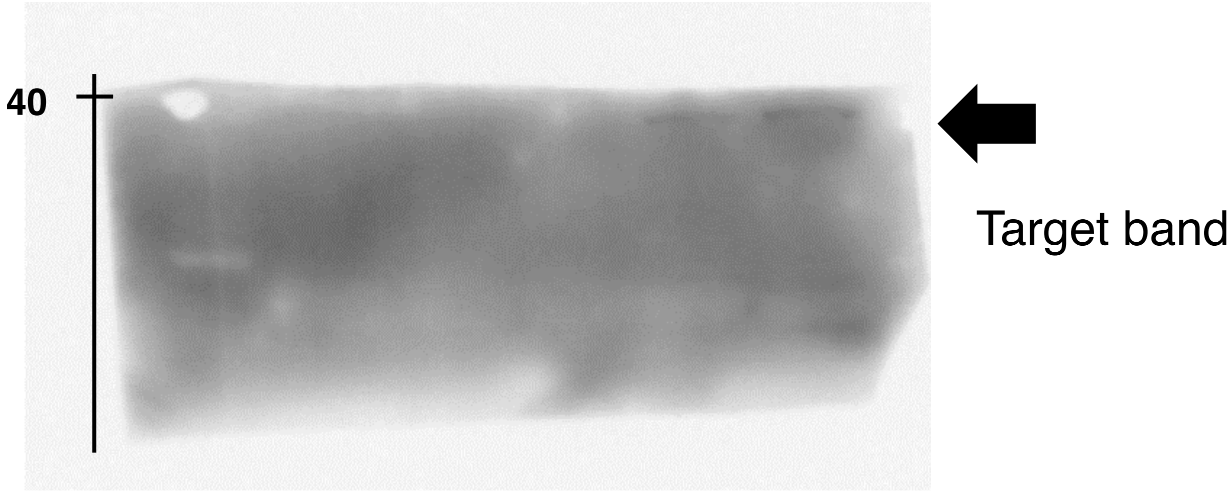

Uncropped Gels and Blots images in MM231 were shown.

Figure 2f

MM231

IFNβ

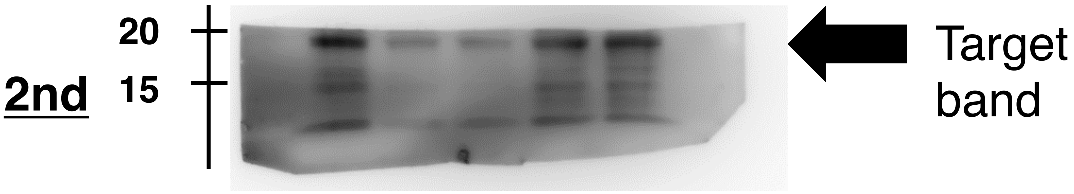

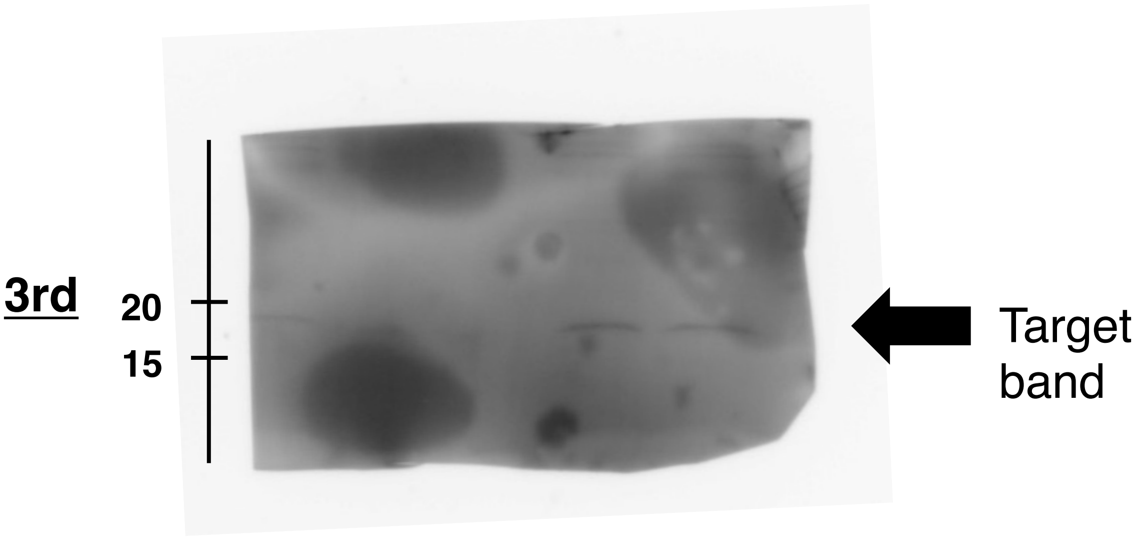

Uncropped Gels and Blots images in MM231 were shown.

Figure 2f

MM231

Vinculin

2nd

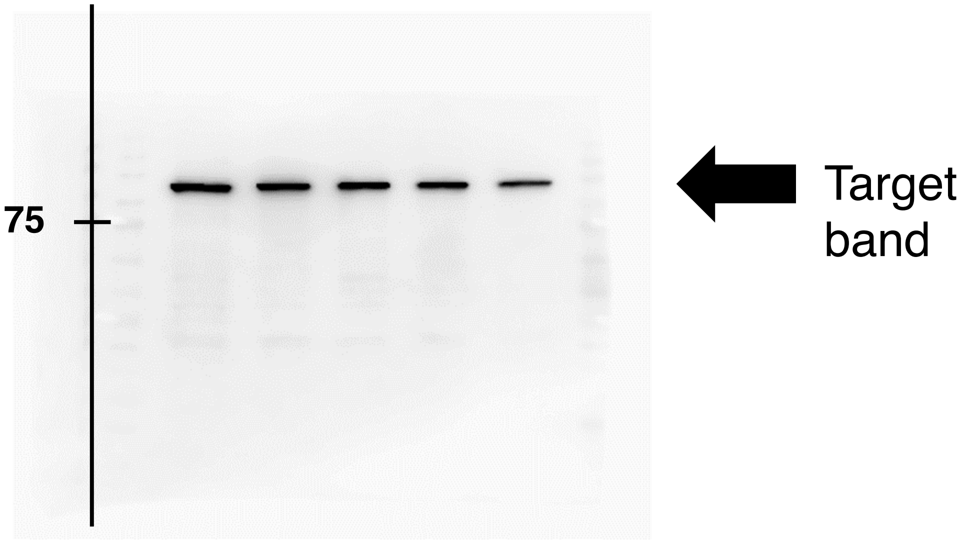

3rd

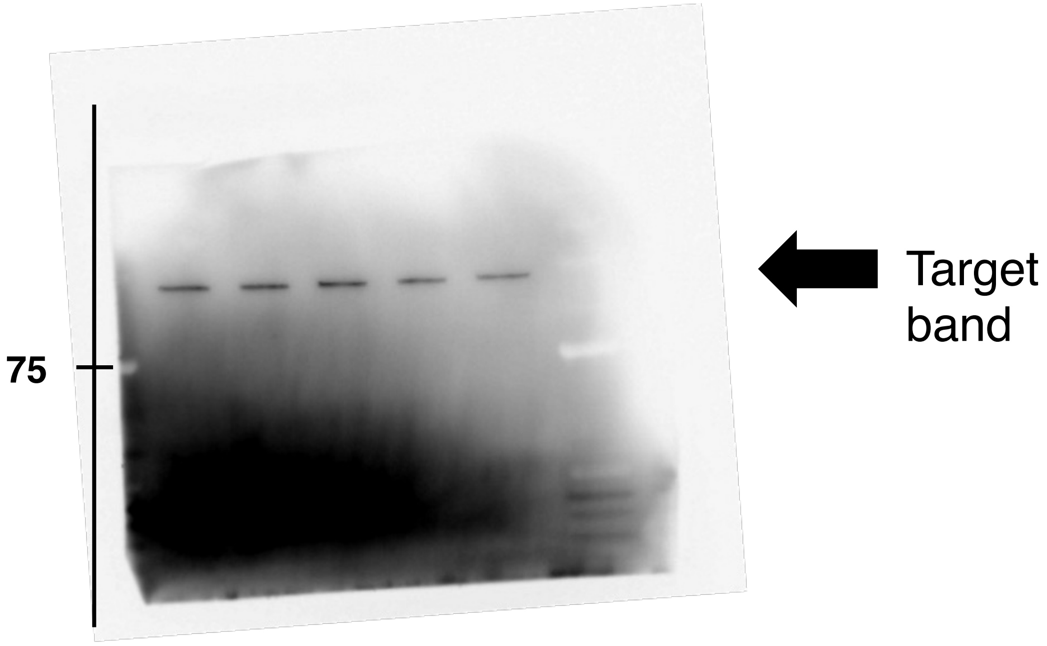

Uncropped Gels and Blots images in MM231 were shown.

Figure 2f

MM231

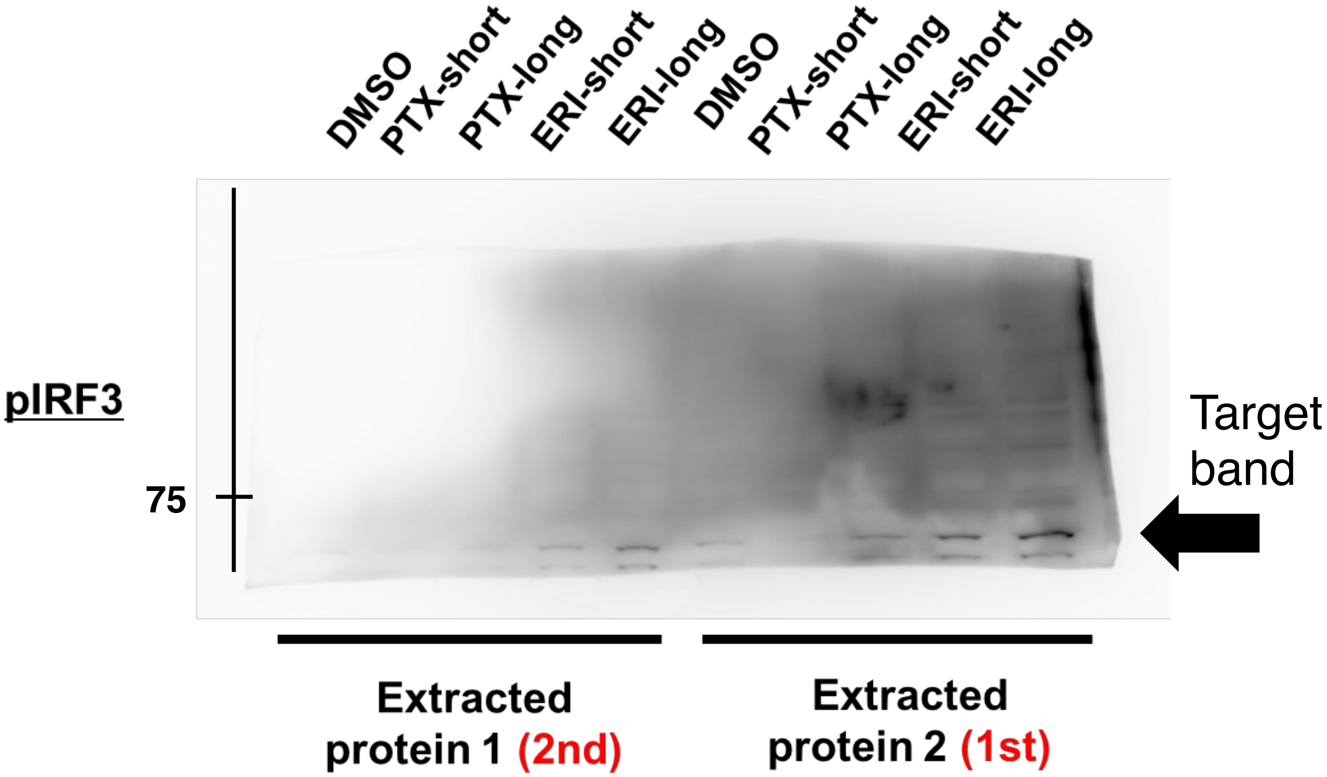

Protein extraction was performed from MM231 cells twice independently and pIRF3 expression was evaluated. The 5 lanes on the left side are ”extracted protein 1” and the 5 lanes on the right side are ”extracted protein 2”. The "extracted protein 2" was used as Figure2f.

Uncropped Gels and Blots images in MM231 were shown.

Figure 2f

MM231

pIRF3

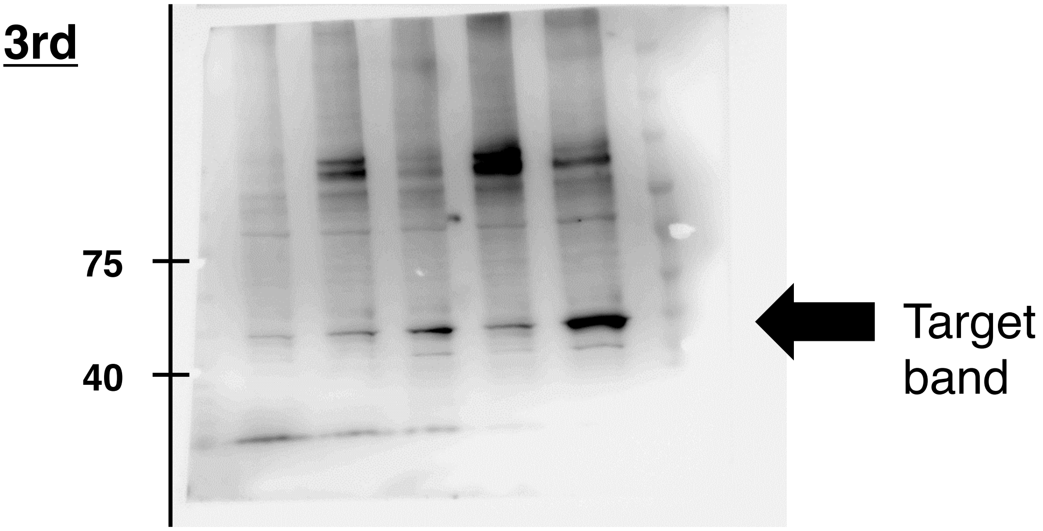

Uncropped Gels and Blots images in MM231 were shown.

Figure 2f

RPE1

cGAS (1st)

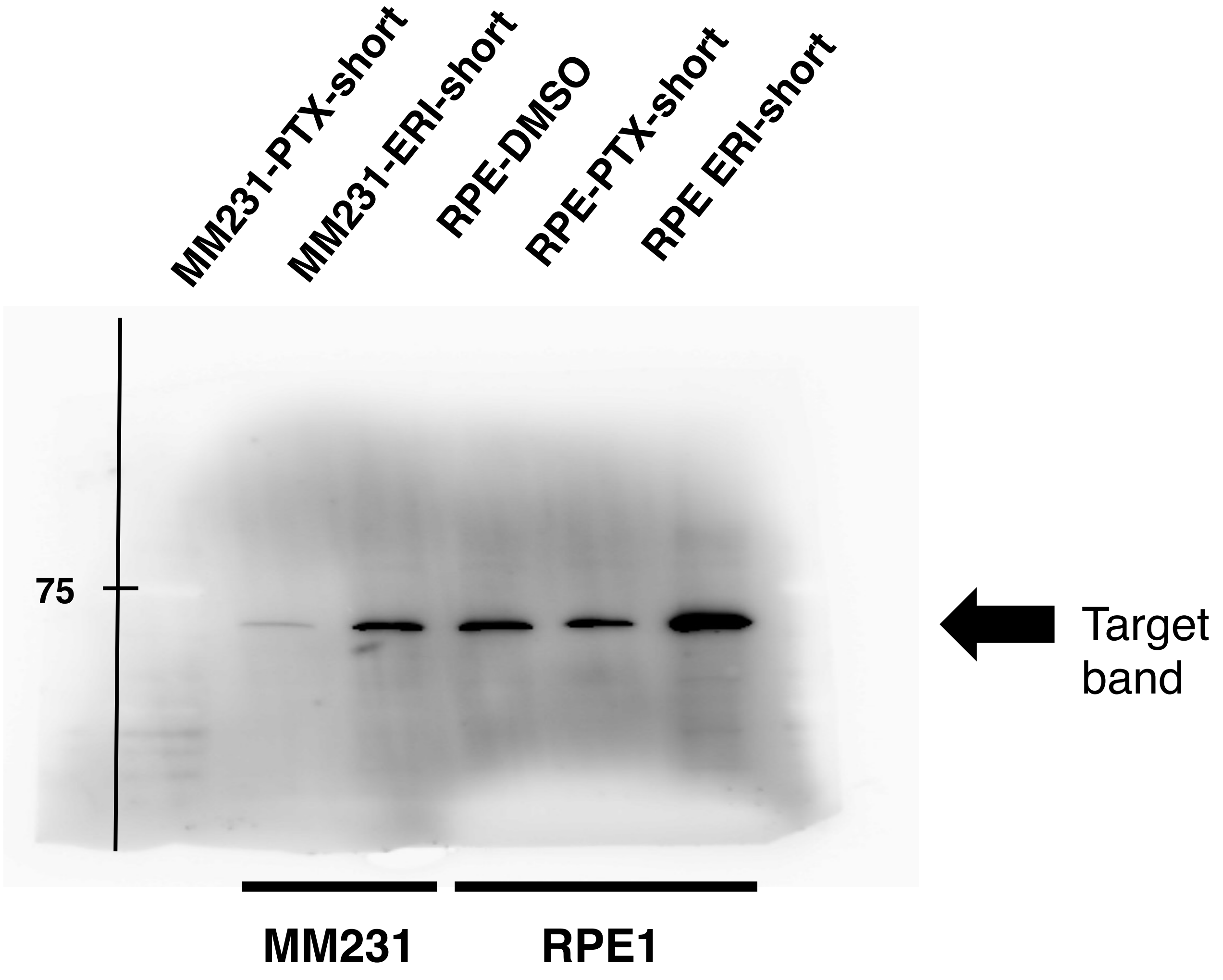

MM231-PTX-short and MM231-ERI-short were also checked whether the bands were detected. Therefore, there are blots of MM231 at the left of the target bands.

Uncropped Gels and Blots images in RPE1 were shown.

Figure 2f

RPE1

cGAS

2nd

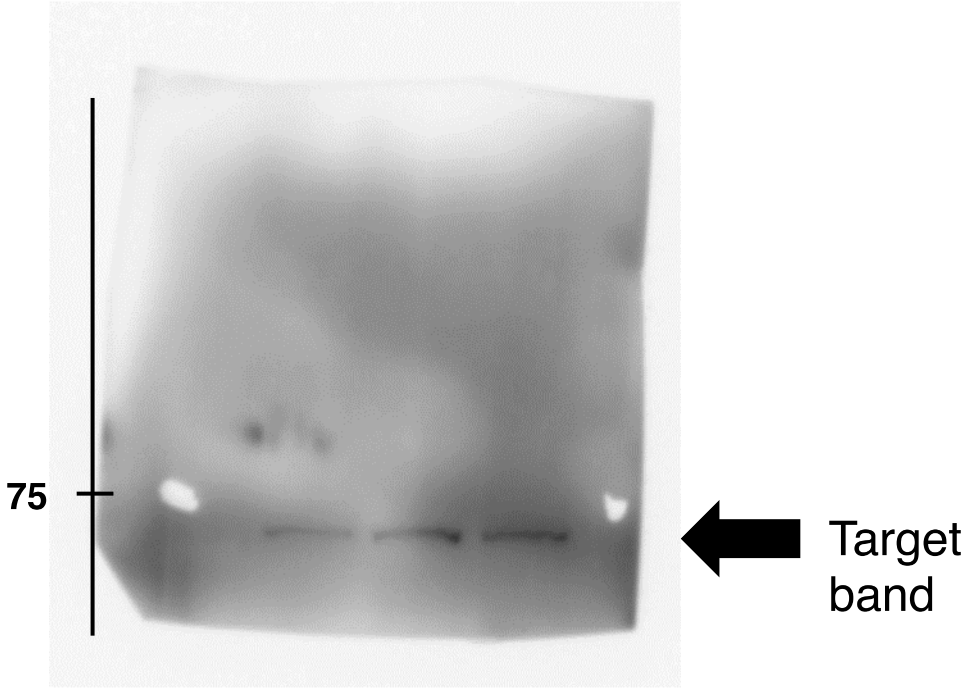

3rd

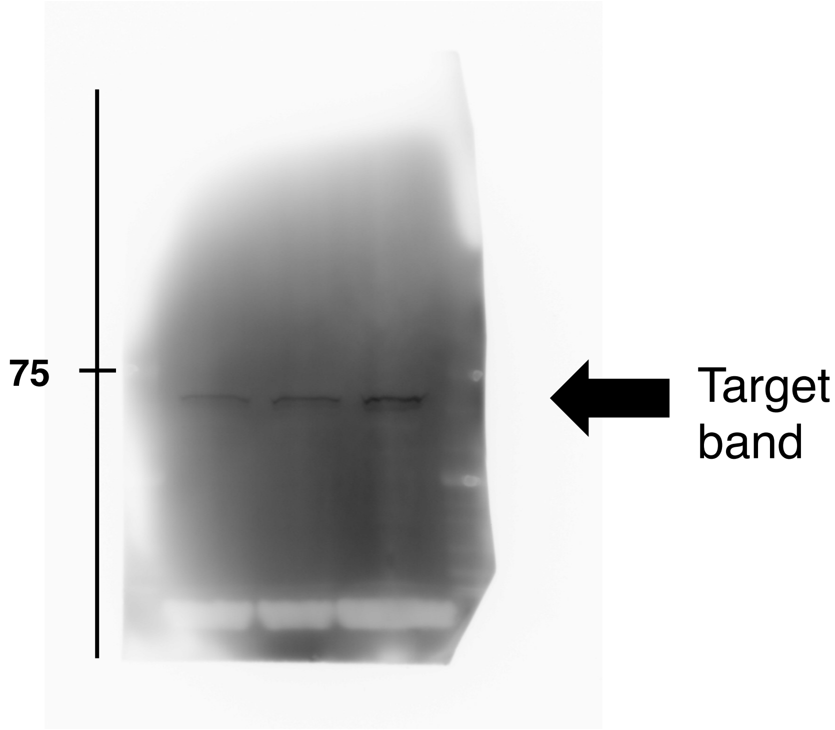

Uncropped Gels and Blots images in RPE1 were shown.

Figure 2f

RPE1

STING (1st)

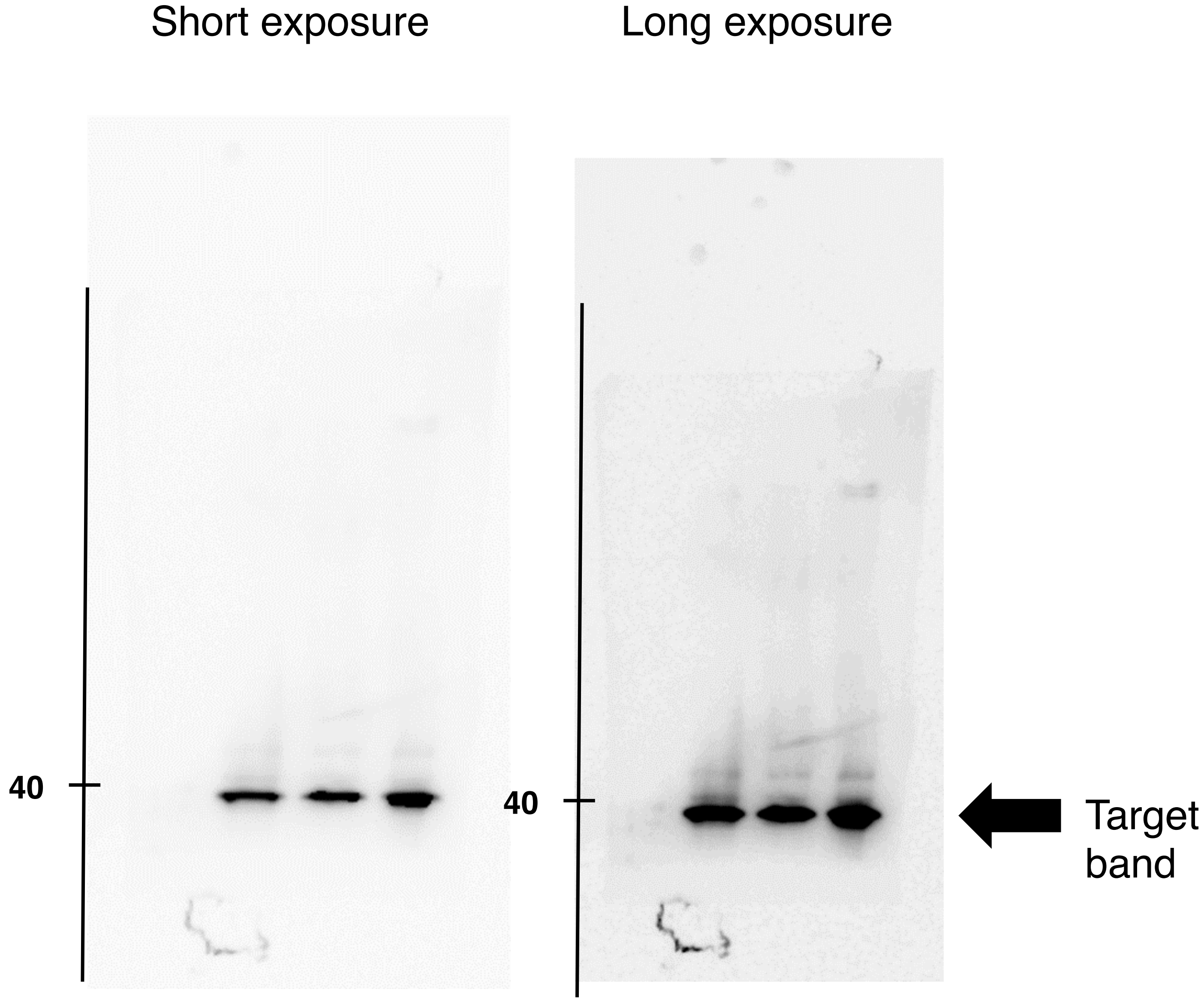

Uncropped Gels and Blots images in RPE1 were shown.

Figure 2f

RPE1

STING

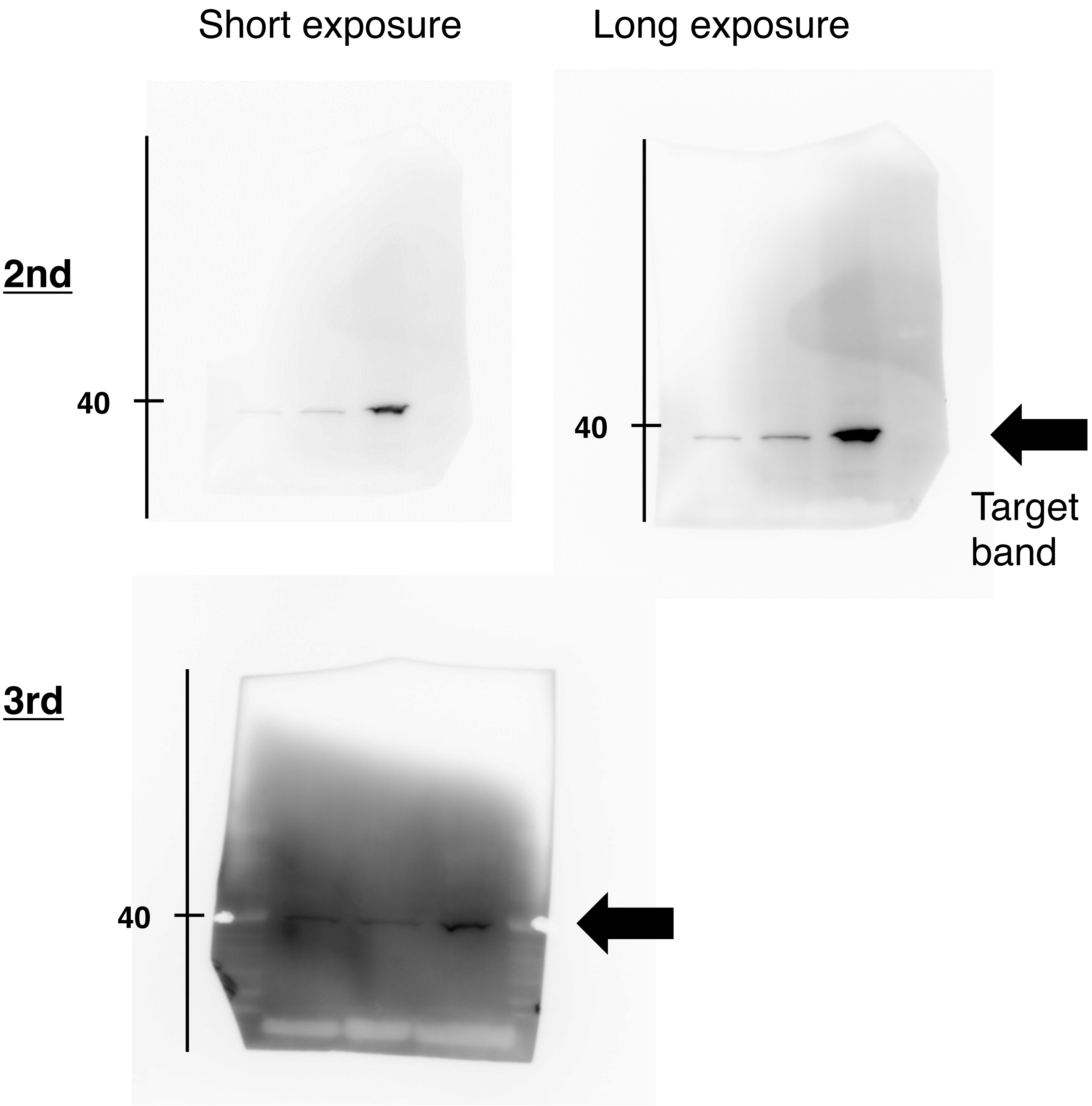

Uncropped Gels and Blots images in RPE1 were shown.

Figure 2f

RPE1

pIRF3

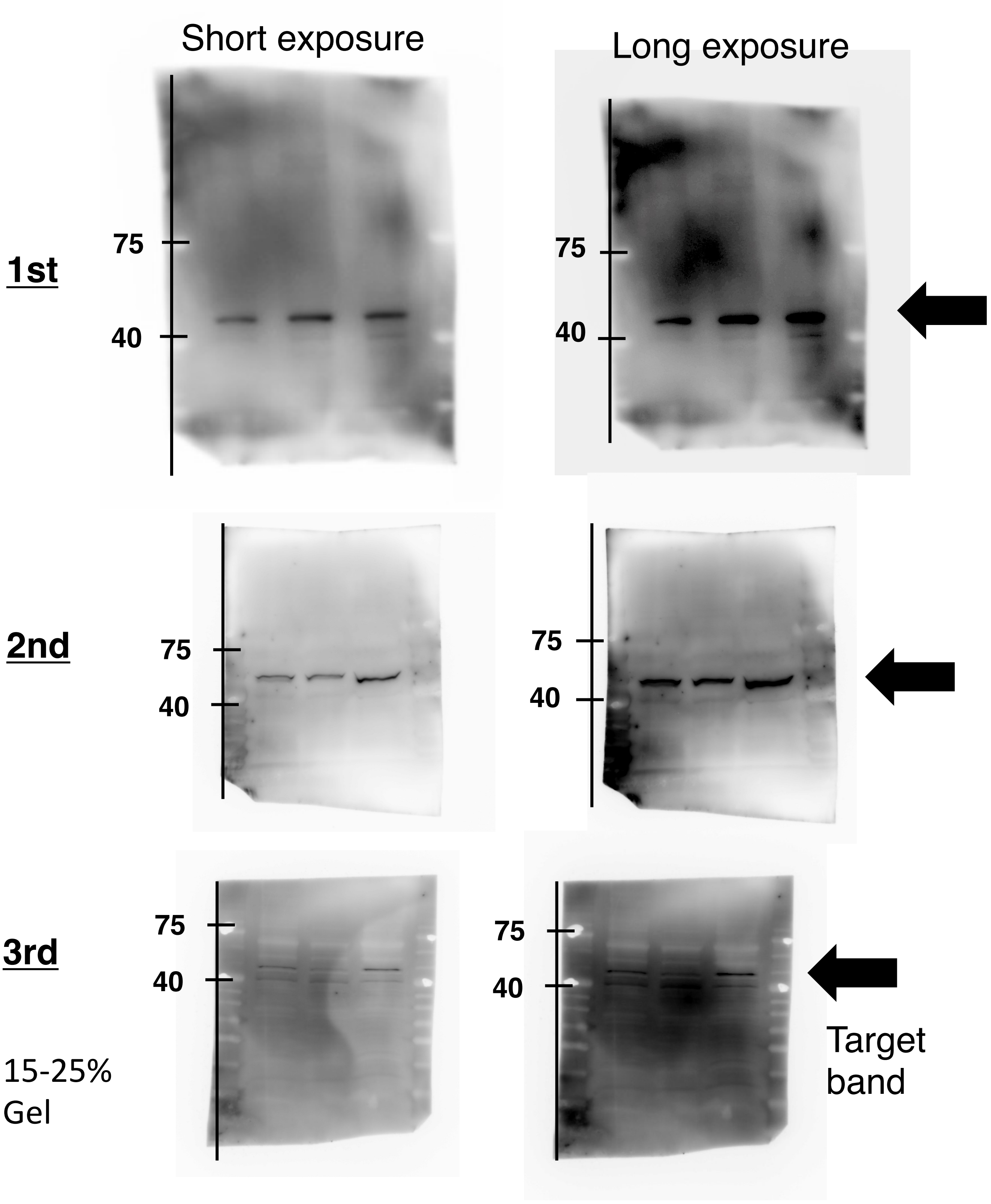

We used a 15-25% gel in the third procedure.
Uncropped Gels and Blots images in RPE1 were shown.

Figure 2f

RPE1

IFNβ (1st)

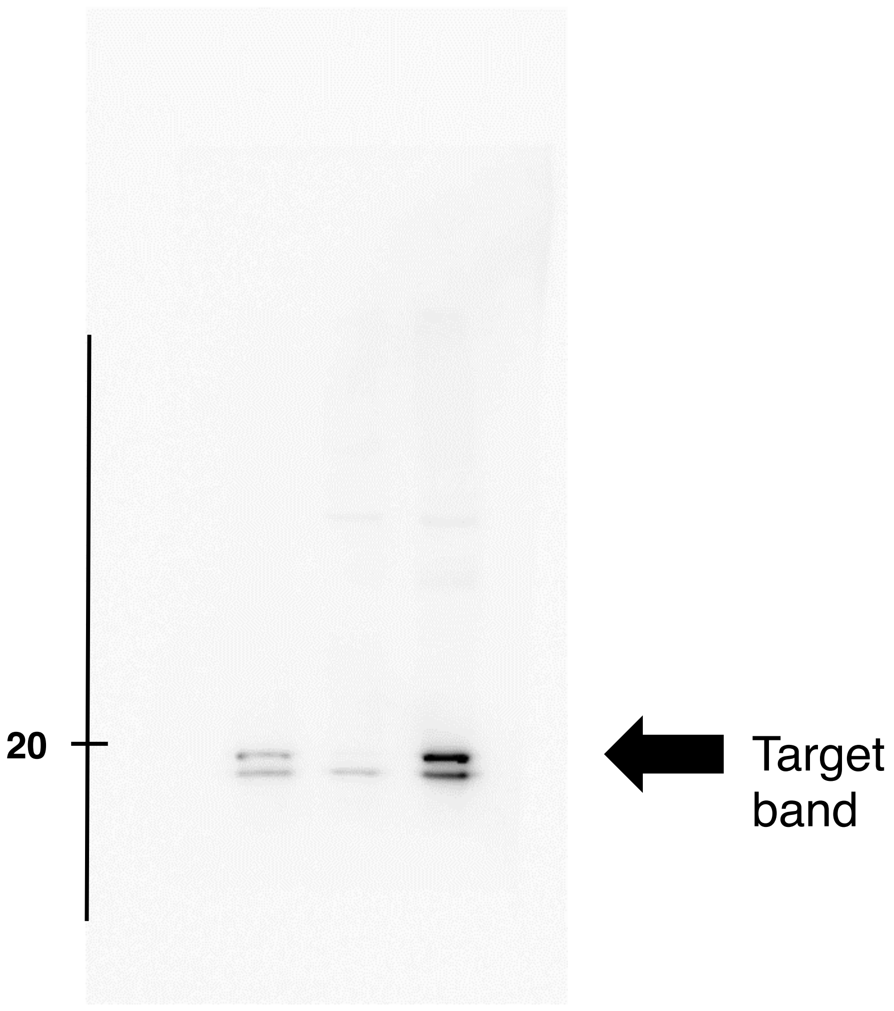

Uncropped Gels and Blots images in RPE1 were shown.

Figure 2f

RPE1

IFNβ

2nd

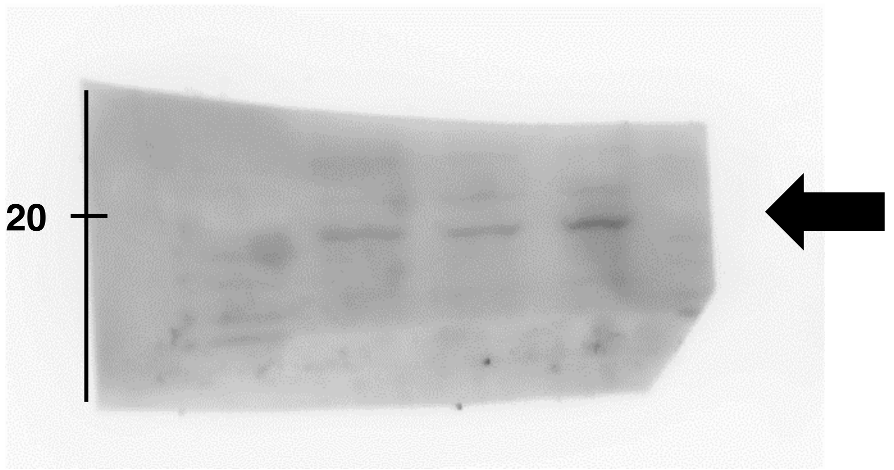

3rd

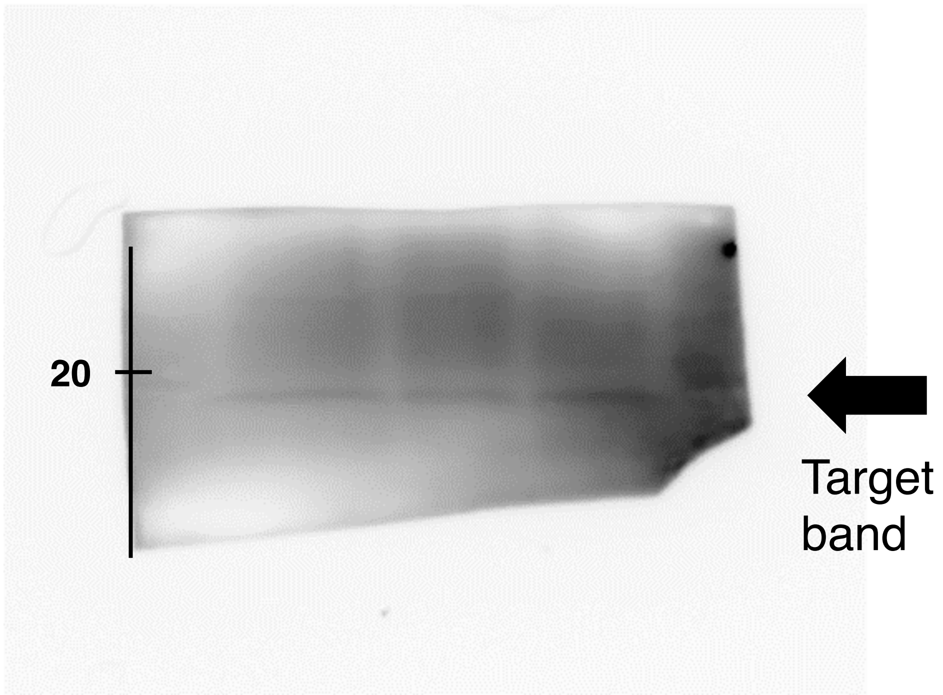

Uncropped Gels and Blots images in RPE1 were shown.

Figure 2f

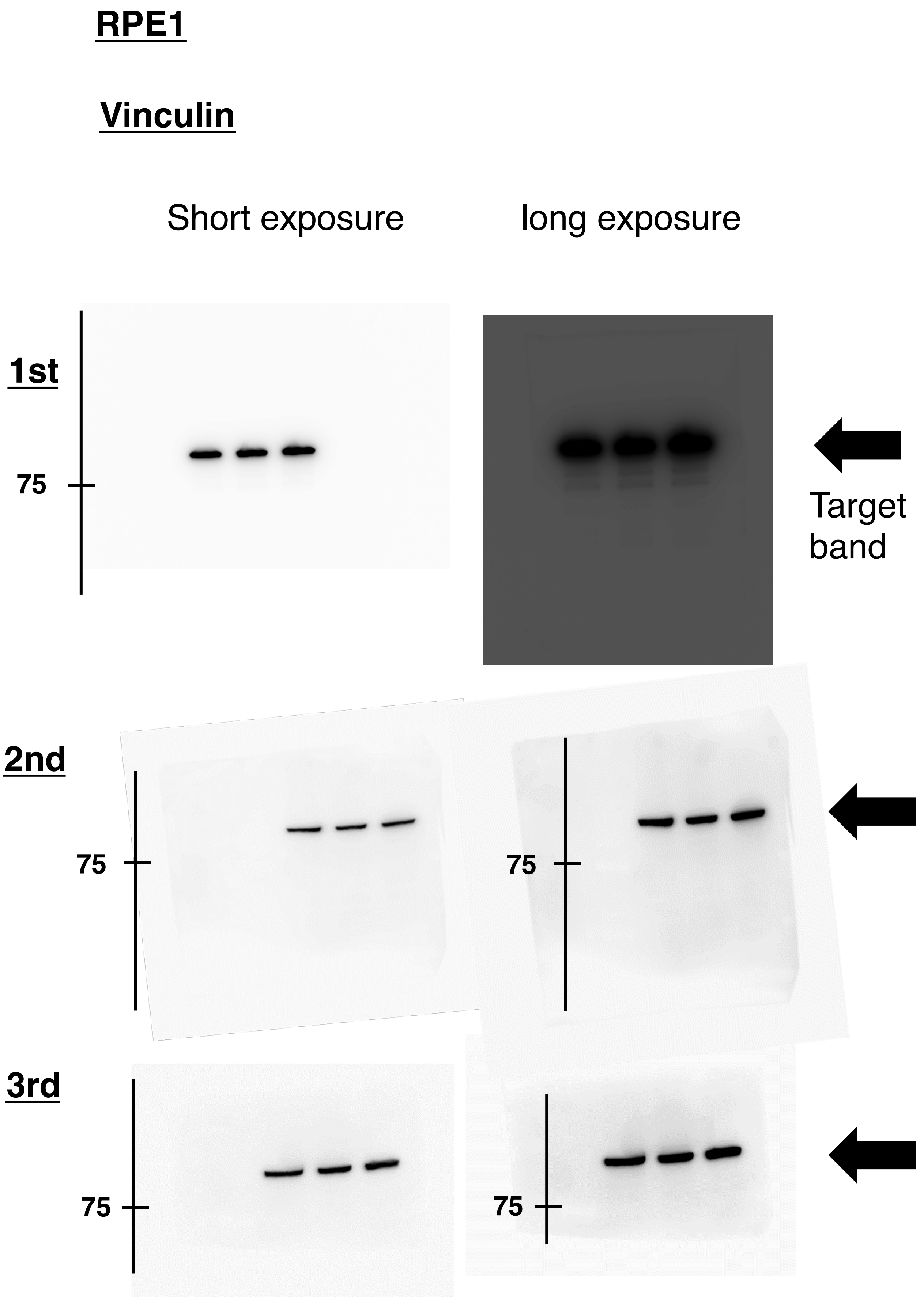

Uncropped Gels and Blots images in RPE1 were shown.

Figure 2f

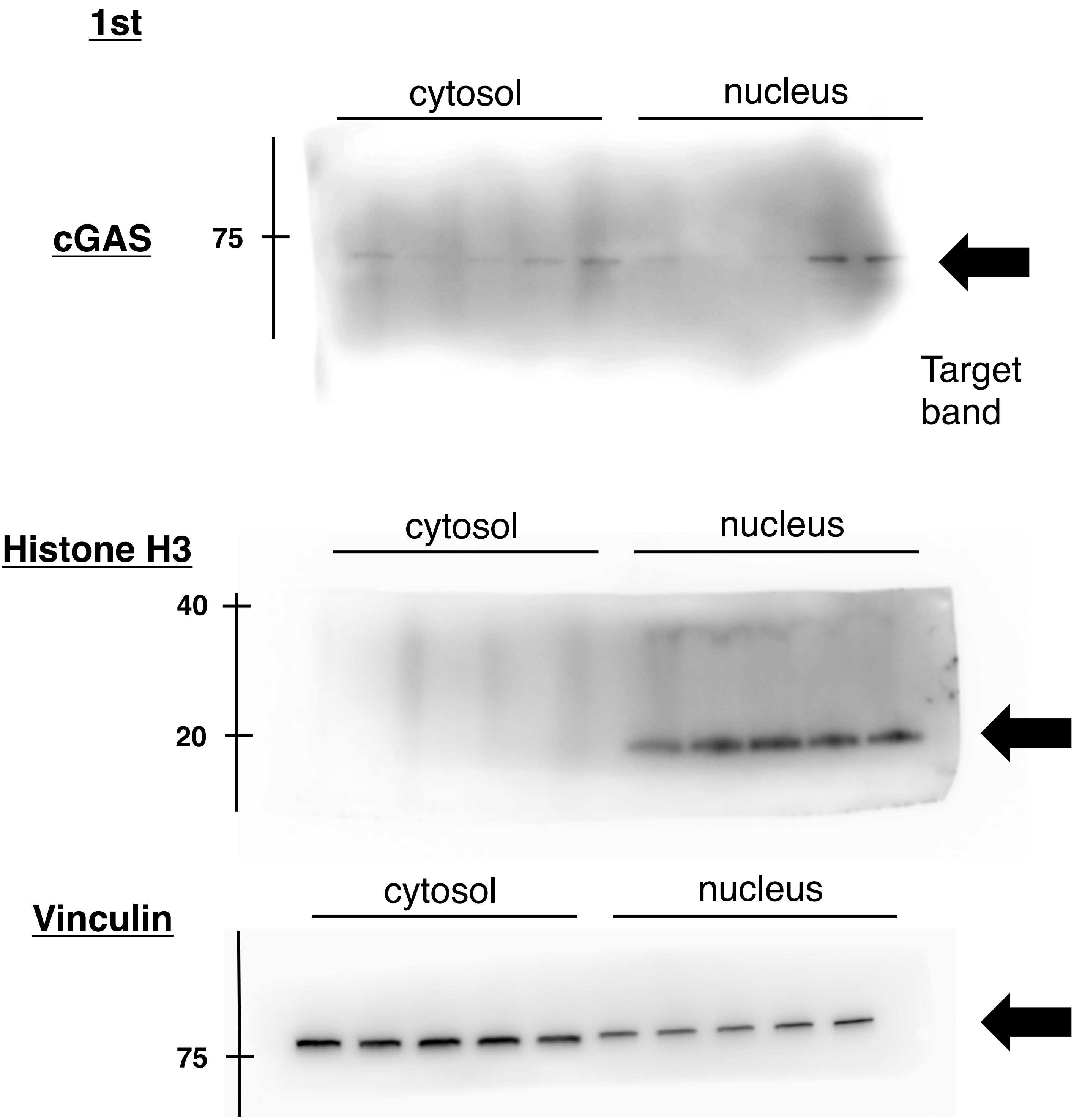

The order of the cytoplasmic and nuclear fractions is reversed in the 2nd and 3rd procedures.

Uncropped Gels and Blots images of Figure 2g were shown.

Figure 2g

cGAS

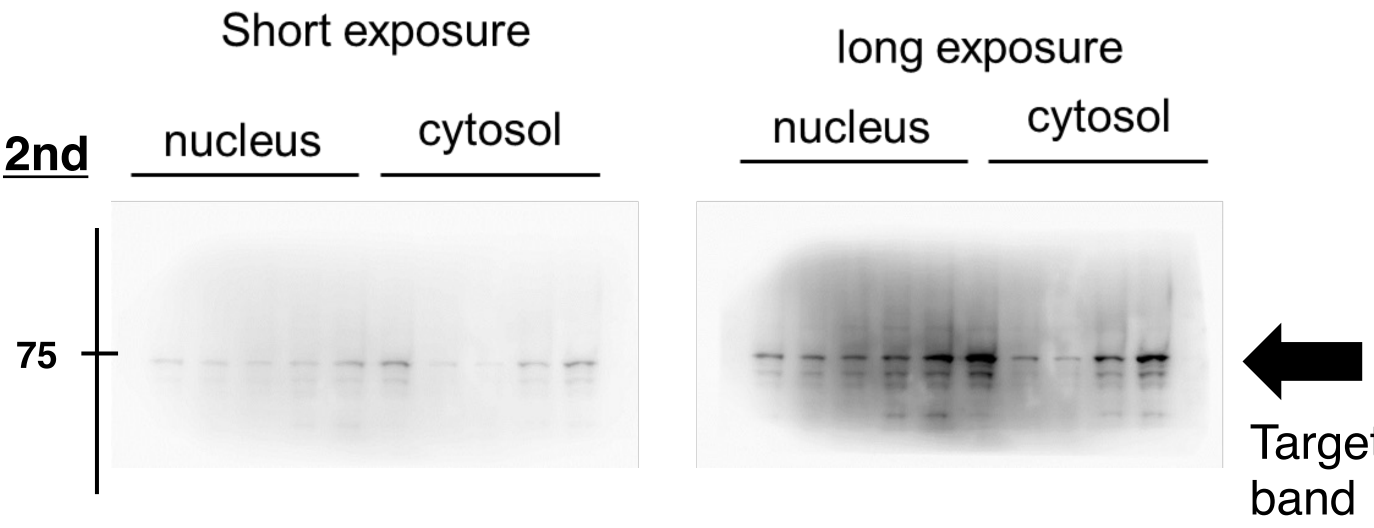

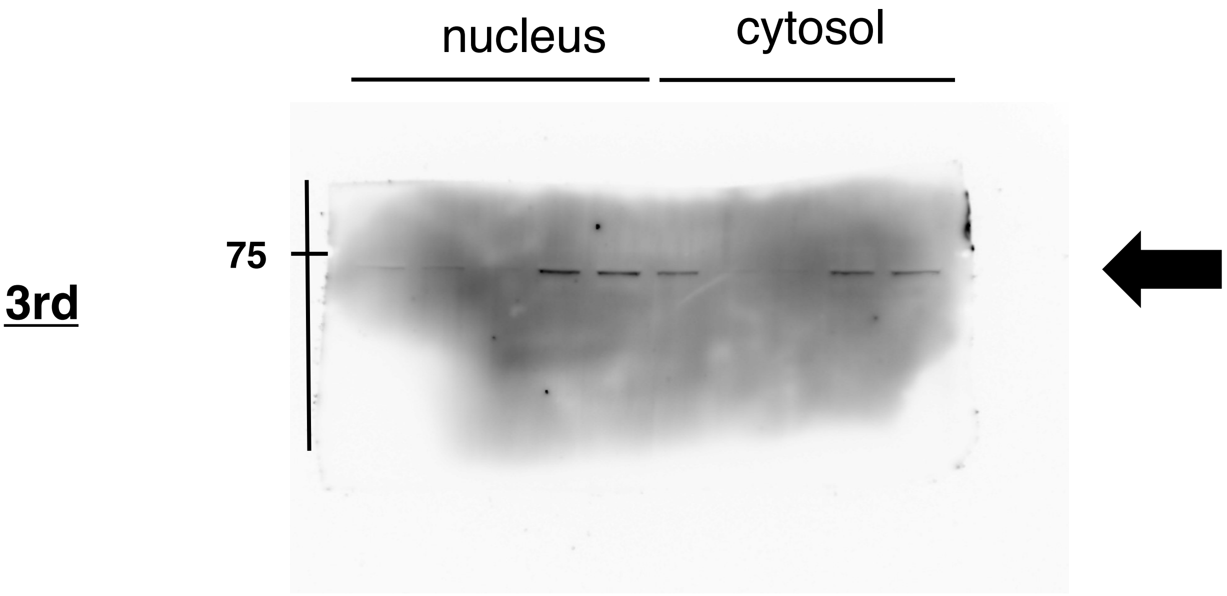

The order of the cytoplasmic and nuclear fractions is reversed in the 2nd and 3rd procedures.

Uncropped Gels and Blots images of Figure 2g were shown.

Figure 2g

Histone H3

2nd

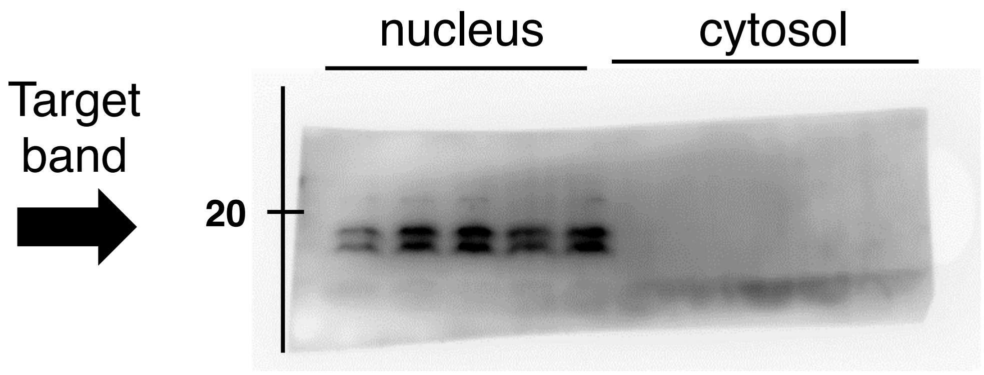

3rd

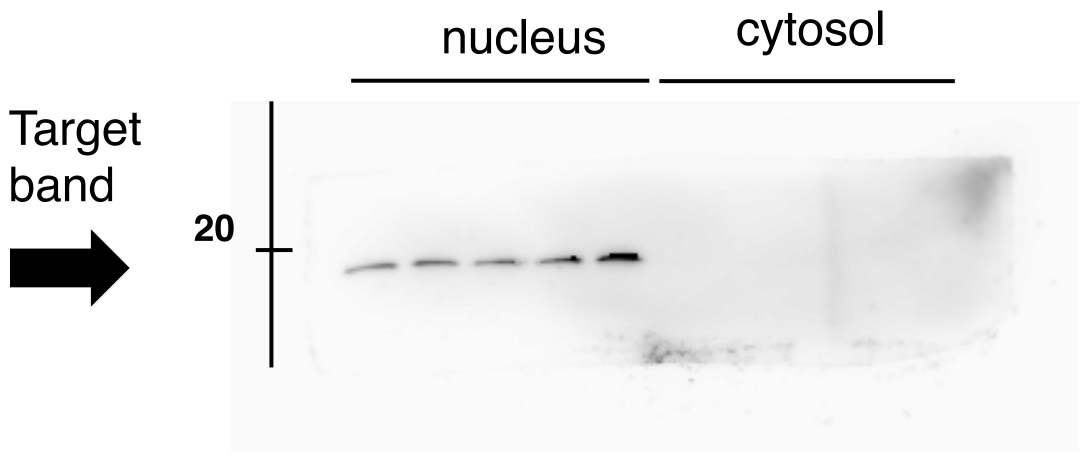

The order of the cytoplasmic and nuclear fractions is reversed in the 2nd and 3rd procedures.

Uncropped Gels and Blots images of Figure 2g were shown.

Figure 2g

Vinculin

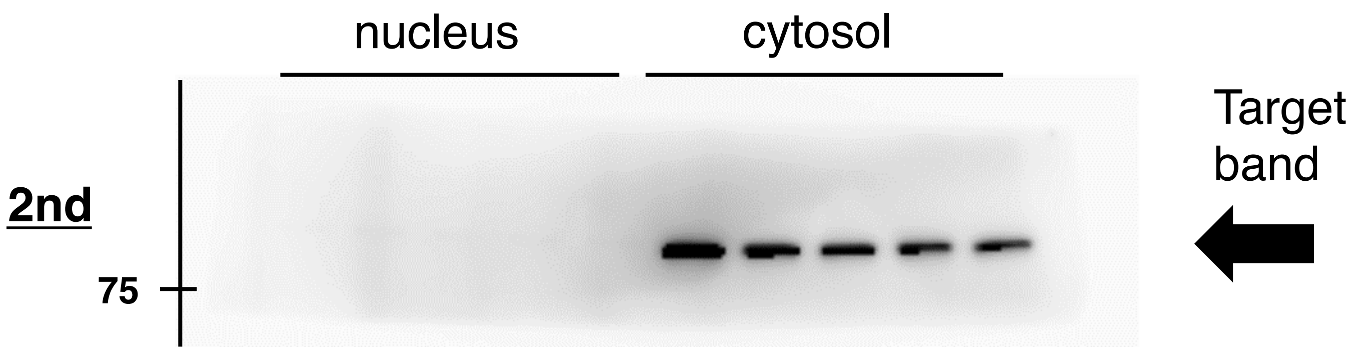

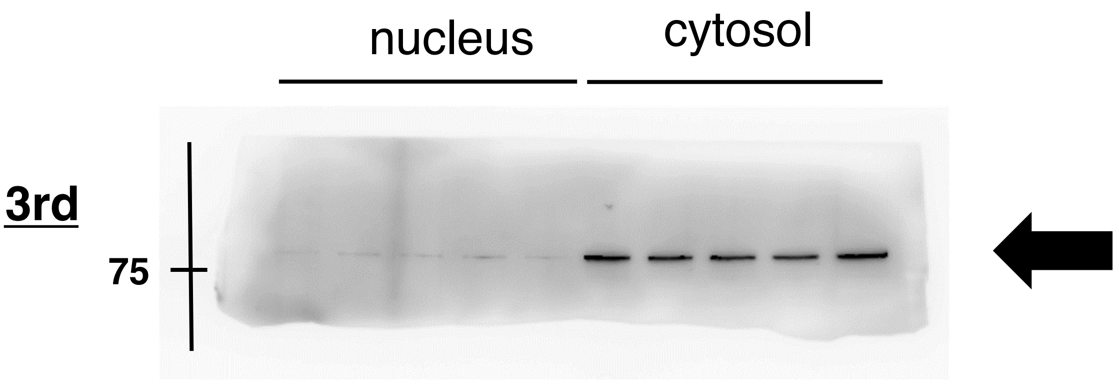

The order of the cytoplasmic and nuclear fractions is reversed in the 2nd and 3rd procedures.

Uncropped Gels and Blots images of Figure 2g were shown.

Figure 2g

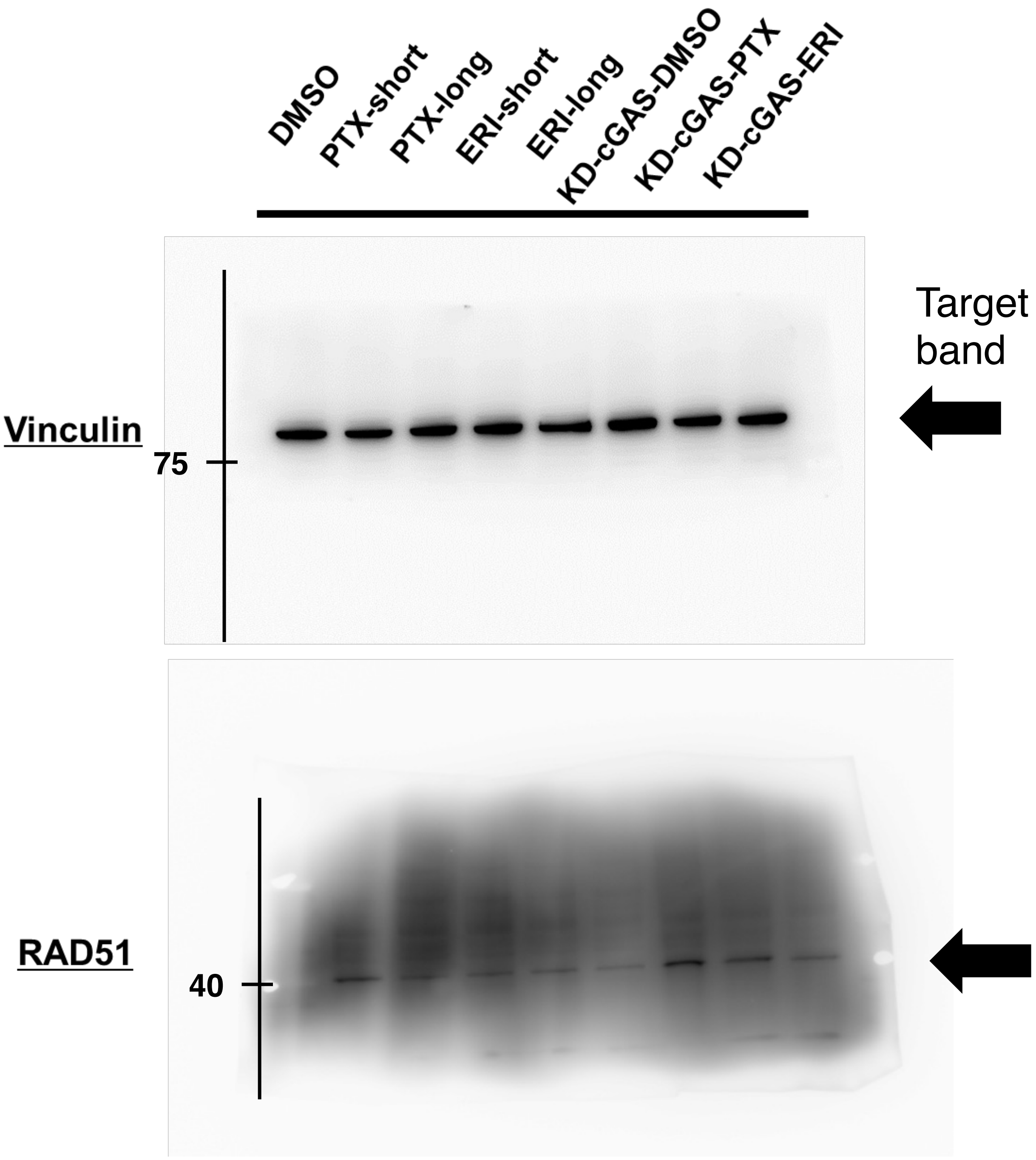

Uncropped Gels and Blots images of Figure 3c were shown.

Figure 3c

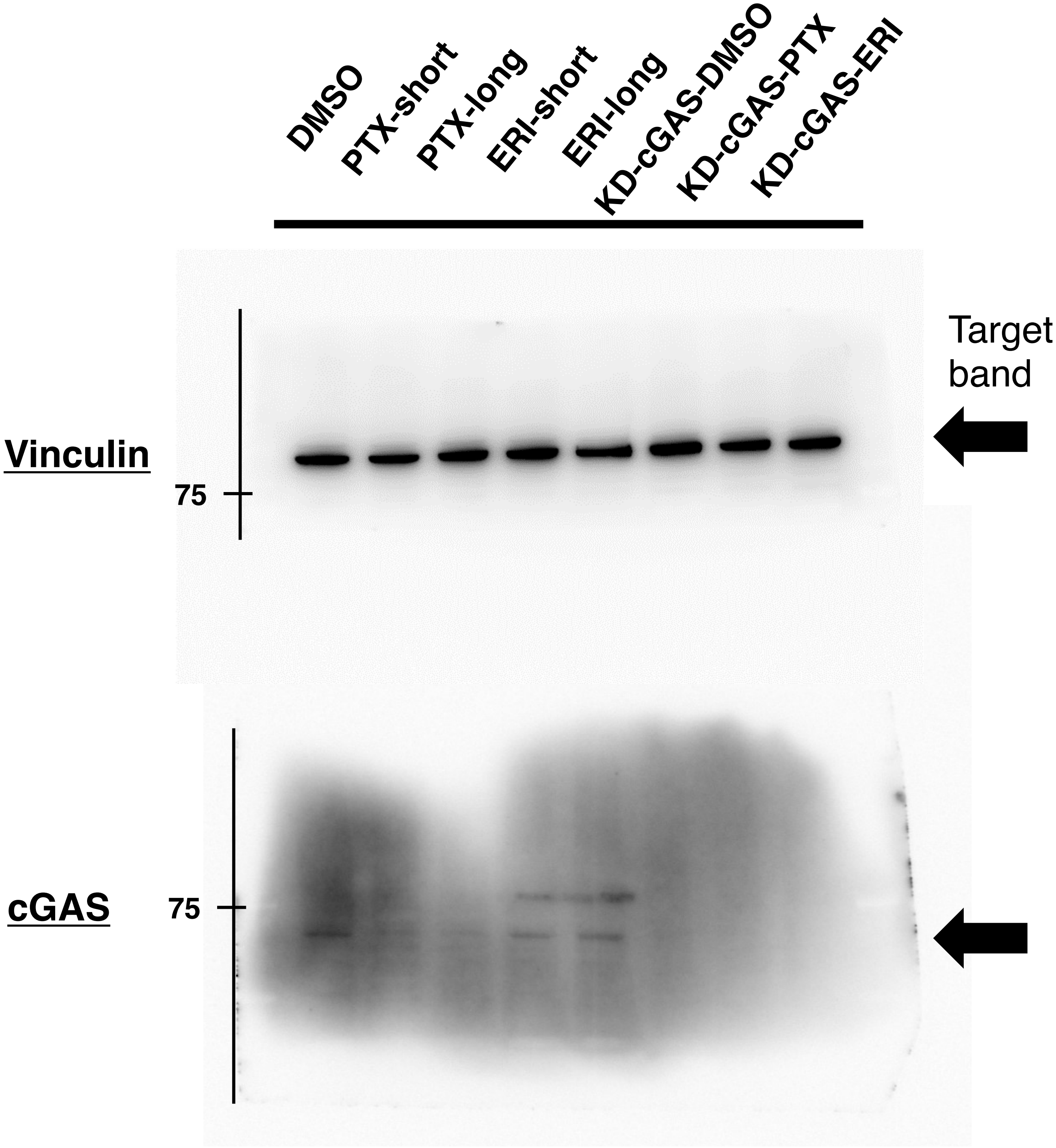

Uncropped Gels and Blots images of Figure 3c were shown.

Figure 3c

Vinculin

2nd

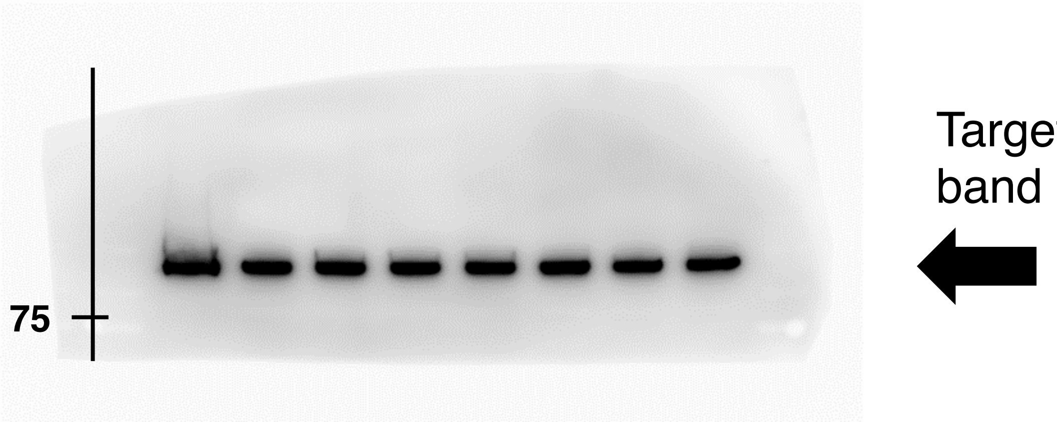

3rd

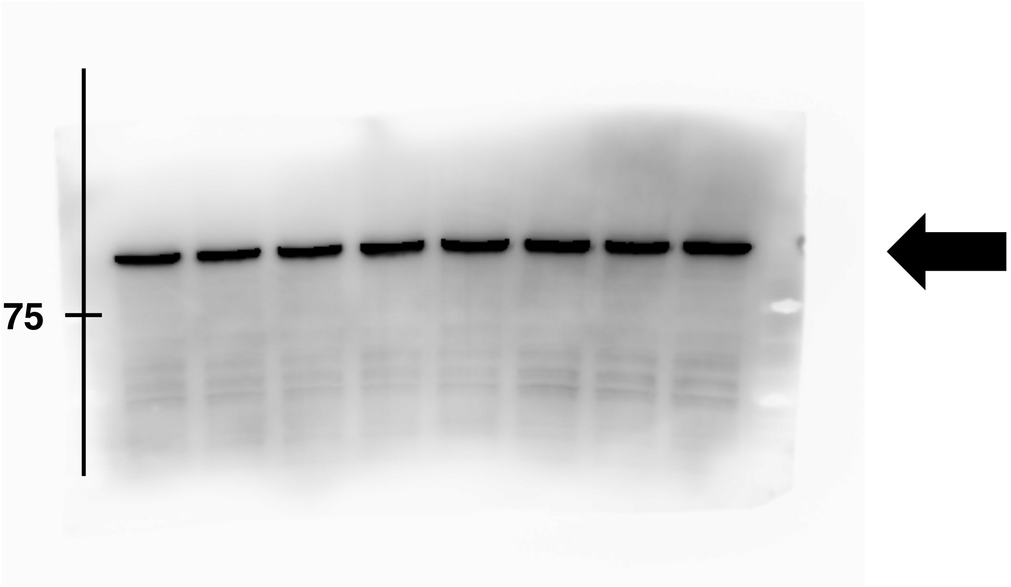

Uncropped Gels and Blots images of Figure 3c were shown.

Figure 3c

RAD51

Short exposure

long exposure

2nd

40

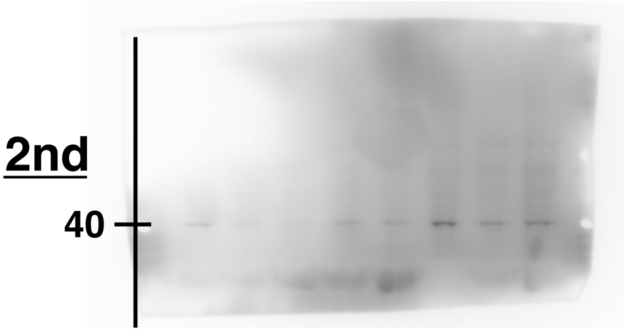

40

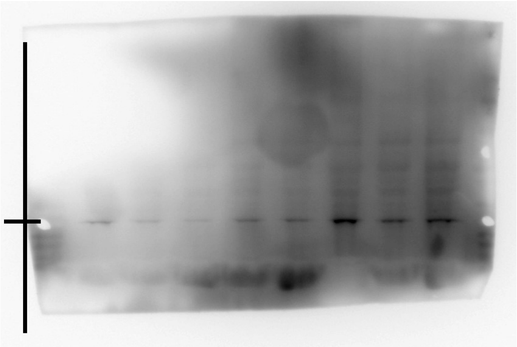

Target band

3rd

40

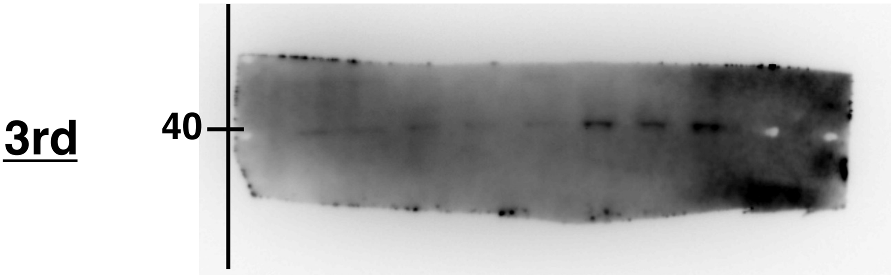

Uncropped Gels and Blots images of Figure 3c were shown.

Figure 3c

cGAS

2nd

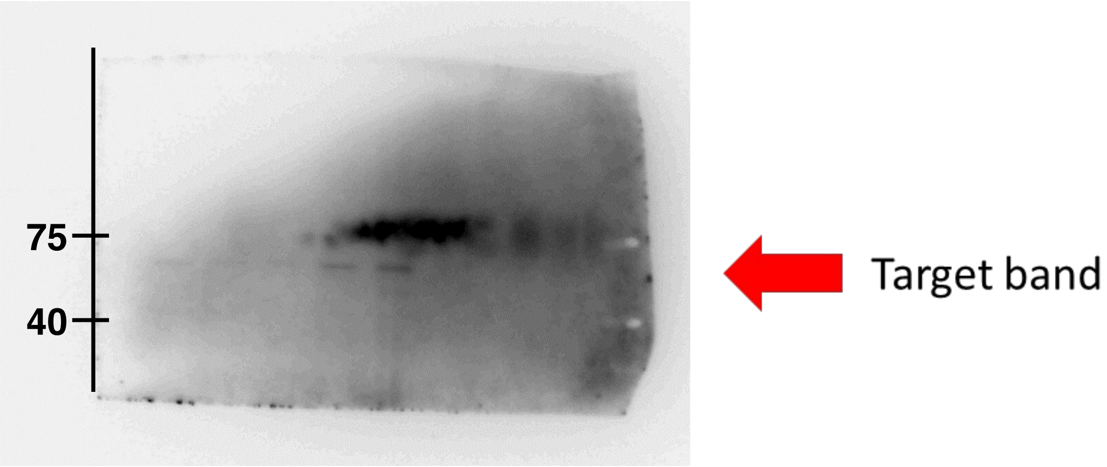

3rd

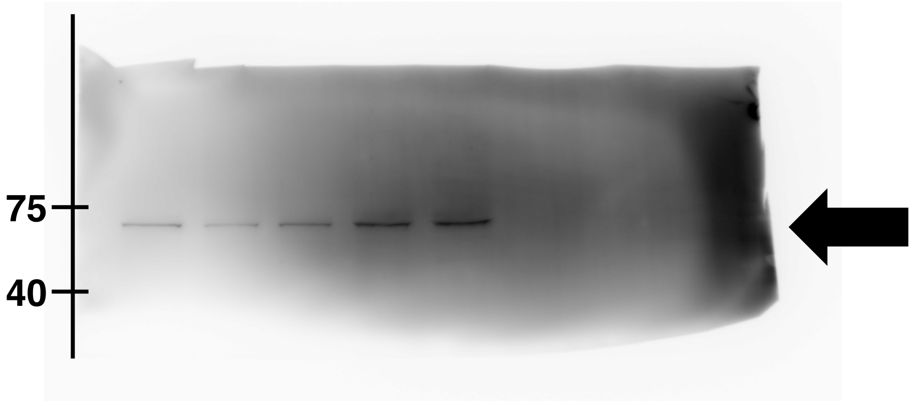

Uncropped Gels and Blots images of Figure 2g were shown.

Figure 3c

a **cGAS**

1st

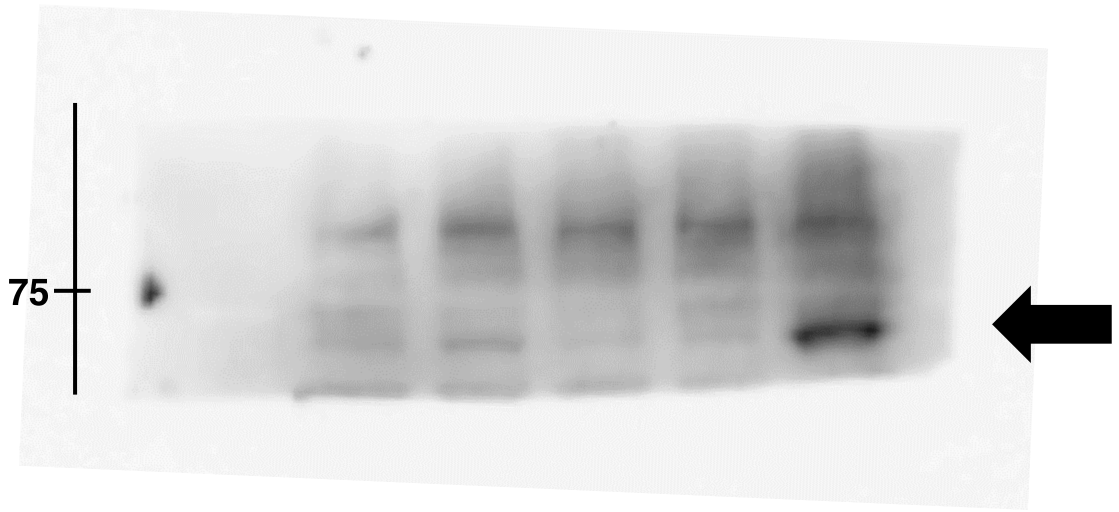

Histone H3

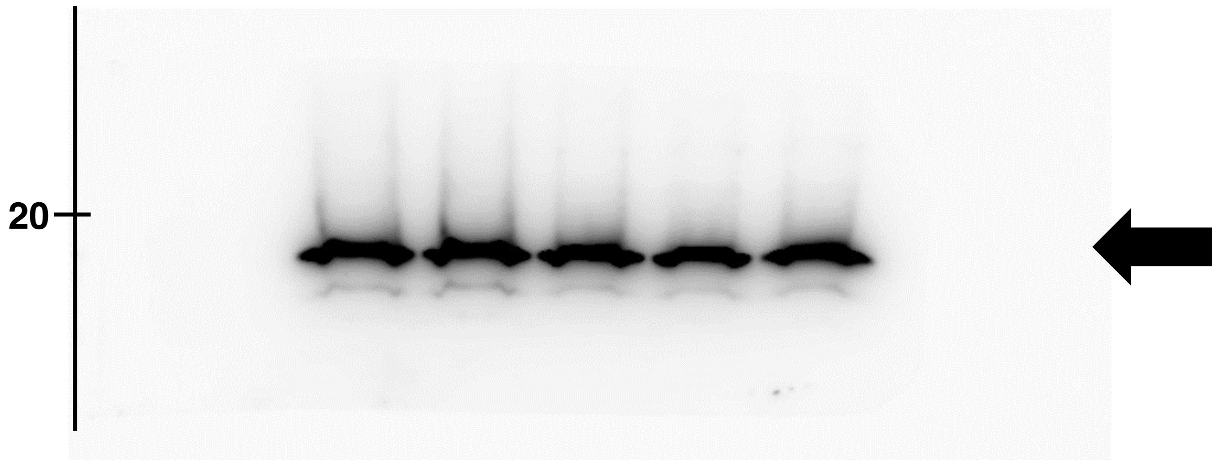

Uncropped Gels and Blots images of Supplementary Figure 2 were shown.

Supplementary Figure 2

cGAS

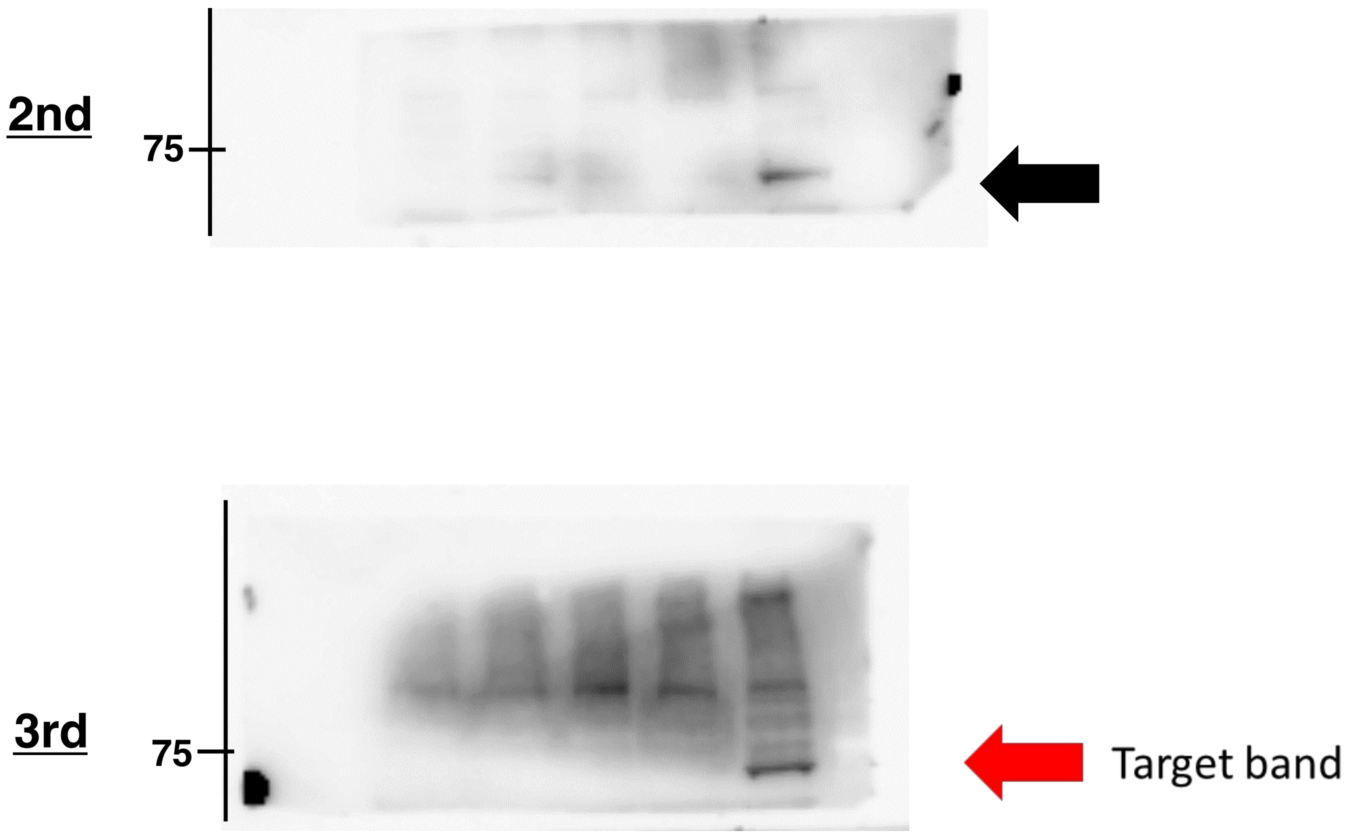

Uncropped Gels and Blots images of Supplementary Figure 2 were shown.

Supplementary Figure 2

Histone H3

2nd

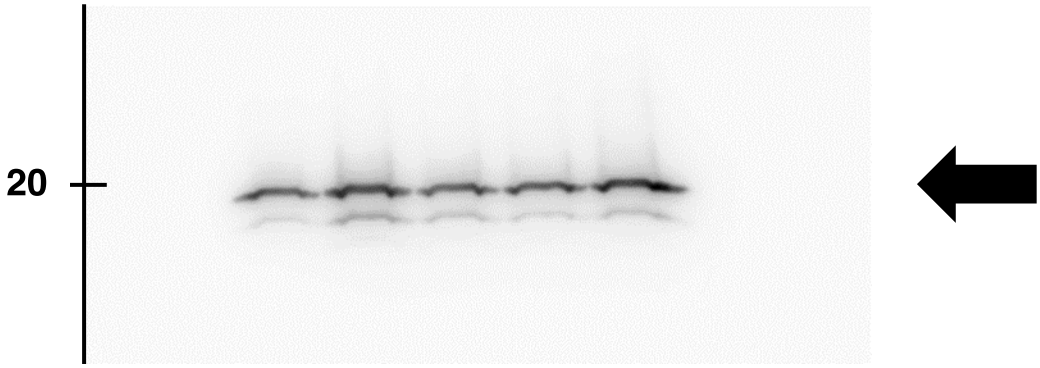

3rd

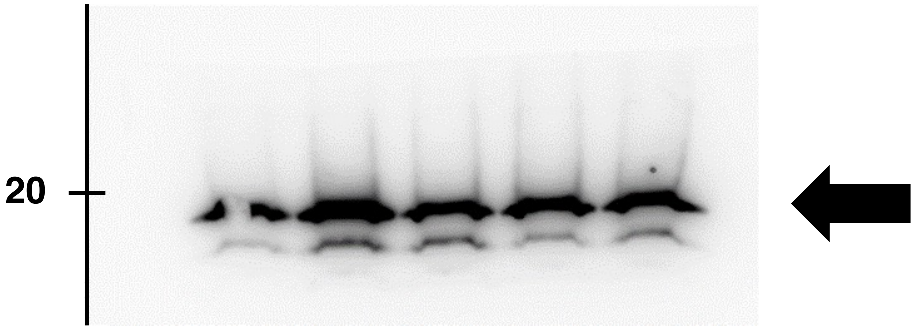

Uncropped Gels and Blots images of Supplementary Fig 2 were shown.

Supplementary Figure 2

Supplemental figure regarding what reviwer pointed out

- As pointed out by the Reviewer, the scale bars in the images were found to be incorrect. IF and IHC images were checked and scale bars were corrected.

Figure 2a

Figure 2a (before revise)

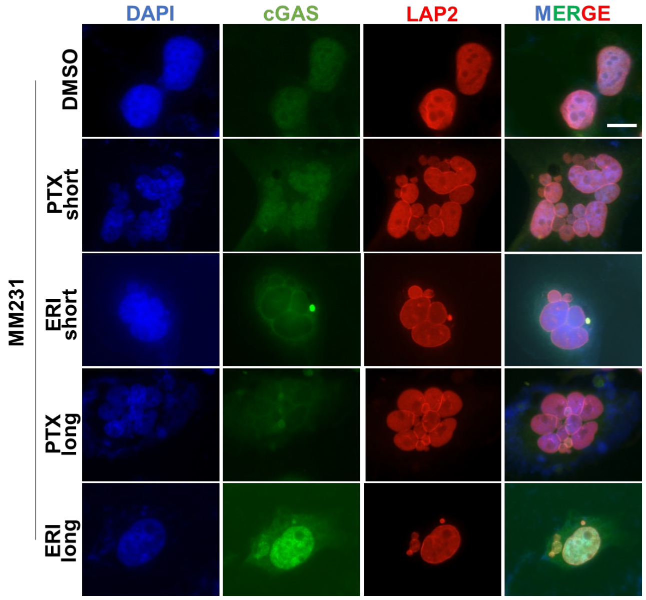

Figure 2a (after revise)

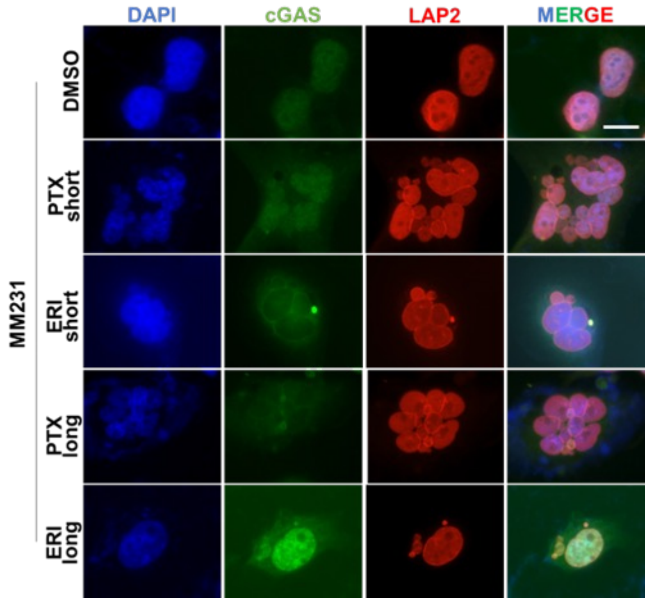

We fixed scale bar. The white line is 10µm.

Figure 2b

Figure 2b (before revise)

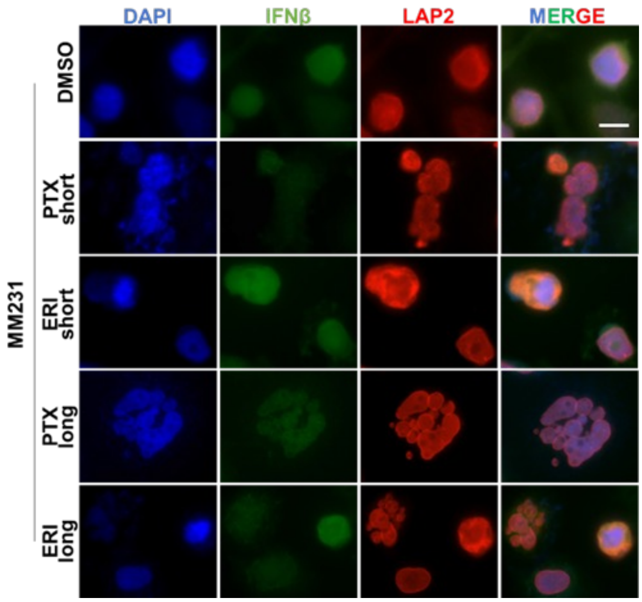

Figure 2b (after revise)

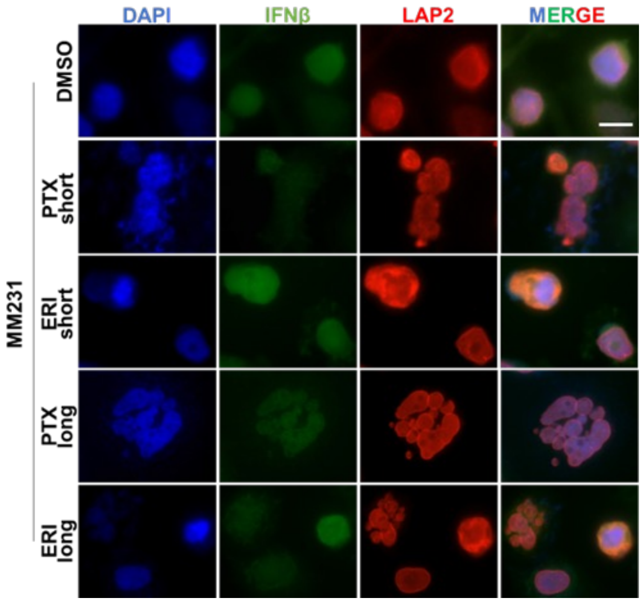

We fixed scale bar. The white line is 10μm.

Figure 2c

Figure 2c (after revise: with higher exposure)

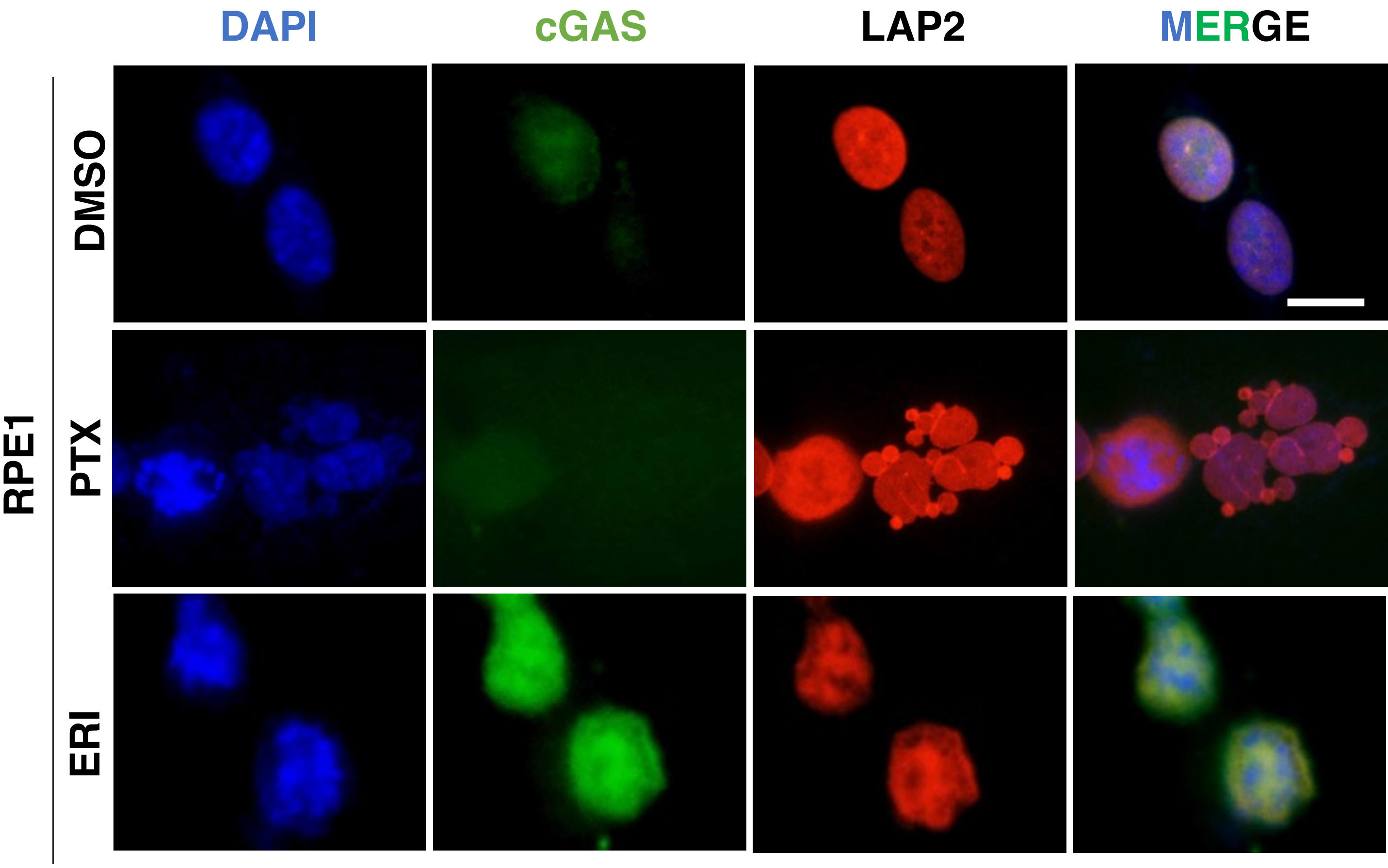

An image gain-adjusted figure is attached after the images were taken (above). We fixed scale bar. The white bar is 10µm.

We would like to change Figure 2c to these photos.

Figure 2c (before revise: lower exposure)

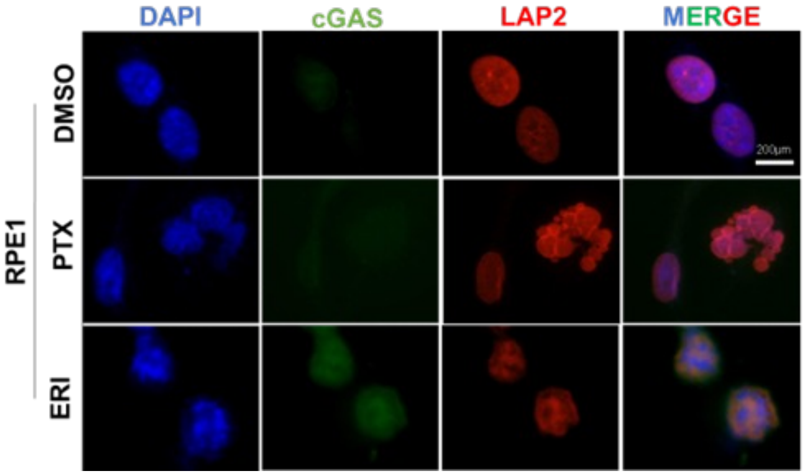

Figure 2d

Figure 2d (after revise: higher exposure)

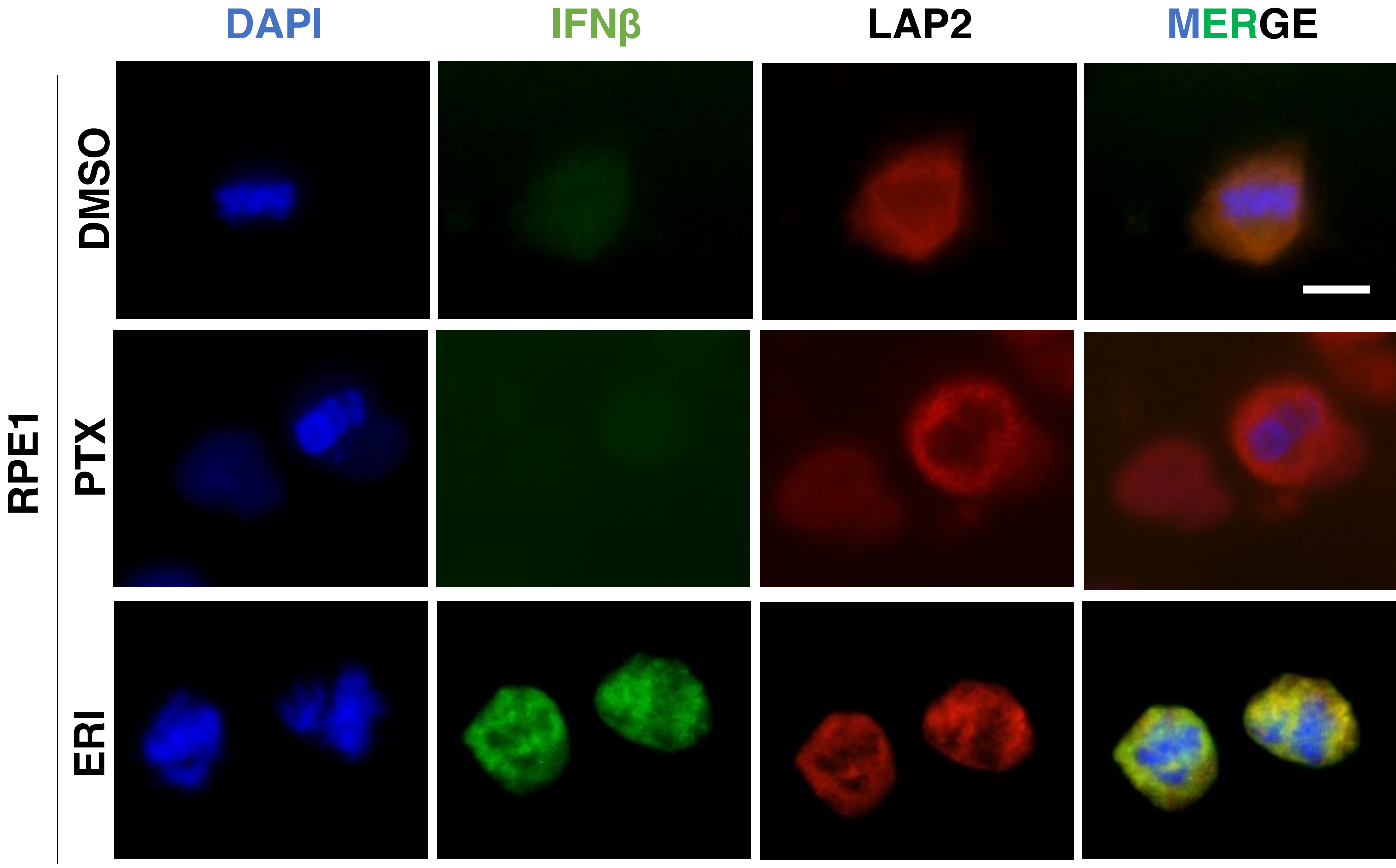

An image gain-adjusted figure is attached after the images were taken (above). We fixed scale bar. The white bar is 10μm.

We would like to change Figure 2d to these photos.

Figure 2d (before revise: lower exposure)

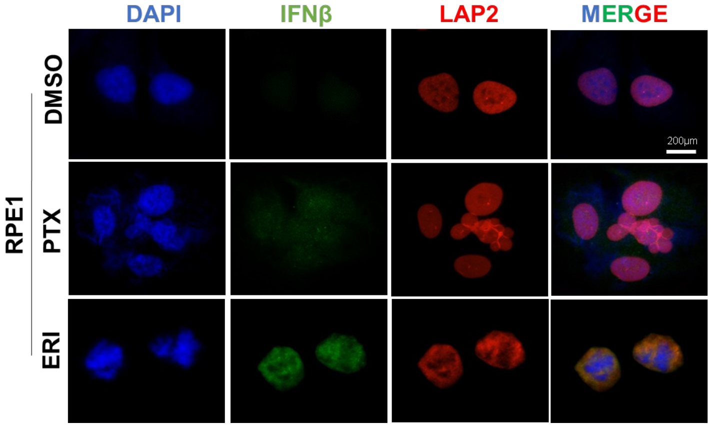

Supplementary Figure

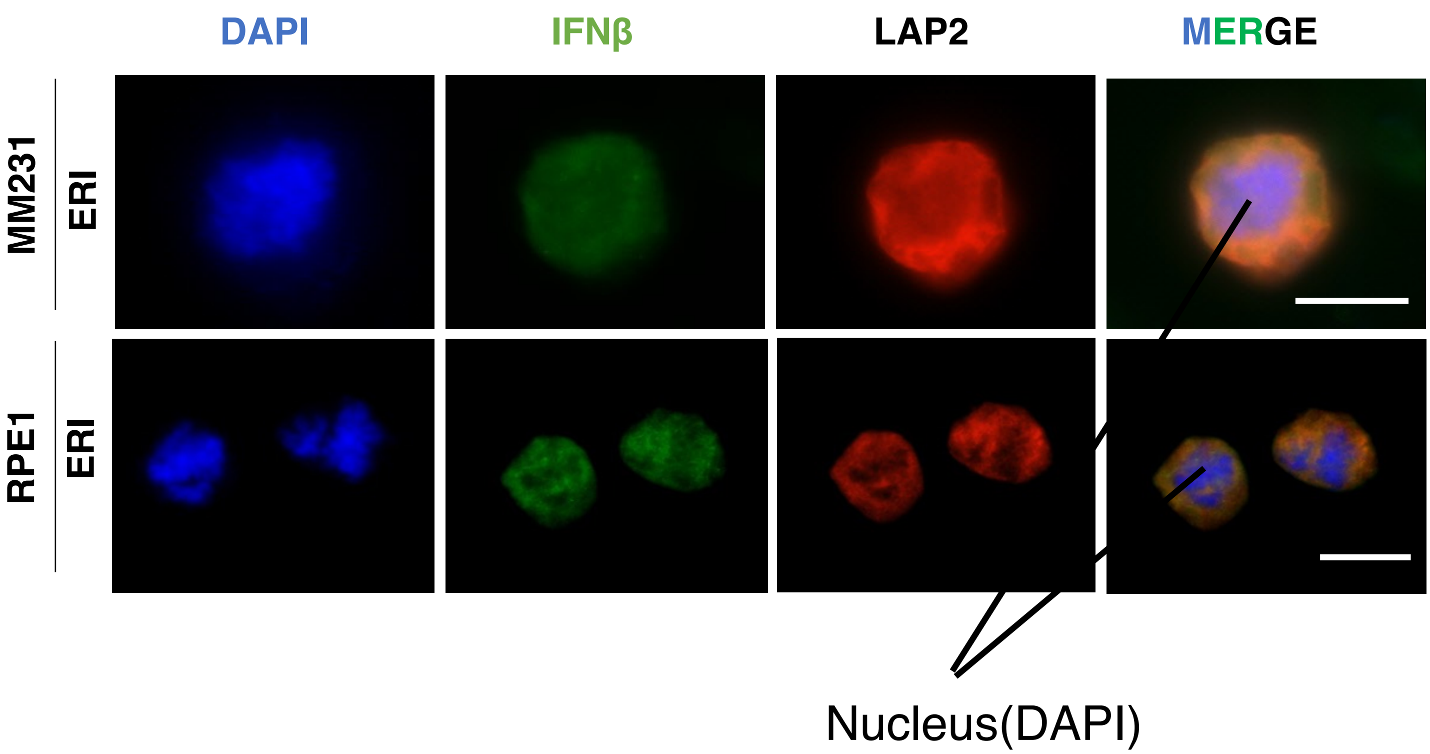

These are part of magnified images of the IFNβ-stained cells in **Figure 2b and 2d**. IFNβ was not stained in the nucleus but stained in the cytosol. The white bars are 10µm.

Additional information on figure 2b and 2d

Supplementary Figure

2nd

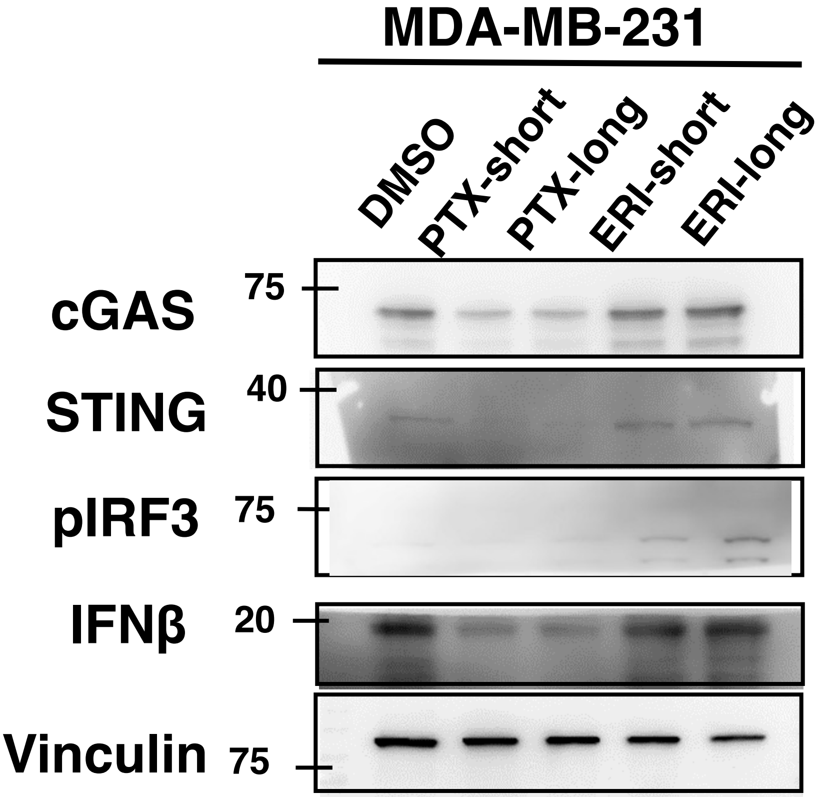

Additional information on figure 2f

3rd

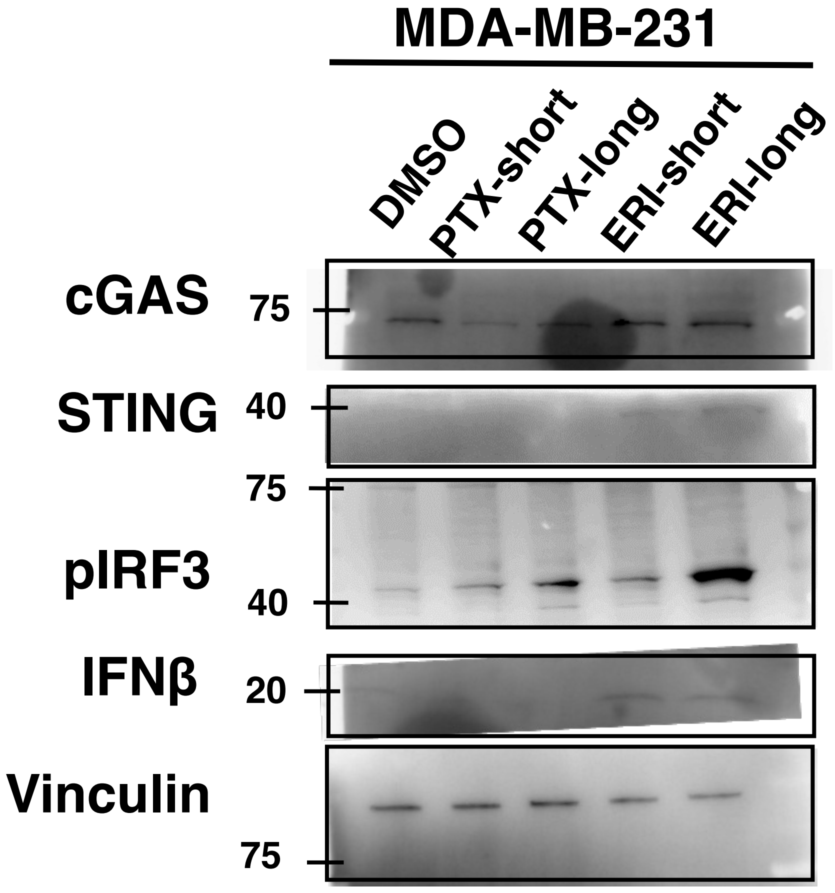

The results of other Westernblotting in figure 2f were consistent with the conclusion that cGAS/STING/pIRF3/IFNb tended to be higher in the ERI long group than in the others.

Figure 3a

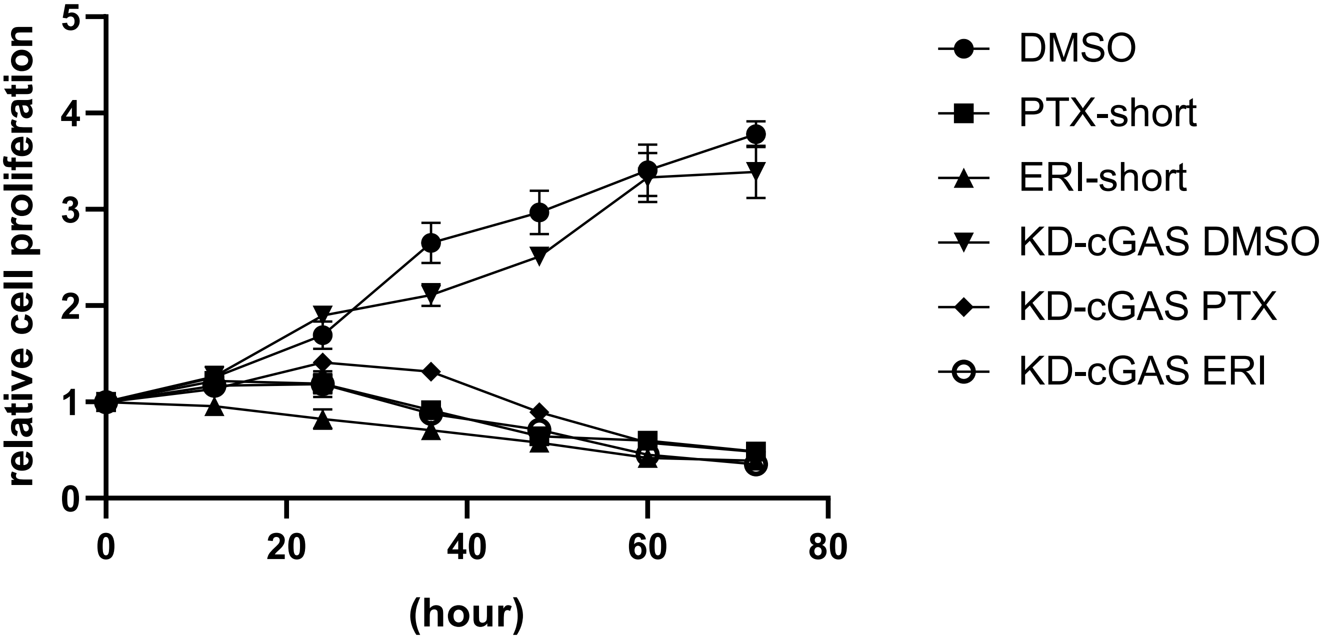

Proliferation at 0 hour was defined as 1, and the relative cell proliferation of above cells was blotted.

$$\text{Relative cell proliferation} = \frac{\text{(Mean OD value of cell A at time X)}}{\text{(Mean OD value of DMSO at 0h)}}$$

※OD: optical density

We would like to change Figure 3a to this graph.

Supplementary Figure

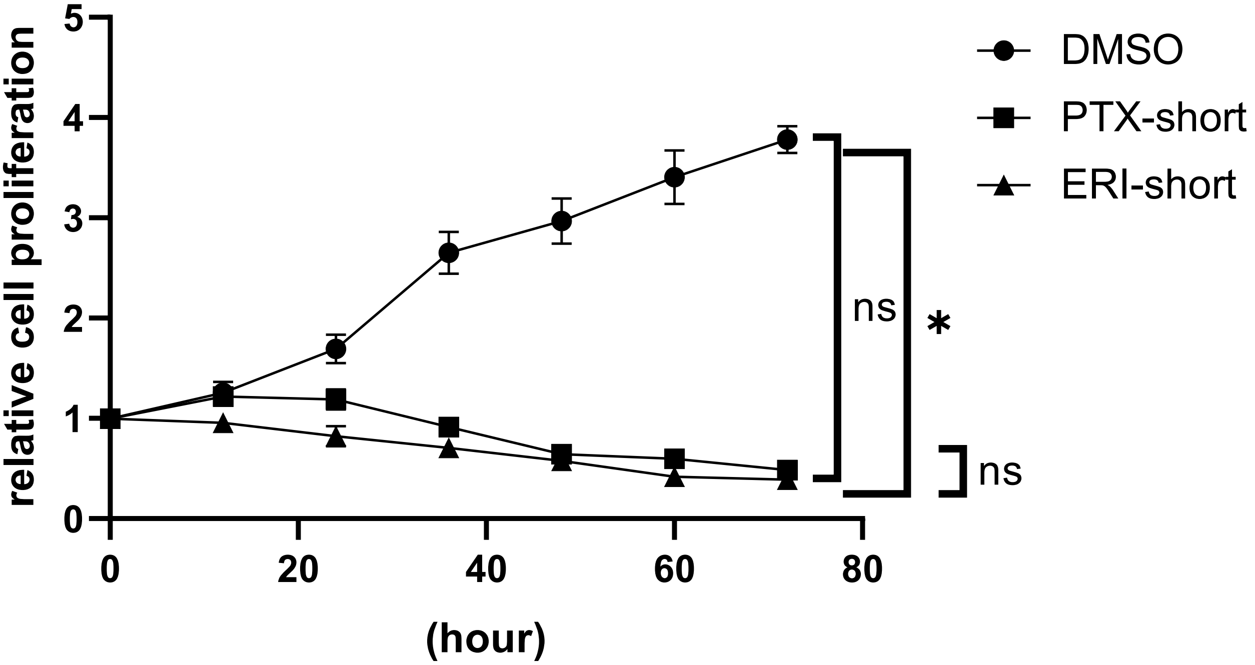

Statistical differences were evaluated by using one-way anova.

ERI-short significantly inhibited cell proliferation compared to DMSO (* p=0.047).
PTX-short inhibited cell proliferation compared to DMSO, but not significantly (p=0.1142).
There was no significant difference in the inhibition of cell proliferation between PTX-short and ERI-short (p>0.9999).

Additional information on figure 3a

Supplementary Figure

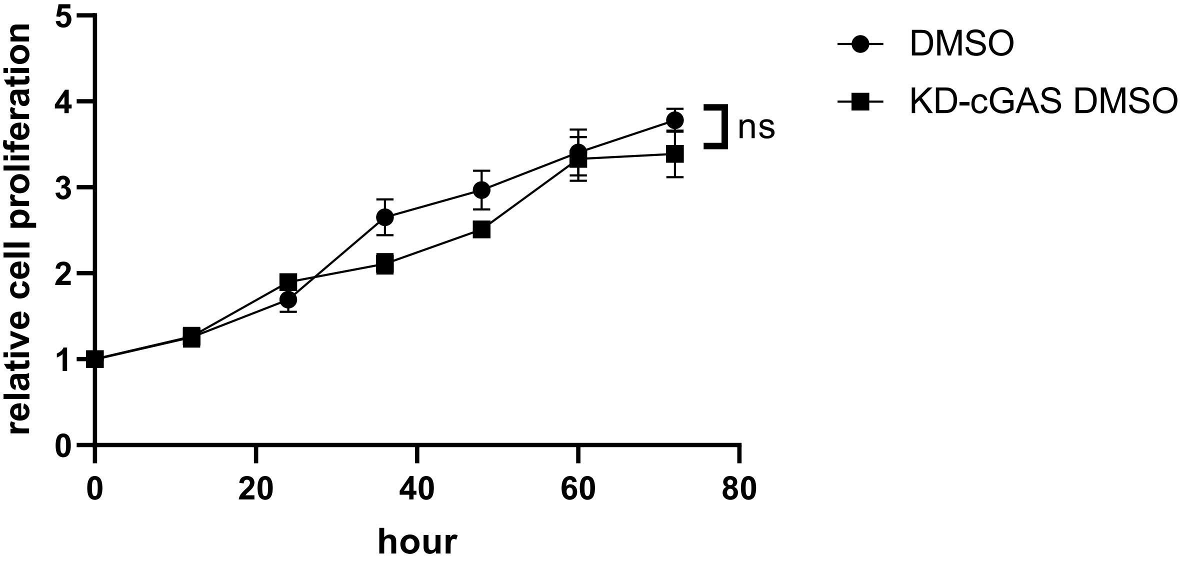

There was no significant difference between DMSO and KD-cGAS-DMSO ($p>0.9999$).

Additional information on figure 3a

Supplementary Figure

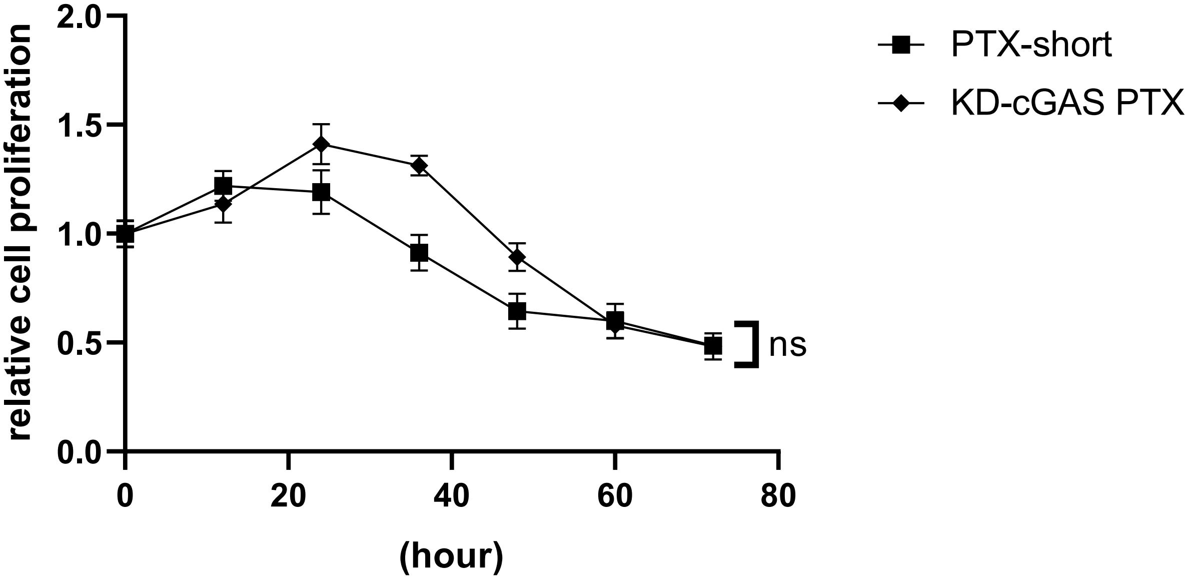

There was no significant difference between PTX-short and KD-cGAS-PTX (p>0.9999).

Additional information on figure 3a

Supplementary Figure

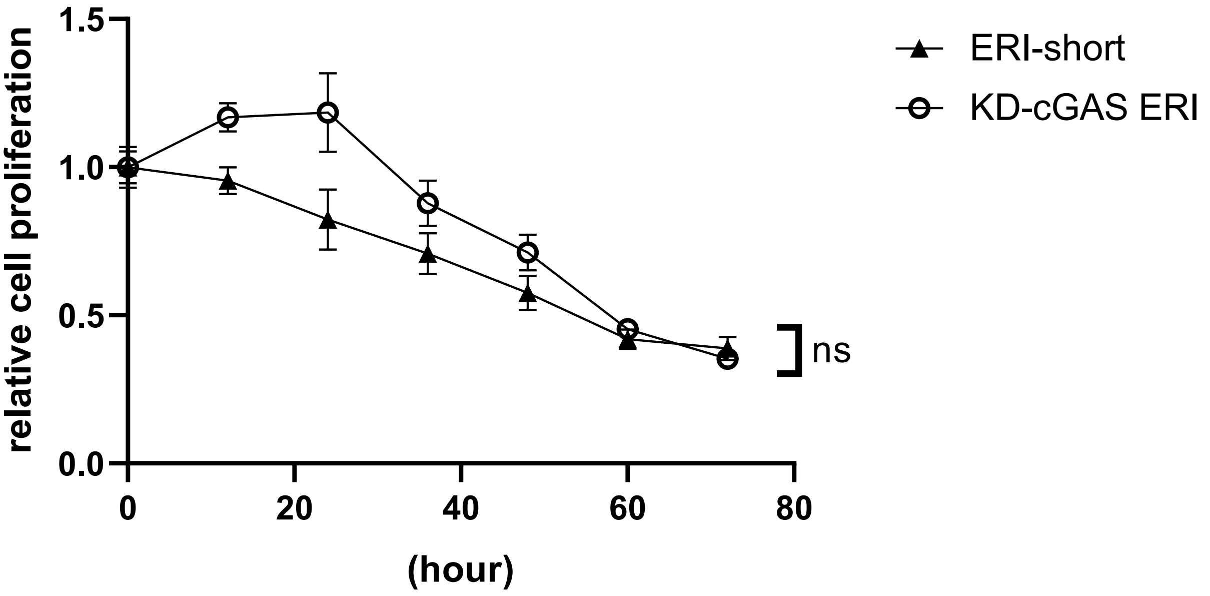

There was no significant difference between ERI-short and KD-cGAS-ERI (p>0.9999).

Additional information on figure 3a

Supplementary Figure

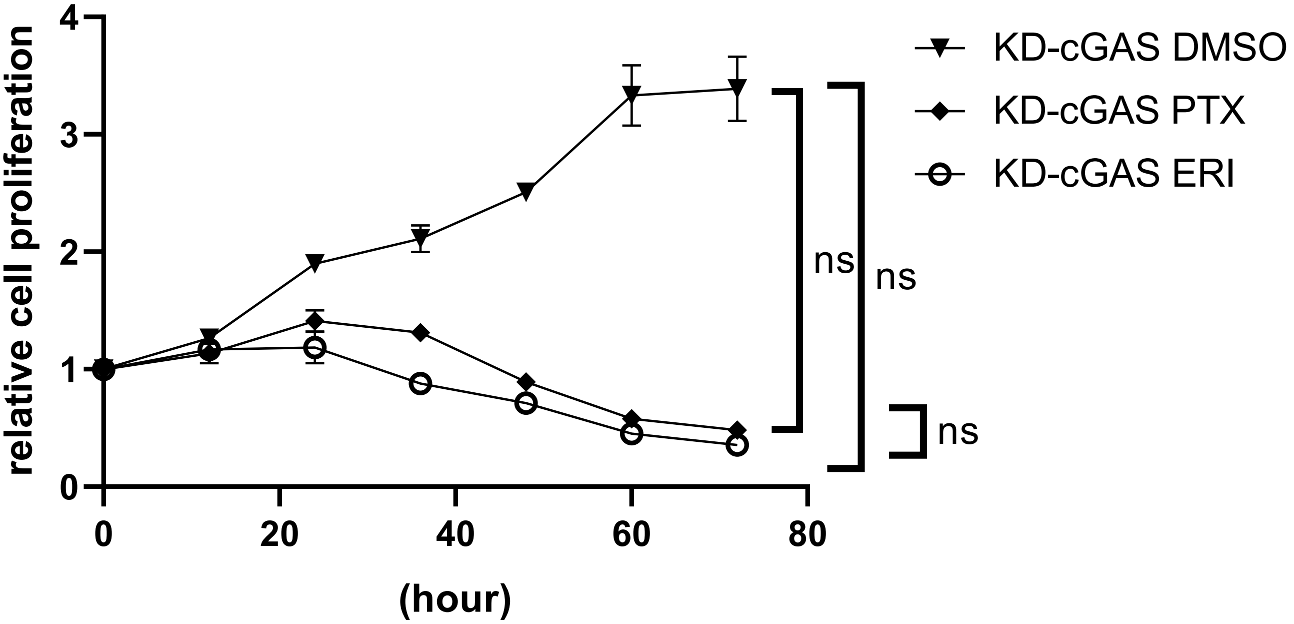

KD-cGAS ERI (p=0.0526) and KD-cGAS PTX (p=0.4404) inhibited cell proliferation compared to KD-cGAS DMSO, but not significantly.
There was no significant difference in the inhibition of cell proliferation between KD-cGAS PTX and KD-cGAS ERI (p>0.9999).

Additional information on figure 3a

Figure 3b

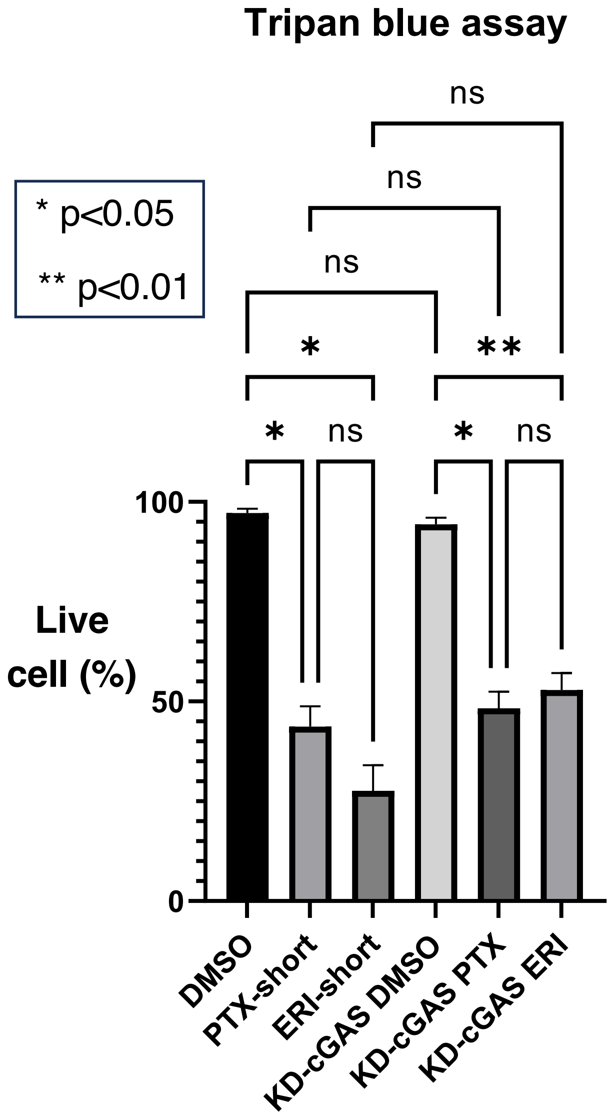

The results of the statistical study are shown.

The percentage of viable cells was statistically compared by unpaired t test.

DMSO and KD-cGAS DMSO were not significantly different in the percentage of viable cells (p=0.3967).

PTX and KD-cGAS PTX were not significantly different in the percentage of viable cells (p=0.5926).

KD-cGAS ERI tended to have a higher percentage of viable cells than ERI short, but the difference was not significant (p=0.0650).

We would like to change Figure 3b to this graph.

Figure 3c

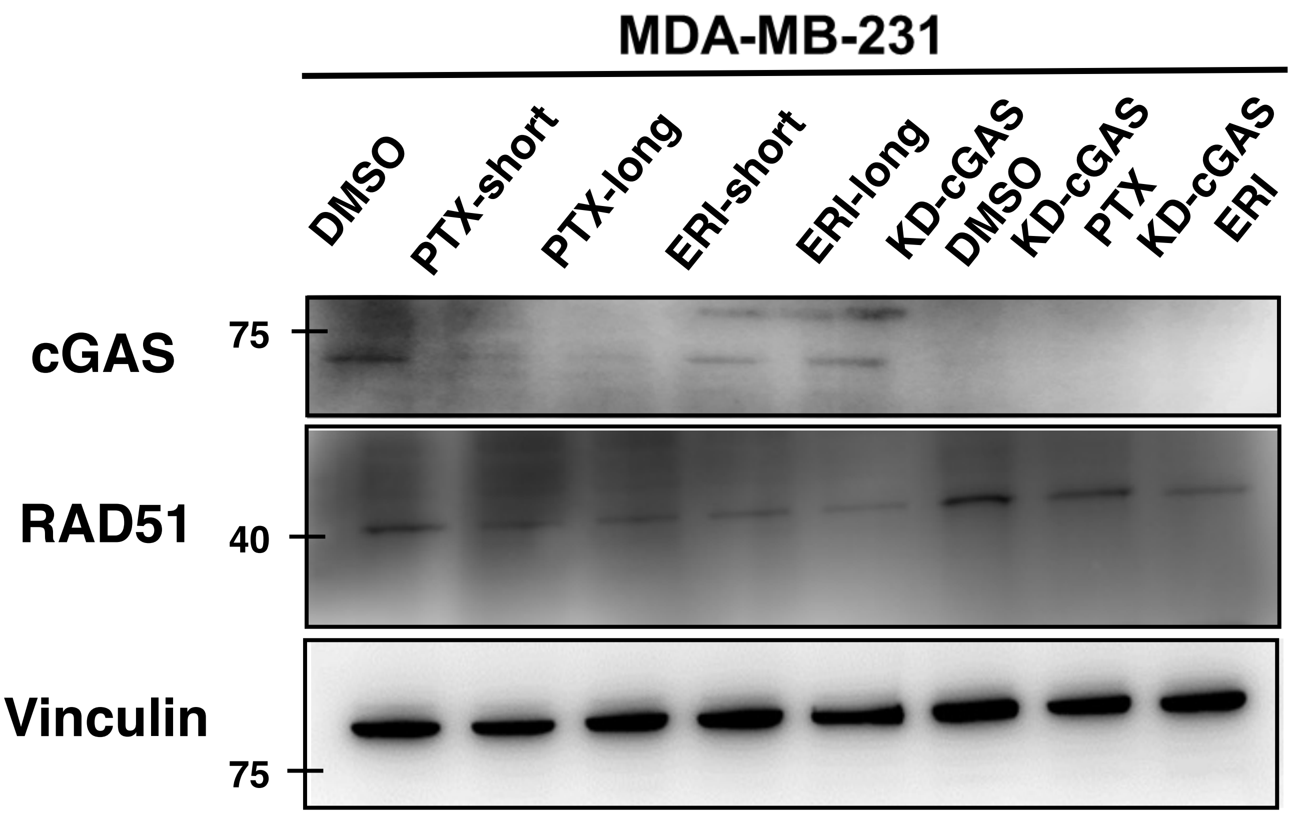

The data shows that cGAS is no longer elevated by ERI or PTX treatment after knockdown of cGAS, and Rad51 is elevated at the same level as in the control in both cases.

We would like to change Figure 3c to this one.

Figure 3d

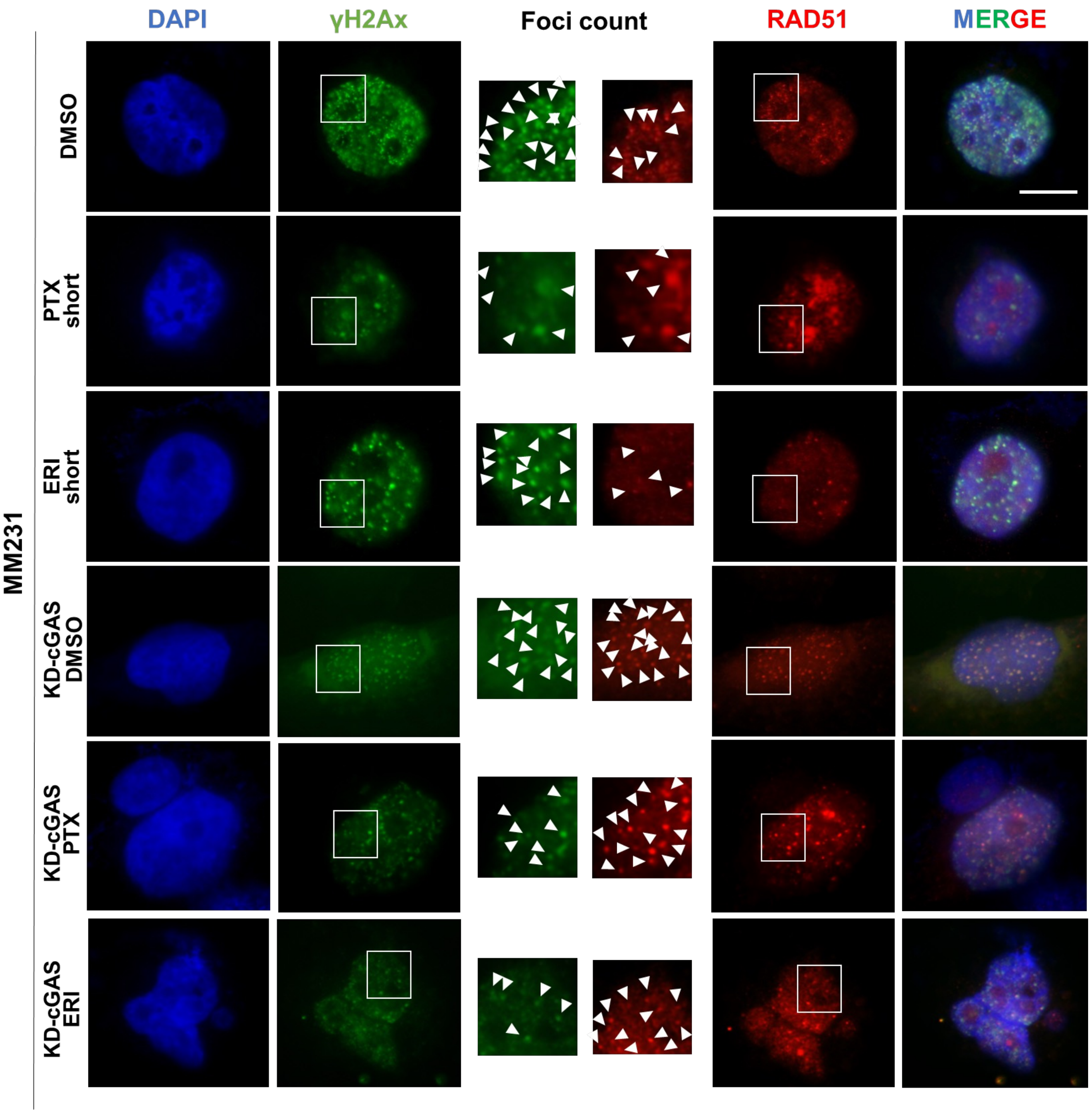

The photos in Figure 3d (**Submitted two times before**) were not sufficient for evaluation of foci, especially since it was diffusely stained with RAD51. White triangles point to foci. We fixed scale bar. The white bar is 10μm.

We would like to change Figure 3d to this one.

Figure 4b

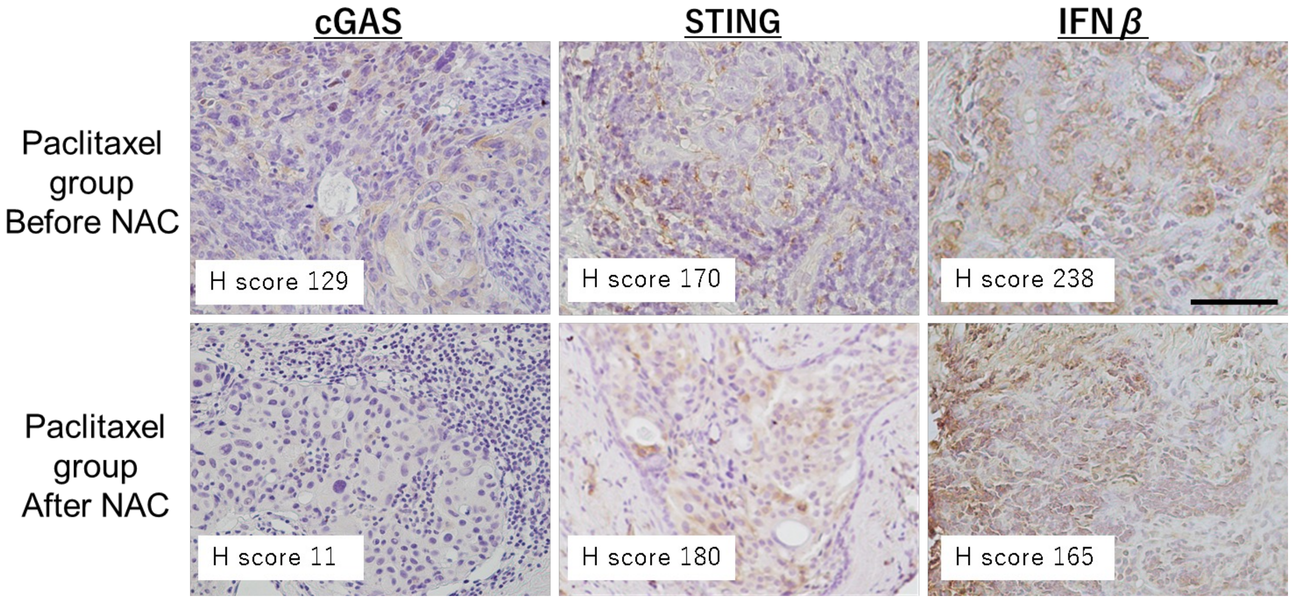

Figure 4c

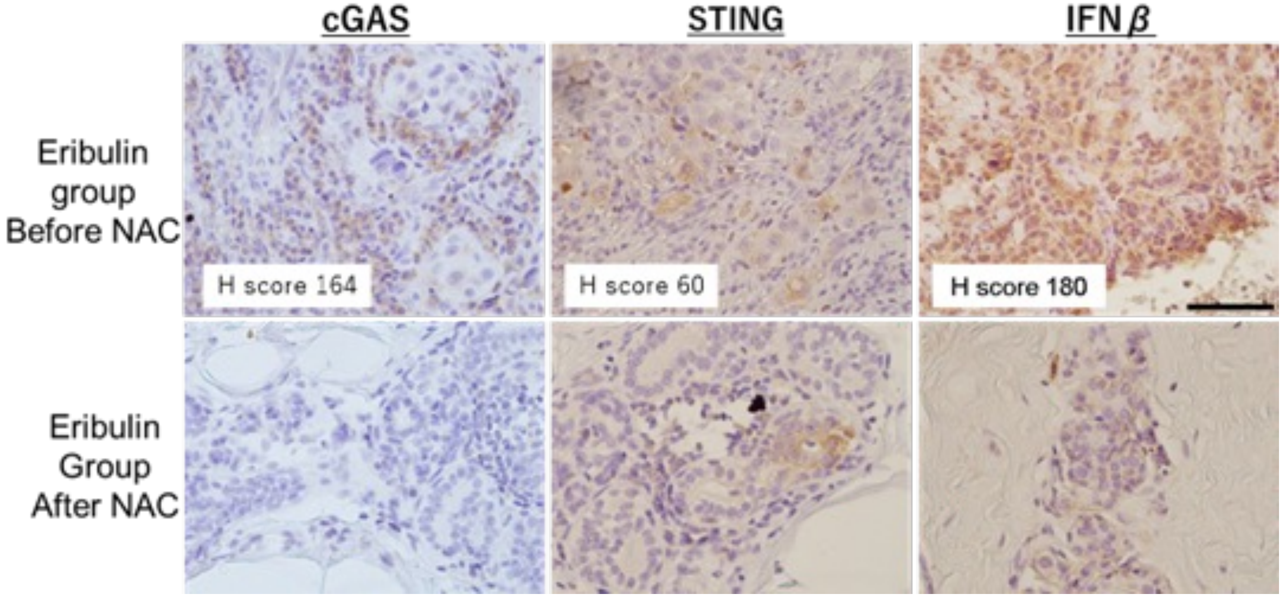

We fixed scale bar. The black bar is 100μm.

We would like to change Figure 4b and 4c to this one.

Supplementary Figure

(×400)

Case 1

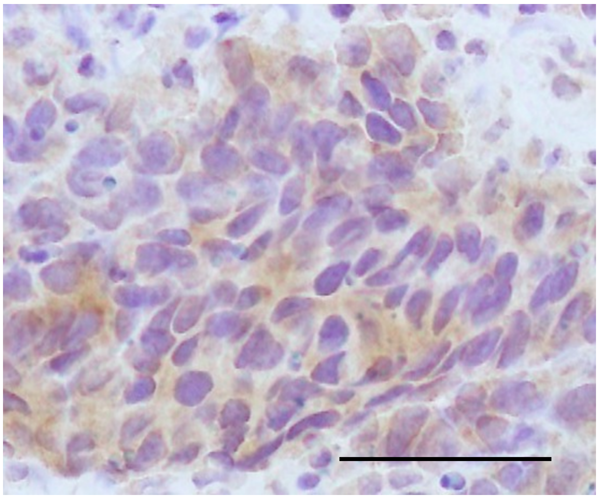

Case 2

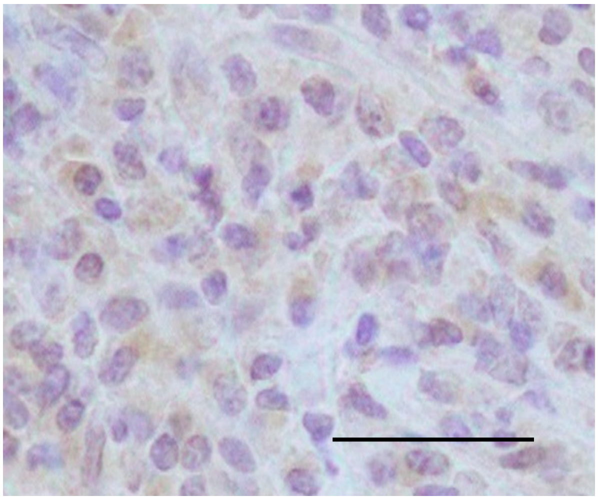

Case 3

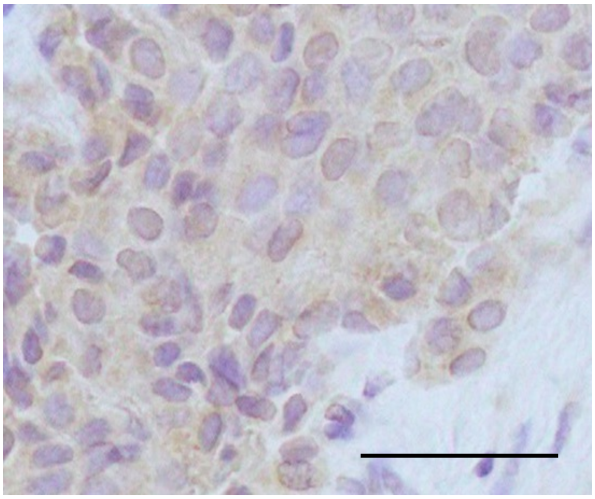

There are three images with IFNβ stained in the cytoplasm rather than the nucleus in IHC.

The black lines are 100μm.

Additional information on figure 4b, 4c

Supplementary Figure 2c

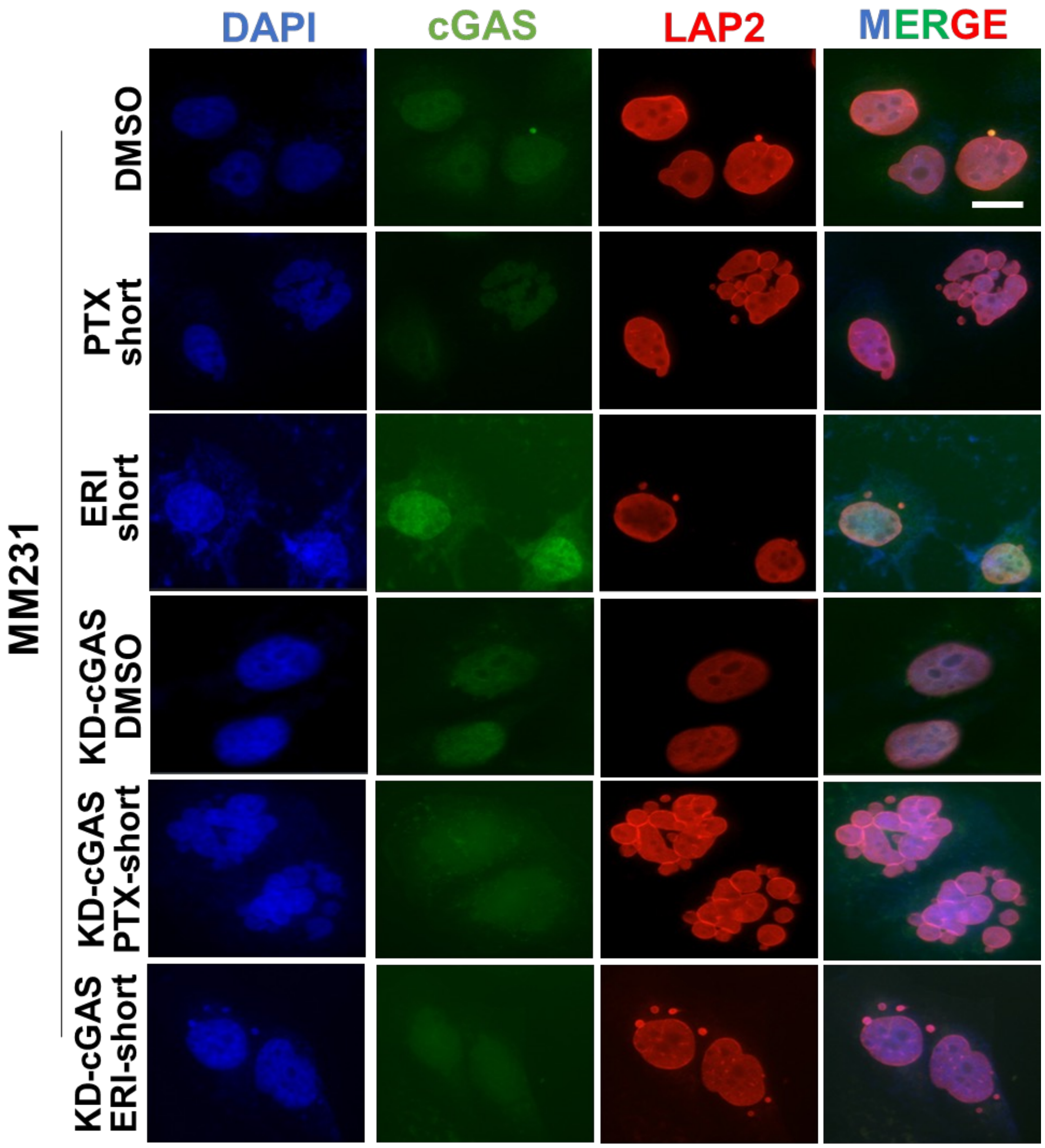

cGAS staining in Figure 2 seems to be a cGAS-specific finding because the staining of the cells seems to disappear when cGAS is knocked down as shown in **supplementary Fig.2c**. The white line is 10µm.
